# Functional characterisation of missense ceruloplasmin variants and real-world prevalence assessment of Aceruloplasminemia using population data

**DOI:** 10.1016/j.ebiom.2025.105625

**Published:** 2025-03-04

**Authors:** Nicole Ziliotto, Sara Lencioni, Martina Cirinciani, Alan Zanardi, Massimo Alessio, Giulia Soldà, Eleonora Da Pozzo, Rosanna Asselta, Andrea Caricasole

**Affiliations:** a Department of Pharmacy, University of Pisa, Via Bonanno 6, Pisa 56126, Italy; b Department of Research & Innovation, Kedrion Biopharma S.p.A, Via di Fondovalle, Loc. Bolognana, Gallicano 55027, Italy; c Proteome Biochemistry, COSR-Centre for Omics Sciences, IRCCS Ospedale San Raffaele, Via Olgettina 60, Milano 20132, Italy; d Department of Biomedical Sciences, Humanitas University, Via Rita Levi Montalcini 4, Pieve Emanuele, 20072, Italy; e IRCCS Humanitas Research Hospital, Via Manzoni 56, Rozzano 20089, Italy

**Keywords:** Aceruloplasminemia, Ceruloplasmin, Genetic prevalence, Functional analysis, Rare disease

## Abstract

**Background:**

Aceruloplasminemia (ACP) is a rare recessive disease caused by loss of ceruloplasmin activity due to pathogenic variants in the ceruloplasmin (*CP*) gene. ACP causes iron accumulation in various organs, leading to neurodegeneration, anaemia, and diabetes. Estimating ACP prevalence is challenging, particularly as missense variants are not readily identified as pathogenic.

**Methods:**

Heterozygous missense variants likely to impact function were mapped in gnomAD and representative examples analysed for effects on CP activity. This knowledge was complemented by prediction of destabilizing effects of potentially pathogenic missense variants and integrated with loss-of-function mutations. Global ACP prevalence was predicted and compared with a more traditional method.

**Findings:**

Several as yet uncharacterised missense CP variants of pathogenic interest were identified by structure-function *in-silico* analysis. A representative subset was functionally validated, together with known ACP missense variants. Insights on the relative importance of copper ions coordinating centres in CP and its substrate specificity were discovered. Overall, a destabilizing effect was predicted for 130 missense *CP* variants. This information, integrated with known ACP missense and loss-of-function CP variants in gnomAD, allowed an estimation of ACP prevalence of 12.6/10^6^. An alternative analysis based on minor allele frequency ≤0.01 resulted in an ACP prevalence as high as 8/10^6^.

**Interpretation:**

These prevalence estimates for ACP are 20–25-fold higher than previously estimated and underscore the applicability of structure-function based analyses of real-world genetic variability to provide an alternative method for representing the frequency of rare disease variants.

**Funding:**

REACT-EU PON 2014–2021, Kedrion S.p.A.

**Research in context**

**Evidence before this study**

Aceruloplasminemia (ACP) was initially identified in Japan in 1987, and its prevalence, based on a small sample of the Japanese adult population, was reported in 1999 to be 0.5 × 10^6^. A more recent study on the lifetime risk of Neurodegeneration with Brain Iron Accumulation (NBIA) disorders estimated the global prevalence of ACP to be 0.4 × 10^6^ based on loss of function *CP* variants in gnomAD (*EBioMedicine* 2022; 77:103869), and a few missense variants. The underdiagnosis of ACP is perpetuated by the scarcity of studies, the dearth of public awareness, and the lack of disease-modifying treatments, which are common challenges faced by rare diseases. Identifying ACP-causing variants is critical for both diagnostic and research purposes, but the absence of functional analyses leads to a significant proportion of variants being designated as variants of uncertain significance in genomic databases. By assessing and integrating this information effectively, the prevalence of ACP in the population can be significantly enhanced.

**Added value of this study**

The present study assessed missense variants associated with ACP by simultaneously examining their expression-related and functional characteristics in comparison with as yet uncharacterised candidate pathogenic mutants present in population genome databases. The present study demonstrates that even residues associated with Cu^2+^ type I ion coordination are critical for the enzymatic activity of CP, thereby broadening our understanding of this protein. Overall, our study created a rational flowchart that incorporated functional characterisation to validate *in-silico* structural analyses and predict mutant pathogenicity, with the ultimate goal of assessing ACP the prevalence in a real-world data population. This research represents a substantial advancement in comprehending the genetic variability of CP in relation to ACP susceptibility and increases estimates of ACP in the general population by up to 25-fold relative to current values.

**Implications of all the available evidence**

This study emphasizes the importance of employing a comprehensive panel of laboratory tests for accurate diagnosis, which includes the assessment of the specific activity (U/g) of CP. Our findings highlight the need for increased awareness in clinical practice, as they can lead to the timely diagnosis of ACP and potentially facilitate access to future disease-modifying treatments for patients who might otherwise remain undiagnosed. This study provides a more accurate prevalence of the disease, which is crucial for current efforts to develop a protein replacement therapy for ACP, as well as a blueprint for revisiting rare disease prevalence from large scale genomic datasets based on available structure-functional information of relevant proteins.

## Introduction

Aceruloplasminemia (ACP) belongs to a heterogeneous group of rare inherited diseases collectively defined as Neurodegeneration with Brain Iron Accumulation (NBIA) disorders, in which abnormal iron accumulation in the brain is a common feature.^1^ ACP is a rare autosomal recessive disease caused by pathogenic variants in the *CP* gene which encodes Ceruloplasmin (CP), the main plasma ferroxidase protein in vertebrates.^1^, ^2^, ^3^, ^4^ CP is a multi-copper (Cu^2+^)-dependent enzyme, and also functions as the principal Cu^2+^ storage protein in plasma.^4^^,^^5^ The clinical manifestations of ACP include retinal degeneration, diabetes mellitus, and neurological symptoms accompanied by liver inflammation and anaemia, with high ferritin and low saturated transferrin values.^1^ Although there are no natural history studies describing ACP penetrance, at least 70% of patients diagnosed on the basis of peripheral symptoms eventually develop neurological symptoms, based on an analysis of published case studies.^6^ ACP is presently treated with iron chelators, with significant limitations in terms of tolerability profiles and the ability to address the neurological manifestations.^7^^,^^8^ As for most ultra-rare disorders, the true prevalence of ACP is likely to be underestimated. Indeed, the reported ACP prevalence of 0.5/10^6^ dates back to 1999 and relies on a single report on a small sample (n = 4990) of the Japanese population.^9^ Subsequently, a study on the lifetime risk of NBIA disorders calculated an ACP prevalence of 0.4/10^6^ in the world population and 0.6/10^6^ in the non-Finnish European population.^10^ These results were obtained by considering all the variants classified as “pathogenic” or “probably pathogenic” in the ClinVar and HGMD (Human Gene Mutation Database) databases up to 2021 and included loss of function (LoF) variants from the genome aggregation database (gnomAD; https://gnomad.broadinstitute.org), but included only a small number of missense variants, many of which are known to be pathogenic.^10^ Indeed, pathogenic missense variants, which may result in compromised enzymatic activity with potentially limited impact on protein levels, can be easily overlooked by diagnostic assays centred on the measurement of CP plasma protein levels, though they represent the most common pathogenic variant type in ACP patients.^6^ Public genomic databases, such as gnomAD constitute a valuable source for identifying as yet uncharacterized ACP-causing variants when present in homozygosity or compound heterozygosity. This is especially true when these variants (particularly missense) can be functionally characterized to understand their effects.^11^^,^^12^ This information, appropriately evaluated and integrated, can considerably improve prevalence estimates. For diagnostic purposes, the correct classification of CP variants as pathogenic or benign is essential, but due to the lack of functional characterization a significant number of them are reported only as variants of uncertain significance. As with most ultra-rare diseases, the lack of sufficient epidemiological knowledge, poorly standardized diagnostic guidelines, and limited mechanistic understanding have hampered the development of therapies. Interestingly, ACP is caused by a deficiency of a plasma protein and is therefore potentially addressable with a protein replacement therapy.^13^^,^^14^ Indeed, the possibility of CP replacement therapy in ACP has been successfully demonstrated in Cp knock-out (CP-KO) mice (a preclinical model of ACP) with CP purified from human plasma.^15^^,^^16^ With the possibility of a future treatment, the ability to correctly identify and diagnose patients with ACP would not only significantly empower therapy development (e.g., through the further development of patient registries such as TIRCON, Treat Iron-Related Childhood-Onset Neurodegeneration, for NBIA disorders^17^) but also shorten patient journeys and enable access to therapy for as many patients as possible.

In this study, we rationally assessed all CP missense variants reported in gnomAD and identified variants impacting residues of known or predicted functional importance, including missense substitutions described in patients with ACP. A representative subset of these was analysed experimentally. This experimental dataset was used to validate a workflow aimed at predicting the pathogenicity of CP missense variants using an integrated suite of bioinformatic tools. Finally, aggregated prevalence estimates for LoF and potentially pathogenic missense variants for CP were determined.

## Methods

### Identification and prioritization of missense *CP* variants of interest

**Fig. 1:**
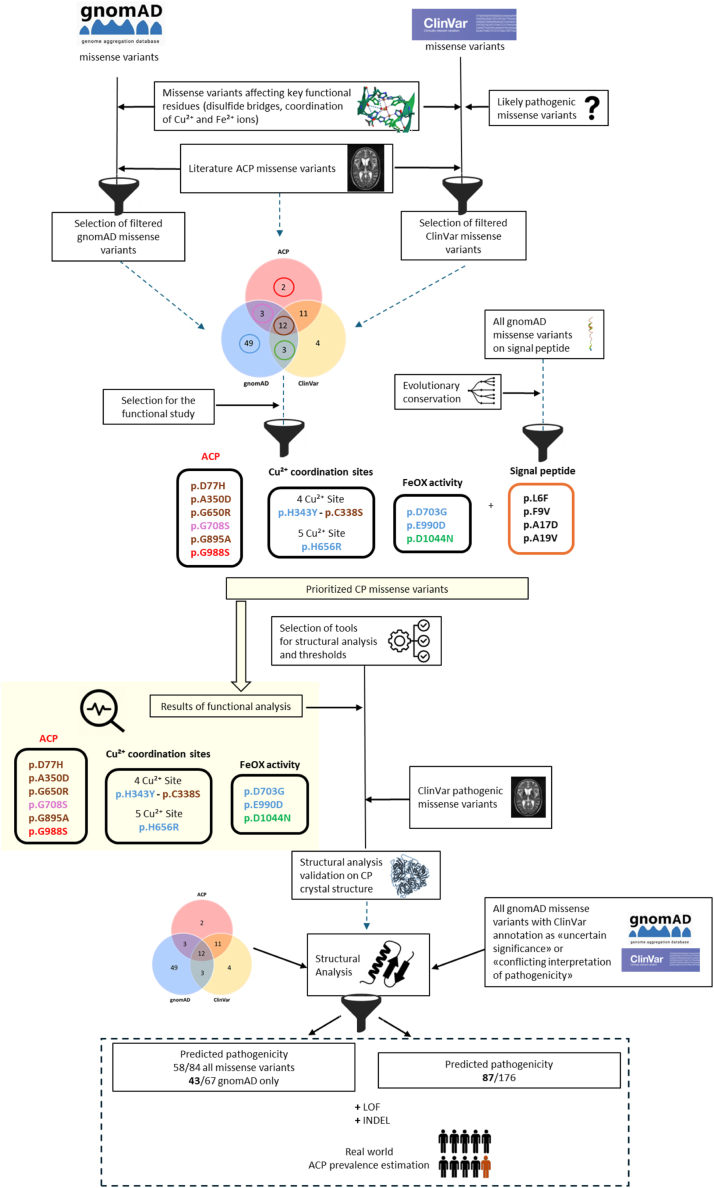
**Schematic representation of the study**.

**Fig. 2:**
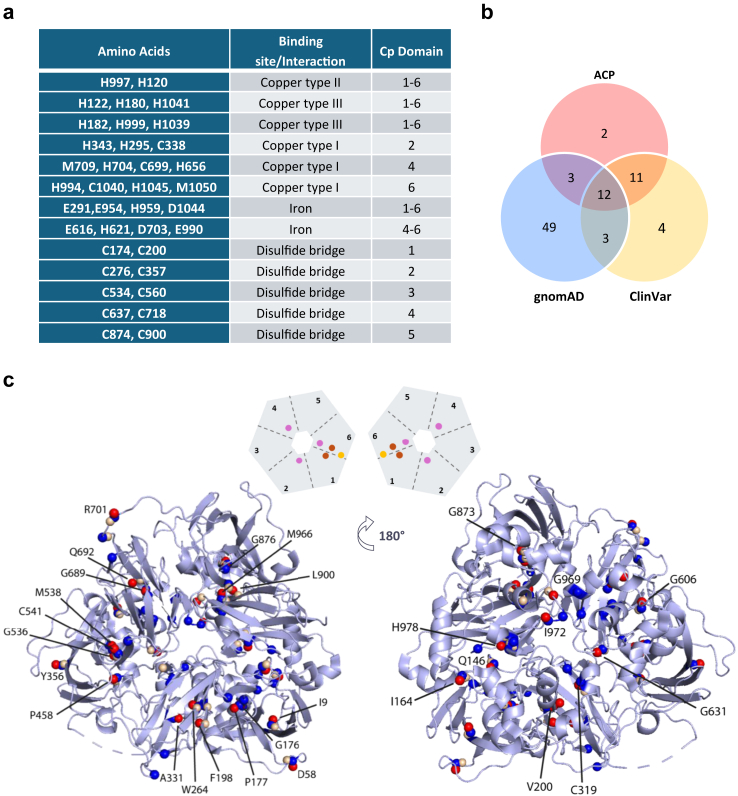
**Functionally important CP residues and new CP missense variants of interest and known ACP missense variants. a.** Table summarising the CP residues key for its structure and function. Data from Uniprot (https://www.uniprot.org/uniprotkb/P00450/entry) and https://doi.org/10.3390/nu5072289. **b.** Venn diagram illustrating the number of identified CP missense variants and their overlaps across the gnomAD (blue) database, ClinVar database (yellow), and ACP literature (red). Missense variants were selected for causing a change in residues involved in disulfide bridges, an interaction with a Cu^2+^ ion, or an Fe^2+^ ion. Variants already associated with ACP, or affecting the same residue but with a different amino acid change are also included. **c.** Top, schematic representation of CP subdivided into the six domains containing Cu^2+^ type I (pink), Cu^2+^ type II (yellow), and Cu^2+^ type III (brown). Bottom, crystal structure of human CP (PDB ID: 4ENZ; view from the top, on the left, and from the bottom, on the right) showing the location of residues of all identified missense variants (blue spheres gnomAD variants; yellow spheres ClinVar variants). Red spheres identify the 28 residues affected by ACP missense variants reported in the literature, are identified based on the mature protein (without signal peptide, 19 amino acids). In some cases (e.g., R701) missense variants are present in all three variant sets (gnomAD, ClinVar and literature ACP sets) and are correspondingly indicated as three separate blue, yellow and red spheres. The figure was generated with the PyMOL program.

**Fig. 3:**
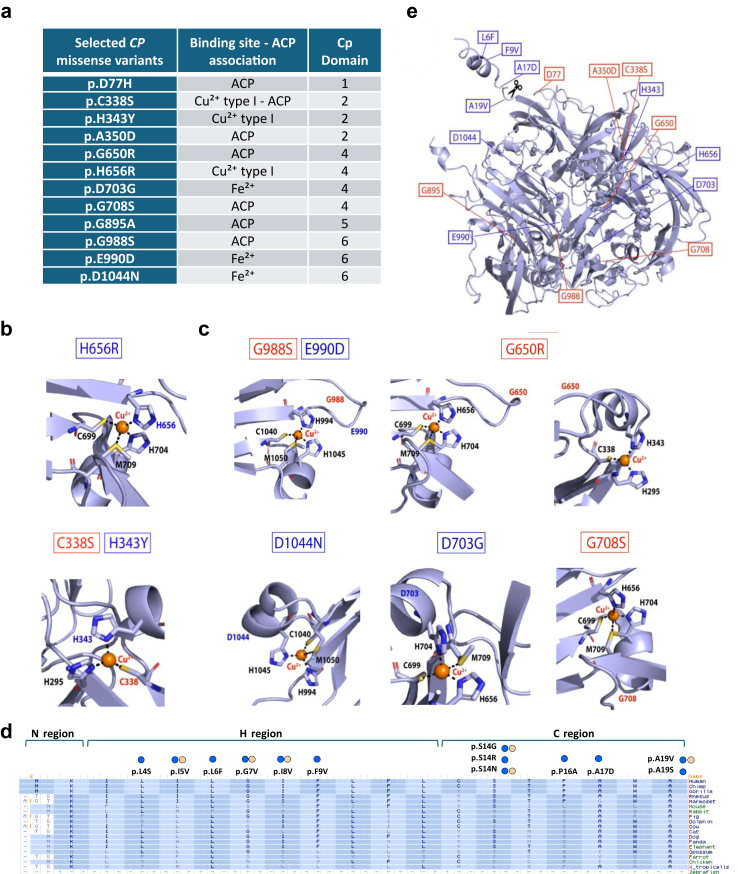
**Selected *CP* missense variants affecting functionally important residues. a.** The 12 missense *CP* variants selected for functional analysis. **b.** Missense variants p.H656R and p.H343Y (see Supplementary Table S2) and ACP mutation p.C338S (see Table 1) which directly impact Cu^2+^ coordinating residues in type I Cu^2+^ centres (1106 and 1102 respectively, using PDB ID: 4ENZ as the reference structure). **c.** Missense variants p.G988S, p.D703G and p.D1044N (see Supplementary Table S2) and ACP mutations p.E990D, p.G708S, p.G650R (see Table 1) which impact residues in close association with type I Cu^2+^ coordinating residues (selected gnomAD missense variants: 1106; missense ACP mutations: 1106 and 1107, respectively), using PDB ID: 4ENZ as the reference structure). p.G650R is in the proximity of two type I Cu^2+^ (1102 and 1106). **d.** Multiple sequence alignment of CP signal peptide using Vertebrate Multiz Alignment & Conservation of UCSC Genome Browser, showing the evolutionary conservation and the missense variants present in gnomAD (**blue** spheres), and ClinVar (**yellow** spheres) databases. The regions of the signal peptide are shown above. Cleavage of the signal peptide occurs after the conserved A-X-A motif. **e.** Alphafold prediction of human CP (structure (https://alphafold.ebi.ac.uk/entry/P00450) showing the location of residues impacted by all selected *CP* missense variants. The cleavage site to generate the mature protein is indicated by scissors.

The structure-function approach towards identifying and prioritizing *CP* missense variants of potential functional significance is illustrated in Fig. 1. CP protein sequence, and key functional amino acids (i.e., residues associated with disulfide bridges, and coordination of Cu^2+^ and Fe^2+^ ions; Fig. 2a) together with known ACP missense variants (Table 1) were retrieved from UniProt (https://www.uniprot.org/uniprotkb/P00450/entry) and the scientific literature, respectively.^1^^,^^8^^,^^18^, ^19^, ^20^, ^21^, ^22^, ^23^, ^24^, ^25^, ^26^, ^27^, ^28^, ^29^, ^30^, ^31^, ^32^, ^33^, ^34^, ^35^, ^36^ All missense variants in the *CP* gene were extracted from gnomAD^37^ and filtered for the CP residues of functional/structural interest (impacting residues associated with disulfide bridges, and coordination of Cu^2+^ and Fe^2+^ ions; Fig. 2b). Missense variants present in ClinVar^38^ were also similarly extracted (Fig. 2b). PyMOL (The PyMOL Molecular Graphics System, Version 3.0 Schrödinger, LLC) was used to visualize the positions of the identified missense variants in the 3D protein crystal structure (4ENZ; Fig. 2c). Candidate uncharacterized pathogenic missense variants for functional characterization were chosen as described in the Results section. With respect to missense variants impacting the CP signal peptide, the evolutionary conservation was inspected with the Vertebrate Multiz Alignment & Conservation in the UCSC Genome Browser (https://genome.ucsc.edu), while the SignalP-6.0 tool (https://services.healthtech.dtu.dk/services/SignalP-6.0/) was used to predict the *in silico* effects of missense variants on the pro-peptide cleavage site^39^ (Fig. 3d).

**Table 1:** List of ACP missense variants reported in the literature.

The list of missense variants associated with ACP was updated on November 2023. Amino acid residue numbering consists of the signal peptide. Missense variants selected for functional study are highlighted in orange. The missense variant p.Thr551Ile Thr551Ile was classified as Benign/likely Bening and was therefore excluded from the analysis.

### Recombinant CP (rCP) mutants production

The rCP-WT plasmid was synthesized from GenScript (Piscataway, NJ, US; RRID:SCR_002891) by cloning the codon-optimized sequence for the human wild-type *CP* (NM_000096.4; includes the signal peptide) into the pcDNA3.1 vector at the *Nhe*I and *Eco*RI sites, followed by a C-terminal TEV protease cleavage site, a 3xC-tag and 3xFLAG tag. To generate different mutants, relevant codon mutations resulting in the desired missense CP mutants were designed and ordered from GenScript (RRID:SCR_002891). All CP constructs were confirmed by sequencing by GenScript Inc. (RRID:SCR_002891). The empty vector (EV) and all plasmids were purchased from GenScript (RRID:SCR_002891).

### Cell culture

HEK293T cells were obtained from American Type Culture Collection (ATCC, #CRL-11268, RRID:CVCL_1926) and grown in heat-inactivated Dulbecco’s modified Eagle’s minimal essential medium (DMEM, Thermo Fisher Scientific, Waltham, MA, US, #41966-029) supplemented with 10% heat inactivated foetal bovine serum (FBS, Thermo Fisher Scientific, #26140079) and 1% PenStrep (Thermo Fisher Scientific, #15140122) at 37 °C in a humidified atmosphere of 95% air and 5% CO_2_. Transient transfection of plasmids encoding EV, rCP-WT, and rCP mutants into HEK293T cells was performed using Lipofectamine 2000 (Thermo Fisher Scientific, #11668-019) and Opti-Mem (Thermo Fisher Scientific, #31985062) according to the manufacturer’s instructions. Media were supplemented with 10 μM CuSO_4_·5H_2_O 5 h and 21 h post-transfection, with the exception of the WT negative control. 48 h after transfection, 4 mL of each collected cell supernatant were concentrated and buffer-changed to Tris buffer (20 mM, pH 7.0) using Amicon-Ultra-4 30 kDa devices (Merck, Darmstadt, DE, #UFC8030). Transfected cells were lysed with M-PER™ Mammalian Protein Extraction Reagent (Thermo Fisher Scientific, #78501) supplemented with a Halt Protease Inhibitor Cocktail (Thermo Fisher Scientific, #87786). The total protein concentration in each cell lysate or supernatant was determined using the Bradford assay (Thermo Fisher Scientific, #23236).

### Turbidimetry

Antigen levels of CP in cell lysates and supernatants were determined using an Optilite turbidimeter (Binding Site, Birmingham, UK) and the Ceruloplasmin kit (Binding Site, #NK045.OPT) for diagnostic purposes in clinical settings. Tests were performed in triplicate according to manufacturer’s instructions and using automated dilutions. The percentage of intra- and extracellular CP for each mutant was calculated based on the total antigen derived from the CP concentrations in the lysate and supernatant, and normalized to the sample volumes.

### CP enzymatic activity assays

Total CP activity was measured in 96-well plates using the Ceruloplasmin Colorimetric Activity Kit (Thermo Fisher Scientific, #EIACPLC) following manufacturer’s instructions. The activity of the standard in the kit was determined using the Curzon and Vallet method under conditions of oxidase activity determination.^40^ Briefly, concentrated and dialysed samples were diluted in assay buffer, added to the substrate, incubated at 37 °C for 1 h, and then the plates were read at 560 nm. Ferroxidase (FeOx) CP activity of concentrated and dialysed samples was measured according to Erel’s method^41^ doubling the reaction volumes optimal for cuvettes. Briefly, 6 μL of sample was added to 700 μL of reagent 1. Then, 150 μL of reagent 2 was added to the mixture, and after incubation for 30 min at 37 °C, the reaction was stopped by adding 60 μL of reagent 3. The absorbance was measured at 600 nm with a correction at 700 nm using a Nanodrop One spectrophotometer (Thermo Fisher Scientific). Each sample was assayed thrice. The calibration was based on 7-point EDTA concentrations, in which the ferrous (Fe^2+^) ion concentration was derived.

### Immunoblots

Equal amounts of total protein or CP from cell lysates or supernatants were resolved using TGX stain-free 4–15% (BioRad, Hercules, CA, US; #5678085) gels or 4–12% Bis-Tris gels (Thermo Fisher Scientific, #WG1403) along with plasma-derived CP standards: human plasma-derived CP Kedrion (pdCP-K),^16^ commercial human plasma-derived CP research-grade purified (pdCP-E) (Enzo Life Sciences, #ALX-200-089-M001), and a standard from the CP Colorimetric Activity Kit (Thermo Fisher Scientific, #EIACPLC). The proteins were transferred to nitrocellulose membranes (Bio-Rad, #1704159) using the Trans-Blot Turbo Transfer System (Bio-Rad). The membranes were then blocked with EveryBlot Blocking Buffer (Bio-Rad, #12010020) and stained overnight at 4 °C using one of the following primary antibodies: anti-serum CP kit Optilite (1:2000, Binding Site, #NK045.OPT), CP monoclonal antibody (1:1000, Thermo Fisher Scientific, #MA5-38035, RRID:AB_2897953), CP polyclonal antibody (1:1000, Thermo Fisher Scientific, #PA5-95336, RRID:AB_2807139), polyclonal anti-Cp (1:1000, Abcam, Cambridge, UK; #ab48614, RRID:AB_869113). Membranes were incubated with appropriate secondary antibodies at room temperature for 1 h: rabbit anti-sheep HRP (1:2000, Thermo Fisher Scientific, #31480, RRID:AB_228457) and donkey anti-rabbit HRP (1:2000, Cytiva, Milan, IT; #NA9340, RRID:AB_772191). The membranes were developed using Clarity Western ECL Substrate (Bio-Rad, #1705061) and exposed to ChemiDoc (Bio-Rad).

### Immunocytochemistry

For immunofluorescence analysis, 50,000 cells/well were seeded on poly L-lysine-coated coverslip glasses (13 mm Ø × 0.17 mm) and transfected with pcDNA3.1 plasmid containing rCP-WT or CP mutants (p.A17D, p.A19V, p.D77H, p.A350D, and p.G895A) using Lipofectamine LTX (Thermo Fisher Scientific, #15338100). After 5 and 24 h, the medium was supplemented with 10 μM CuSO_4_·5H_2_O. 48 h post-transfection, cells were fixed using Image-IT Paraformaldehyde 4% (Thermo Fisher Scientific, #I28800) and permeabilized with PBS containing 0.25% Triton X-100 for 20 min. The cells were then blocked for 2 h in PBS supplemented with 5% BSA and 0.1% Triton X-100. For double staining, the cells were incubated for 16 h at 4 °C with antibodies diluted in PBS containing 2.5% BSA and 0.1% Triton X-100. The antibodies used were rabbit anti-Cp (1:100, BioVision, #7019, RRID: AB_3665653), rat anti-GRP94 (1:100, Enzo Life Sciences, #ADI-SPA-850-D, RRID:AB_2039133), and mouse anti-GM130 (1:100, BD Biosciences, #610822, RRID:AB_398141). After washing with PBS, the cells were incubated for 1 h with goat anti-rabbit Alexa Fluor-594 (1:500, Thermo Fisher Scientific, #A32740, RRID:AB_2762824), goat anti-rat Alexa Fluor-488 (1:500, Thermo Fisher Scientific, #A11006, RRID:AB_2534074), or goat anti-mouse Alexa Fluor-488 (1:500, Thermo Fisher Scientific, #A11029, RRID:AB_2534088). Cells were then incubated for 5 min with 300 nM DAPI (4',6-diamidino-2-phenylindole, ThermoFisher Scientific, #D1306) in PBS. After washing four times with PBS and once with ultrapure water, coverslips were mounted using ProLong Diamond Antifade Mountant (Thermo Fisher Scientific, #P36934) and sealed with ProLong Coverslip Sealant (Thermo Fisher Scientific, #P56128). Staining was visualized using a Leica TCS SP5 Laser Scanning Confocal Microscope equipped with a 63× objective, and images were acquired using LAS-AF software (Leica). Images were reconstructed by stacking five consecutive z scans (z step 0.9 μm) with ImageJ software.

### *In silico* structure-based mutational analysis

Structural analysis of the CP mutants to predict their impact on protein folding in terms of free energy change (ΔΔG) was performed using the PremPS,^42^ DDMut,^43^ DUET,^44^ DynaMut2,^45^ and PoPMuSiC^46^ servers using the CP crystal structure (Protein Data Bank: 4ENZ) and AlphaFold.^47^ Missense variants predicted to be destabilized by three of these five tools were selected to estimate the prevalence of ACP.

### Prevalence estimates

Carrier frequencies and prevalence estimates were calculated with the Genetic Prevalence Estimator (GeniE, https://genie.broadinstitute.org/). This tool employs gnomAD allele frequencies to assess the genetic prevalence of autosomal recessive diseases. We selected the GeniE simplified model, which is based on the Hardy–Weinberg equation for the calculation of genetic prevalence. We opted for the GeniE manual function, by submitting 3 custom lists of variants: 1) one based on a rational selection of variants (including LoFs, start/stop codon losses, in-frame deletion/insertions, as well as missense substitutions identified through our functional/structural analyses); 2) the second one, more unbiased, based on the following criteria: i) minor allele frequency ≤0.01, and ii) inclusion of all LoFs (comprising also 4 structural variants/large deletions), start/stop codon losses, in-frame deletion/insertions, as well as missense substitutions with a predicted REVEL score ≥0.932 (strong missense variants); and 3) the third list mirrored the second one, but included missense substitutions with a predicted REVEL score of ≥0.773 (moderate missense variants). REVEL (rare exome variant ensemble learner) is a specific method developed to predict and classify pathogenicity of missense variants based on 13 individual tools. The REVEL score threshold to assign missense variants as having “strong” (≥0.932) or “moderate” (≥0.773) evidence of variant pathogenicity was chosen in agreement with ClinGen recommendation.^48^ In all cases, variants were retrieved from gnomAD v.4.1.0 and gnomAD SV v.4.1.0 (for structural LoF variants), and the Matched Annotation from NCBI and EMBL-EBI (MANE) transcript ENST00000264613.11/NM_000096.4 of the *CP* gene for variant reporting.

### Statistics

Statistical calculations and data visualization were performed using GraphPad Prism v.10 (GraphPad Software, Inc. La Jolla, CA, USA). Normality of data distribution (levels of antigen, and specific activities) was assessed using the Shapiro–Wilk test. Accordingly, the statistical data analysis was performed using one-way analysis of variance (ANOVA) followed by correction for multiple comparisons using Dunnett’s method (∗P ≤ 0.05, ∗∗P < 0.01, ∗∗∗P < 0.001, ∗∗∗∗P < 0.0001 versus WT rCp).

### Role of funders

The funder Kedrion S.p.A. had a direct role in study design, data collection, data analyses, interpretation, and writing of report. The funder Italian Ministry of Universities and Research (MUR) had no role in the study design, data collection, analyses, interpretation, or writing of the report.

## Results

### Identification of *CP* missense variants with relevance for pathogenicity

While variants resulting in a truncated or missing protein can be easily associated with a LoF phenotype, missense substitutions may or may not result in impaired protein function and require functional characterization. The pathogenic potential of missense substitutions can be inferred from their association with residues of functional importance in the protein. In CP, structure and function are dependent on the appropriate coordination of Cu^2+^ ions for correct protein folding and Fe^2+^ ions for ferroxidase activity. According to the literature, six Cu^2+^ ions are present in holoceruloplasmin (Holo-CP; distinct from the Cu-uncomplexed form defined as apoceruloplasmin, Apo-CP), subdivided into three spectroscopically distinct forms (type I, II, and III).^49^ Among these, three Cu^2+^ ions (two of type III and one of type II), which form the trinuclear cluster, have been identified as being responsible for the process of electron transfer from the substrate to be oxidised to molecular oxygen, and thus for the enzymatic activity of CP.^50^^,^^51^ Therefore, it is possible to identify key CP amino acid residues that are associated with correct folding (including residues associated with disulfide bridges) and functional properties based on the coordination of Cu^2+^ and Fe^2+^ ions. Aside from missense substitutions affecting these known functionally relevant residues, a number of ACP-associated pathogenic missense variants are known. CP residues key for its structure and function were identified (n = 37; Fig. 2a), as well as missense pathogenic variants in *CP* associated with ACP (n = 28; Table 1). All missense substitutions in *CP* reported in the gnomAD database were then extracted, resulting in the identification of 807 individual impacted residues out of 1065 amino acids (including the signal peptide) comprising the soluble form of CP. Next, gnomAD missense *CP* variants coincident with residues associated with functionally important amino acids (disulfide bridges and interactions with Cu^2+^ or Fe^2+^ ions) or resulting in known ACP pathogenic variants were selected. This analysis resulted in a subset of 67 gnomAD *CP* missense variants (Supplementary Table S1), all of which were present in the database as heterozygous variants, with the exception of two reportedly pathogenic variants previously associated with ACP (p.P477L and p.G895A). Similar to the screening performed on gnomAD *CP* missense variants, *CP* missense variants present in the ClinVar database were filtered for amino acid substitutions affecting functionally important residues, or those resulting in ACP (n = 29). In addition to the identified 29 *CP* missense variants, we included the p.R310G variant based on its classification as “likely pathogenic” in ClinVar (Supplementary Table S1). Collectively, the pool of potentially pathogenic variants numbered 84 (Fig. 2; Supplementary Table S1).

### Functional characterization of selected CP missense variants

Of these 84 variants, 12 missense *CP* variants were selected for functional analysis (Fig. 3a). The trinuclear cluster comprising type II and III Cu^2+^ ions is located at the C-terminus of the linear CP sequence.^5^^,^^49^, ^50^, ^51^ This cluster comprises 8 histidine residues (H120, H122, H180, H182, H997, H999, H1039, H1041; Supplementary Fig. S1). Amongst these histidine residues H997 and H999 are impacted by two gnomAD variants (p.H999Q and p.H997R, Supplementary Fig. S1 and Table S1). Additionally, H180 is impacted by a known ACP mutation (p.H180N, Supplementary Fig. S1 and Table S1). These missense variants have not been prioritized for functional analysis as their disruption of key Cu^2+^ coordinating histidine residues in the trinuclear cluster likely results in incorrect CP folding and impaired enzymatic activity (though no Clinvar annotation is as yet available). Instead, the focus was placed on *CP* missense variants impacting residues associated with Cu^2+^ type I ion coordination, which are spread over the entire length of the linear CP sequence and are so far believed to be less important for CP folding than type II and III Cu^2+^ ions.^5^ The 12 selected missense *CP* variants included seven missense variants previously associated with ACP (p.D77H, p.C338S, p.A350D, p.G650R, p.G708S, p.G895A, and p.G988S) as pathogenic (positive) controls. All missense variants, with the exceptions of p.G988S and p.G708S, were identified in both gnomAD and ClinVar databases (Table 1). Variants p.C338, p.A350, p.G650, p.G708, and p.G988 were chosen due to their direct or indirect involvement in Cu^2+^ type I ion coordination (Fig. 3b and c). In contrast, p.D77H and p.G895A, while associated with ACP, do not appear to influence Cu^2+^ or Fe^2+^ coordination. As uncharacterized candidate pathogenic ACP missense variants, we selected H343Y, H656R, R703G, E990D, and D1044N because they directly impact residues coordinating Cu^2+^ type I or Fe^2+^ ions.

Finally, since variants in the signal peptide may impact protein secretion, we extracted all missense variants present in the gnomAD and ClinVar databases that map within the CP signal peptide (Fig. 3d). Their effect on CP protein secretion was predicted with the SignalP-6.0 server (Fig. 3d).^39^^,^^52^^,^^53^ This analysis identified p.A17D and p.A19V as affecting signal peptide cleavage, and these *CP* missense variants were therefore selected for functional characterization. Two additional missense variants in the CP signal peptide (p.L6F and p.F9V) were selected for functional analysis based on evolutionary conservation of the impacted residues among vertebrates and the charge effect/steric hindrance of the amino-acid substitution (Fig. 3d). Collectively, the selected CP variants (Fig. 3e) were analysed by transient expression in the human cell line HEK293T to determine their effects on CP secretion, ability to form Holo-CP, and enzymatic activity of the secreted CP protein.

### Analysis of intracellular retention of CP variants

**Fig. 4:**
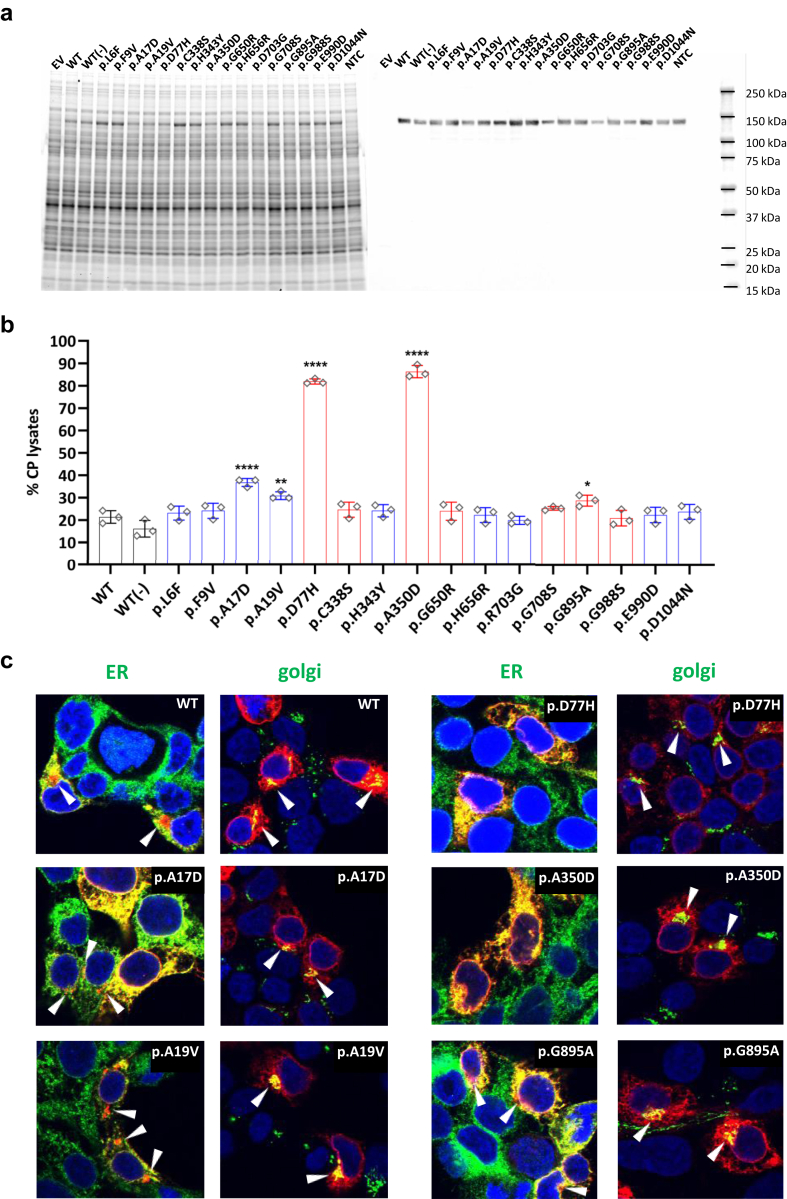
**Intracellular retention of rCP mutants. a.** Coomassie staining (left) and anti-CP immunoblot (right) of cell lysates from HEK293T cells transfected with either empty vector (EV), WT rCp or the indicated mutants. Media were supplemented with CuSO_4_·5H_2_O with the exception of WT (−) rCP (negative control). Cell lysate from non-transfected cells (NTC) was also included as control. Cell Lysates were subjected to denaturing and reducing SDS-PAGE on TGX stain-free 4–15% gel. 10 μg of total protein (shown on the left) were loaded in each well and blotted with anti-CP antibodies (shown on the right). **b.** 48 h post-transfection cell lysates and supernatants were collected, and CP antigen levels were determined by turbidimeter assay. The percentage of intra- and extracellular CP for each mutant was calculated on the total antigen derived from the CP concentration in the lysates and supernatants normalized for sample volumes. Bars represent the mean ± the standard deviation from three independent experiments. Statistical analysis was performed using ANOVA followed by correction for multiple comparisons using Dunnett’s method (∗P ≤ 0.05, ∗∗P < 0.01, ∗∗∗P < 0.001, ∗∗∗∗P < 0.0001 versus WT rCp). Black lines indicate controls, **blue** lines are the new *CP* candidate pathogenic variants, and **red** lines are the ACP literature variants. **c.** Representative images for the staining of the CP (red) and endoplasmic reticulum (ER; anti-GRP94, green) or golgi (anti-GM130, green) in HEK293T cells. Cell nuclei are counterstained using DAPI (blue).

Most previous *in-vitro* studies have focused on the glycosyl-phosphatidylinositol (GPI)-membrane anchored CP form^18^, ^54^, ^55^ produced physiologically by alternative splicing.^3^ This is not the predominant soluble form circulating in plasma and thus is not fully representative for studying ACP. Therefore, we transiently expressed the soluble form of WT rCP and the selected CP variants in the human HEK293T cell line, resulting in high expression levels (Fig. 4a). Antigen levels of CP in cell lysates and supernatants were measured by a turbidimetric assay routinely used for ACP diagnostic purposes in clinical settings.^54^ Since we had evidence that absorbance resulting from immune complexes decreased significantly in turbidimetry after boiling CP, we hypothesized that the assay was affected by CP protein conformation, resulting in underestimation of CP protein levels when the studied variants impacted protein structure. To avoid confounding effects due to variants altering immunocomplex formation, the percentage of intra- and extracellular CP for each individual mutant was calculated based on total antigen levels. As shown in Fig. 4b, the secretion of ACP mutants p.D77H and p.A350D was significantly impaired (∼80%) in spite of clearly detectable intracellular expression, consistent with literature data showing that these variants result in retention within the Endoplasmic Reticulum (ER).^1^^,^^56^ Variants in the signal peptide (p.A17D and p.A19V) significantly reduced the secretion of rCP compared to the wild-type protein (Fig. 4b), suggesting that these signal peptide variants affect CP secretion and may result in decreased circulating CP levels *in vivo*. A limited but statistically significant effect on secretion was observed for the p.G895A variant (Fig. 4b). The observed impaired CP secretion for p.A17D, p.A19V, p.D77H, p.A350D, p.G895A could be due to impaired endoplasmic reticulum (ER)-to-Golgi trafficking as previously reported in literature for some of them.^18^^,^^56^ To gain insight into the intracellular trafficking of mutants with reduced secretion, we investigated their subcellular localization using immunofluorescence and confocal microscopy analysis, using subcellular compartment markers for the ER and Golgi. Cells transfected with WT rCP showed CP co-localization with the ER, with a typical pattern of vesicular haze extensively dispersed throughout the cytosol, and a punctate CP signal consistent with its colocalization in the Golgi apparatus, as demonstrated by colocalization of the CP signal with a Golgi marker (Fig. 4c). A similar staining pattern was observed in cells transfected with the p.A17D, p.A19V, and p.G895A rCP mutants, with no apparent differences relative to cells transfected with WT rCP. In contrast, in cells transfected with the p.D77H and p.A350D mutants, a less punctuated and more evenly spread rCP signal was observed, with qualitatively higher ER co-localization and little Golgi co-localization detectable, consistent with an impairment in p.D77H and p.A350D mutant maturation due to defective ER-to-Golgi trafficking (Fig. 4c). Therefore, ER retention was observed for the D77H and A350D mutants, as previously reported,^56^ which was consistent with the strong effects of these variants on CP secretion (Fig. 4b). In contrast, the effects of p.A17D, p.A19V, and p.G895A on the impairment of ER-Golgi trafficking were not as obvious, somewhat consistent with a relatively more subtle effect of these variants on CP secretion (Fig. 4b).

### Analysis of Holo-form generation and enzymatic competence of secreted CP variants

**Fig. 5:**
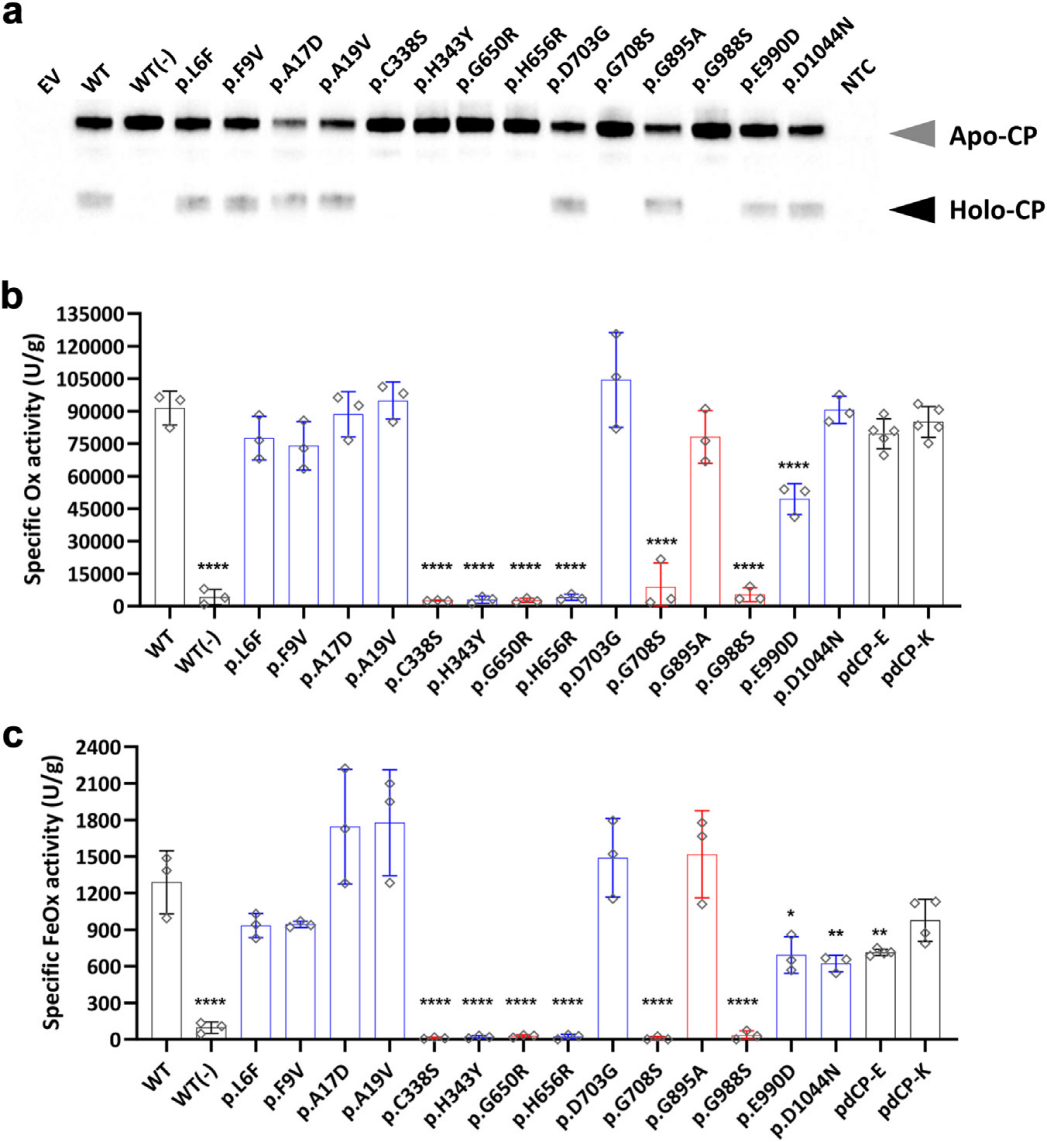
**Functional characterization of rCP mutants and plasma derived CPs. a.** Immunoblots of cell culture supernatants from HEK293T cells transfected with either empty vector (EV), WT rCp or the indicated mutants. Media were supplemented with CuSO_4_·5H_2_O with the exception of WT (−) rCP (negative control). Medium from non-transfected cells (NTC) was also included as control. 48 h post-transfection, supernatants were subjected to partially denaturating and non-reducing SDS-PAGE on 4–12% Bis-Tris gels to reveal the presence of Apoceruloplasmin (Apo-CP) and Holoceruloplasmin (Holo-CP), indicated by grey and black arrowheads, respectively. Cell culture supernatants containing 10 ng of CP were loaded in each well, except for EV and NTC for which 3 μg of cell culture supernatant total protein were loaded. **b.** Specific Oxidase (Ox) and **c.** specific Ferroxidase (FeOx) CP activities of concentrated and dialysed cell culture supernatants. For each analytical session, the basal activity of EV was subtracted from the activity values of rCP mutants of the respective transfections. Activities were also measured for human plasma-derived CP Kedrion (pdCP-K) and a commercial research-grade purified CP (pdCP-E), included as controls. Specific activity was calculated by normalizing Ox and FeOx activities in cell culture supernatants on their corresponding CP antigen levels. Bars (**b,c**) represent the mean ± s.d. from three independent experiments. **Black** lines indicate controls, **blue** lines are the new candidate pathogenic variants, and **red** lines are the ACP literature variants. The statistical data analysis was performed using one-way analysis of variance (ANOVA) followed by correction for multiple comparisons using Dunnett’s method (∗P ≤ 0.05, ∗∗P < 0.01, ∗∗∗P < 0.001, ∗∗∗∗P < 0.0001 versus WT rCp).

Next, the rCP missense variants for which efficient secretion from HEK293T cells in the culture medium could be demonstrated were investigated through the analysis of the corresponding conditioned media for their oxidase (Ox) and ferroxidase (FeOx) activities, thus excluding the p.D77H and p.A350D mutants from further analysis. Both Ox and FeOx assays are commonly employed for the characterization of CP activity, with the FeOx assay being the principal functional assay to measure CP activity in human plasma samples.^8^^,^^57^^,^^58^ Because FeOx activity represents an iron-dependent component of oxidase activity, we decided to employ both assays to assess any potential functional effects of individual missense variants on the capacity of CP to oxidize different substrates. In each assay and for each rCP protein conditioned media, CP activity was measured and normalized to CP antigen levels to produce specific activity values and compared to plasma-derived purified CP proteins as positive controls, namely from Kedrion (pdCP-K^16^) and from a commercial source (pdCP-E^15^^,^^16^). As a negative control, WT rCP was expressed in the absence of Cu^2+^ supplementation in the medium (−), which is known to result in an Apo-CP protein devoid of functional activity.^59^ In fact, WT rCP expressed in HEK293T cells in the absence of Cu^2+^ supplementation resulted in the secretion of Apo-CP (Fig. 5a), completely devoid of either Ox or FeOx activity (Fig. 5b and c). However, WT rCP expressed in the presence of supplemented Cu^2+^ resulted in the secretion of significant quantities of Holo-CP (Fig. 5a) with an Ox activity that was at least comparable to that of plasma-derived CP samples (Fig. 5b). Among the rCP mutants expressing an ACP-associated variant, loss of Holo-CP (Fig. 5a) and loss of both Ox and FeOx activity was confirmed for p.C338S, p.G650R, p.G708S, and p.G988S (Fig. 5b and c). However, p.G895A showed a profile of Holo-CP and Ox/FeOx activities comparable to that of the WT rCP protein, inconsistent with a pathological significance of this variant, as some studies have also suggested.^54^^,^^56^ Of the nine yet unexplored rCP variants, the p.H343Y and p.H656R variants, which alter residues directly interacting with copper type I ions (Fig. 3c) demonstrated loss of Holo-CP expression (Fig. 5a) and almost null specific Ox and FeOx activities (Fig. 5b and c). Conversely, the p.D703G variant did not result in impaired Holo-CP formation or Ox/FeOx activity (Fig. 5a,b,c). The p.D1044N and p.E990D variants did not result in impaired Holo-CP formation (Fig. 5a) but demonstrated intriguing differences in their enzymatic activities relative to WT rCP. Specifically, p.E990D demonstrated a partial reduction (∼40%) in both Ox and FeOx activities with respect to WT rCP (Fig. 5b), but showed a specific FeOx activity (Fig. 5c) comparable to that of plasma-derived CP proteins (demonstrated to be efficacious in Cp-KO mice^15^^,^^16^). As for p.D1044N, only its FeOx activity was partially impaired (approx. 40%; Fig. 5b and c). Finally, the variants affecting the signal peptide, which may result in the secretion of a CP with a different amino-terminus relative to the WT rCP, did not affect Holo-CP formation (Fig. 5a), as might be expected from variants that do not impact CP residues key for Cu^2+^ loading and protein folding, and had no significant impact on protein FeOx and Ox activities (Fig. 4b and c). Therefore, none of the signal peptide missense CP variants could be considered to have pathological significance.

### Structural analyses and rational prediction of pathogenicity for gnomAD *CP* missense variants

An estimate of the real-world prevalence of ACP can be obtained through the analysis of pathogenic and potentially pathogenic *CP* variants described in the human population. To this end, an *in-silico* workflow was applied to identify CP variants with pathogenic potential, intended as variants capable of altering the protein structure locally or globally (i.e., in terms of experimental free energy change, ΔΔG_exp_). First, as a validation set for the workflow, the 12 missense mature CP variants that underwent functional characterization (Fig. 3a) were selected. Next, PremPS, DDMut, DUET, and DynaMut2 were employed to examine the destabilizing potential of each of these 12 *CP* variants using either the CP crystal structure (PDB ID:4ENZ) or the CP AlphaFold-predicted structure (P00450). As the results were comparable (Supplementary Table S2), the CP crystal structure was used, which allowed the inclusion of a fifth tool, PoPMuSiC. Then, the 12 selected missense variants together with the known pathogenic variants on ClinVar (n = 8) were used to validate the structural analysis using the threshold of ΔΔG_exp_≥1.0 kcal mol^−1^ for highly destabilizing variants for PremPS and PoPMuSiC (as previously reported^42^), and the widely used ΔΔG_exp_≥0.5 kcal mol^−1^ for DDMut, DUET and DynaMut2. Missense variants predicted as destabilizing by at least three of these five tools showed a true-positive rate of 81% and a true-negative rate of 100% (Supplementary Table S2). With these parameters, we performed an analysis of the 84 missense variants (Fig. 2b; gnomAD subset). Of these, 58 (69%) were predicted to have destabilizing (pathogenic) effects (Supplementary Table S3). Then, among all gnomAD missense variants, we extracted 176 variants annotated in the ClinVar database as of “uncertain significance” or “conflicting interpretations of pathogenicity: 87 of them were predicted to be destabilizing on the CP structure (Supplementary Table S3). Structural analysis was not performed for two variants, p.S501R and p.T1062N, because they fall into regions of CP which are not represented in the PDB structure. A total of 244 missense variants from gnomAD were included in the analysis, of which 130 were predicted to have pathogenic effects (53%) (Supplementary Table S3). These rationally selected 130 missense CP variants present in gnomAD were therefore included for ACP prevalence estimations.

### Assessment of ACP prevalence from gnomAD

Prevalence estimates for ACP were calculated using the recently released Genetic Prevalence Estimator tool (GeniE, https://genie.broadinstitute.org), which leverages general population allele frequencies present in gnomAD. Only variants included in the more recent gnomAD v4.1.0 and passing quality checks were used. A total of 324 variants were submitted to GeniE: i) 125 predicted pathogenic missense variants and the p.D77H and p.H343Y were included based on our functional/structural analyses; ii) 177 LoFs (frameshift, nonsense, and splicing variants affecting the first two and the last two intronic nucleotides); iii) 3 start/stop codon loss variants; and iv) 17 in-frame deletions/insertions. The estimated global prevalence of ACP was 12.6/10^6^ individuals (Table 2), ∼20–25 fold higher than that reported to date.^9^^,^^10^ Similarly, the prevalence rate in the European (non-Finnish) population was 12.9/10^6^ individuals, while the prevalence in the East Asian population was 57.4/10^6^ individuals, a much higher prevalence than in the other populations (Table 2). The recurrent and ethnicity-specific variants are summarized in Table 2.

**Table 2:** GeniE estimated prevalence of Aceruloplasminemia and most frequent mutations.

Genetic ancestry group	Estimated heterozygous frequency in 10^3^ individuals	Estimated prevalence in 10^5^ individuals	Top 5 most frequent mutations	Structural analysis
Global	7.09	1.265^b^	p.N263S	5/5
			p.A406E	3/5
			p.I453V	5/5
			p.G1000S	5/5
			p.L592F	3/5
African/African-American	5.24	0.689	p.I894T	5/5
			p.E803G	3/5
			p.G967A	4/5
			p.R177K	5/5
			p.R89TfsTer14	–
Latino/Admixed American	8.58	1.855	p.M1?	–
			p.R310H	3/5
			p.K422R	4/5
			p.L634I	3/5
			p.N263S	5/5
Ashkenazi Jewish	1.31	0.043	c.∗13+1G > A (splice donor variant)	–
			c.1348+2 T > C (splice donor variant)	–
			p.T491C	5/5
			p.N1059MfsTer9	–
			p.E803del	–
East Asian	15.0	5.740	p.M1?	–
			p.H130Q	4/5
			p.L592F	3/5
			p.G1000S	5/5
			p.W55CfsTer8	–
European (Finnish)	2.68	0.180	p.W205Ter	–
			p.N263S	5/5
			p.H621P	3/5
			p.Y67H	5/5
			p.T841RfsTer52	–
Middle Eastern	9.03	2.057	p.N263S	5/5
			p.G1000S	5/5
			p.S833Ter	–
			p.R785Q	5/5
			p.M288T	5/5
European (non-Finnish)	7.17	1.294	p.N263S	5/5
			p.A406E	3/5
			p.I453V	5/5
			c.∗13+1G > A (splice donor variant)	–
			p.L592F	5/5
Remaining	7.48	1.408	p.A406E	3/5
			p.N263S	5/5
			c.∗13+1G > A (splice donor variant)	–
			p.I453V	5/5
			p.Y67H	5/5
South Asian	8.24	1.711	p.G1000S	5/5
			p.M288T	5/5
			p.D77H	2/5^a^
			p.A378T	4/5
			p.I736N	4/5

The consequences on native protein numbering are reported.

a Missense variant functionally characterized.

b 95% Confidence intervals for the global population only are (1.241–1.289).

To provide a broader perspective, we calculated additional prevalence estimates using a less selective approach. All gnomAD CP variants predicted to be pathogenic with a minor allele frequency of ≤0.01 were included, consistent with common methodologies for rare disease prevalence estimation.^60^, ^61^, ^62^ We generated two datasets applying different thresholds for missense variant inclusion. The first dataset employed a stringent criterion, considering only “strong” damaging missense variants, while the second incorporated both “strong” and “moderate” missense substitutions.^48^ Of note, both datasets also included structural LoF variants (i.e., large deletions, n = 4) identified from genome data (gnomAD SV v.4.1.0, based on information from 63,046 individuals). Hence, the two datasets comprised 248 and 546 variants, respectively (Supplementary Table S4). Our analysis yielded overall prevalence estimates ranging from 6.64/10^6^ to 21.7/10^6^ individuals. This is in line with the number of homozygous individuals annotated in the two datasets (12 and 4 among 807,162 individuals for the less and more stringent dataset, respectively). Table 3 provides a breakdown of prevalence estimates across different ethnic groups. Interestingly, prevalence estimates are profoundly affected by a single missense variant, i.e., the p.G895A substitution (rs139633388), which is the most frequent one among those included in our analyses (global minor allele frequency: 1.82/10^3^). The global prevalence of ACP is strikingly decreasing from 8.01 to 0.57/10^6^ individuals depending on the inclusion of the p.G895A variant (most stringent dataset; Table 3). This underlines the importance of a functional assessment of predicted pathogenic variants, like p.G895A, as presented in this work (in the ClinVar database, p.G895A is indeed reported with conflicting classifications of pathogenicity).

**Table 3:** GeniE estimated prevalence of Aceruloplasminemia based on predicted pathogenic gnomAD variants.

	Most stringent threshold set (LoF + Strong Missense^a^) n = 248		Less stringent threshold set (LoF + Moderate Missense^b^) n = 546	
Genetic ancestry group	Estimated heterozygous frequency in 10^3^ individuals (c)	Estimated prevalence in 10^5^ individuals (c)	Estimated heterozygous frequency in 10^3^ individuals (c)	Estimated prevalence in 10^5^ individuals (c)
Global	5.14 (1.51)	0.66 (0.057)^d^	9.27 (5.65)	2.17 (0.80)^d^
African/African-American	1.82 (1.23)	0.083 (0.038)	21.2 (20.6)	11.43 (10.81)
Latino/Admixed American	7.98 (2.40)	1.60 (0.14)	12.8 (7.21)	4.12 (1.31)
Ashkenazi Jewish	2.97 (0.47)	0.22 (0.006)	4.38 (1.89)	0.48 (0.09)
East Asian	3.57 (3.52)	0.32 (0.31)	9.28 (9.24)	2.17 (2.15)
European (Finnish)	3.80 (1.43)	0.36 (0.051)	5.55 (3.17)	0.77 (0.25)
Middle Eastern	1.70 (1.05)	0.073 (0.027)	8.60 (7.94)	1.86 (1.59)
European (non-Finnish)	5.76 (1.50)	0.83 (0.057)	8.56 (4.32)	1.85 (0.47)
Remaining	4.58 (0.99)	0.53 (0.025)	9.31 (5.75)	2.19 (0.83)
South Asian	1.52 (1.37)	0.058 (0.047)	10.9 (10.8)	3.03 (2.95)

Only predicted pathogenic variants with minor allele frequency ≤0.01 were included.

a REVEL score ≥0.932.

b REVEL score ≥0.773.

c In parentheses we reported the calculated estimates after removing the p.G895A variant.

d 95% Confidence intervals for the global population only are [0.646–0.682] (Most stringent threshold set) and [2.14–2.20] (Less stringent threshold set).

## Discussion

In this study, a rational approach is presented that integrates aspects of CP genetics, genomics and functional characterization to validate *in-silico* structural analyses and predict mutant pathogenicity, with the ultimate goal of providing an approximation of ACP prevalence (intended as *birth prevalence* throughout this manuscript) in a real-world data population. This analysis, covering a set of candidate pathogenic CP variants as well as known ACP mutations, demonstrated that missense CP variants can impact protein function without a major impact on protein levels. This is highly relevant for ACP diagnosis as CP measurement in patients is often based solely on CP protein levels.^57^ Valuable insights into the role of key CP functional residues were obtained in the process, and the information gathered was used to develop a workflow enabling the rational bioinformatics prioritization of potentially pathogenic CP missense variants present in gnomAD. Together with structural, LoF CP variants, these potentially pathogenic CP missense variants were leveraged for an assessment of ACP prevalence based on real-world data. The resulting prevalence estimates were up to 20–25 fold higher than current ACP prevalence values (0.5/10^6^ in the Japanese population^9^; 0.4/10^6^ in the global population^10^). In an unbiased orthogonal approach (often employed to estimate prevalence of rare/ultra rare diseases based on gnomAD gene variants; e.g.,^10^^,^^62^, ^63^, ^64^, ^65^) prevalence estimates were also up to 16-fold higher than current ACP estimates, in broad agreement with the rationally obtained prevalence approximation.

Accurate prevalence estimates for autosomal recessive rare and ultra-rare diseases represent a challenge, particularly when no natural history studies are available to provide important elements such as disease penetrance, phenotype penetrance (important for pleiotropic phenotypes) and impact on mortality. In the absence of detailed patient demographics data, an approximation of prevalence may be achieved by analysing the number and geography of diagnosed patients based on published case reports, as recently reported for ACP.^6^ This approach exhibits numerous limitations, being affected by the low rate of diagnosis and long patient journeys associated with ultra-rare diseases, exacerbated by a lack of awareness of ACP and the pleiotropic nature of its clinical manifestations, which frequently results in misdiagnoses (e.g., as haemochromatosis or Wilson disease^6^^,^^66^). Also, the rate of diagnosis depends largely on the efficiency and organization of national Healthcare systems, which varies hugely amongst different geographies.^67^ The unprecedented availability of large-scale genomic information potentially represents a game-changer, as it provides information on all variants present in a population for a gene of interest irrespective of the limitations of diagnosis, as indeed implemented to estimate prevalence data for various ultra-rare diseases (e.g.,^10^^,^^62^, ^63^, ^64^, ^65^). In one of these studies focussing on NBIAs,^10^ the global lifetime risk of ACP was estimated at 0.4 × 10^6^ based on an earlier version of gnomAD (v2.1; comprising ca. 140 K genomes and exomes). These results were obtained by considering all the variants classified as “pathogenic” or “probably pathogenic” in the ClinVar and HGMD (Human Gene Mutation Database) databases up to 2021 and included mostly LoF variants from gnomAD v2.1 and their internal database, with only a small number of missense variants which however represent >59% of known ACP mutations.^6^ The higher ACP prevalence estimate values obtained in the present work may be explained by the larger number of alleles available in gnomAD 4.1.0 (1.6 M alleles from 807,162 individuals), ca. 5× the collective allele dataset considered previously, likely resulting in a smaller number of pathogenic/likely pathogenic CP variants available for previous prevalence estimates. Additionally, the use of a rational, experimental data-driven approach for selecting pathogenic/likely pathogenic CP variant alleles may have also contributed. While LoF mutations have predictable impacts on gene expression and/or function, missense variants still represent a challenge because their effect on the biological activity and/or expression of a protein is not immediately obvious. However, the potential impact of missense substitutions on a protein function can be addressed rationally if structural and functional information is known, as is the case for CP. For instance, a missense variant may have local or global impacts on protein function by altering a functionally important residue, active site, or structural features of the protein. If this information is available, it is possible to categorize the pathogenic potential of missense variants present in a population. For ACP, relying on the impact of missense variants on CP function to establish their pathogenicity is particularly important, as loss of CP activity is the only known fully penetrant phenotype, while the pleiotropic clinical manifestations may be misdiagnosed or overlooked (at least in the early stages of the disease). In the present study, the potential impact on CP enzymatic activity as a discriminator for ACP has been applied to predict the pathogenic potential of missense *CP* variants, resulting in the identification of 125 missense variants present in gnomAD v4.1.0. The robustness of this workflow was demonstrated by functional analysis of a subset of these variants in experiments aimed at determining their expression, secretion, and activity. This analysis wholly confirmed published data for all ACP missense variants analysed and resulted in a true positive rate of 81% and a true negative rate of 100%. Although a limitation of the study is represented by the relatively low number of variants experimentally analysed for sensitivity studies, the approach can demonstrably identify variants with a functional impact on CP, as well as those with no obvious impact on function (e.g., the p.G895A variant). These 125 missense *CP* variants with pathogenic potential, together with 177 LoF variants, three missense variants causing start/stop codon loss, 17 in-frame deletion/insertion, and 2 ACP missense variants (p.D77H and p.H343Y) constituted the dataset used for prevalence estimate predictions. Leveraging 324 CP variants from the latest gnomAD release (v4.1; n = 807,162), GeniE estimated a prevalence of 12.6/10^6^ in the general population. This is up to 25-fold greater than previous estimates, with values from 0.5/10^6^ in the Japanese population^9^ to 0.4/10^6^ variants in the general population (based on gnomAD v2.1; n = 141,456).^10^ GnomAD allows stratification based on the ethnic origin of genomes and exomes present in the database. In the gnomAD version employed in this study (v4), individuals of European ancestry represent 77% of the total cohort, which therefore biases prevalence estimates towards those found in individuals of European ancestry and may render estimates for other ethnic groups less representative (e.g., see^68^). Perhaps not surprisingly given the ethnic composition of gnomAD, the prevalence of CP pathogenic variants in the gnomAD cohort of European origin is 12.9/10^6^, similar to the global predicted prevalence of 12.6/10^6^. With the caveat of the gnomAD dataset underrepresenting ethnicities of non-European origin, the ethnic cohorts with the highest predicted prevalence of CP pathogenic variants are those from East Asia, Middle East and South Asia, with those presenting the lowest predicted prevalence represented by the Ashkenazi Jewish and African/African American populations. Interestingly, this study identifies the East Asian cohort as the ethnic group with the highest predicted prevalence of pathogenic CP variants in gnomAD, somewhat consistent with Japan being the country where ACP was first identified^69^ and with the highest number of diagnosed ACP patients.^6^ ACP prevalence estimates using an orthogonal, unbiased and widely used approach yielded overall prevalence estimates ranging from 6.64/10^6^ (most stringent threshold set; Table 3) to 21.6/10^6^ individuals (less stringent threshold set; Table 3). These estimates include the relatively more frequent p.G895A variant, for which there is conflicting evidence of pathogenicity and which in our *in-vitro* system did not impair CP function. This indicates that either its effects are manifested only *in vivo* through as yet uncharacterized mechanisms, or that other *CP* variants associated with p.G895A alleles are responsible for the ACP phenotypes in patients. If the p.G895A variant is removed from unbiased estimate calculations, estimates of 0.57 (based on the most stringent threshold) to 8.01/10^6^ (based on the less stringent threshold) are obtained. The prevalence estimate of 8.01/10^6^ is close to the global prevalence of 12.6/10^6^ calculated using the rational, structure-function informed approach (which did not include the pG895A variant frequencies as this was not amongst the 125 CP missense variants identified as potentially pathogenic by our *in-silico* analysis). To emphasize the significance of these findings, these estimates place ACP in a range of prevalence comparable to that of most rare bleeding disorders.^60^, ^61^, ^62^ Considering the experimentally analysed missense *CP* variants, these were selected as directly or closely associated with CP residues coordinating Cu^2+^ or Fe^2+^ ions (n = 5) and/or those associated with ACP (n = 7, as controls), together with examples of missense variants affecting the signal sequence of CP (n = 4). The selected missense CP variants have addressed three fundamental aspects of CP biology, namely i) CP copper loading, impacting correct folding and stability ii) CP enzymatic activity and iii) CP secretion/intracellular trafficking. This functional analysis focuses on the soluble form of CP, which is the major CP isoform expressed in the organism and one of the more abundant plasma proteins (https://www.proteinatlas.org/ENSG00000047457-CP/blood+protein). In fact, most published studies characterizing ACP mutant CP proteins from a functional perspective have so far focused on the GPI-anchored form of the protein.^18^, ^54^, ^55^ ACP has a clinical landscape characterised by both central nervous system (CNS) and peripheral phenotypes, including iron-restricted erythropoiesis leading to anaemia,^1^ and systemically delivered CP mitigates both CNS and non-CNS phenotypes in Cp-KO mice (a translational *in vivo* model of human ACP^15^^,^^16^). Therefore, it may be argued that functionally analysing the impact of pathogenic and potentially pathogenic CP variants on the soluble form of the protein constitutes a more pathologically relevant approach than focusing solely on the GPI-anchored form. Indeed, CP plasma levels represent a key diagnostic marker for ACP.^57^ Missense CP variants can cause pathogenicity by impairing correct CP copper loading and hence folding, stability, and activity (e.g., variants p.C338S and p.G650R) as well as by impairing CP secretion (e.g., variants p.D77H and p.A350D). In fact, decreased CP plasma levels due to impairment of CP copper loading is eminently demonstrated in Wilson disease, where variants in the copper loading transporter ATP7b result in the expression of an incorrectly folded CP and its rapid degradation (e.g., see^70^). Aside from impacting copper loading, missense CP variants could impact residues important for enzymatic activity resulting in normal or near normal CP plasma protein levels but decreased specific ferroxidase/oxidase activity. To date, no ACP variant specifically leading to an impairment of Ox/FeOx activity (i.e., without a concomitant impact on CP folding and protein levels) has been identified. It is noteworthy that the rational approach presented here has identified a CP missense variant present in the population (p.E990D; impacting a residue directly coordinating Fe^2+^), with apparently normal CP expression, secretion, and copper incorporation (as assessed by Holo-CP formation), but displaying a significant impairment of specific enzymatic activity (down to 50% of WT rCP). With respect to *CP* variants impacting CP activity but not folding/expression, the p.D1044N missense variant is of further interest. Its apparent selectivity for FeOx activity, with little or no effect on Ox activity, suggests that p.D1044N may influence CP activity on a subset of its substrates, differently from the p.E990D variant which impacts both FeOx and Ox CP activity. Interestingly, separate substrate binding sites have been identified in CP for biogenic amines and aromatic amines.^71^ D1044 was identified as a residue implicated in the binding site for biogenic amines and is one of three residues implicated in coordinating Fe^2+^ binding (FeOx assay substrate; Fig. 2a). Conversely, D1044 has no apparent involvement in the structurally distant binding site for aromatic amines, bound by p-phenylenediamine which is the Ox assay substrate.^71^ This may explain the apparent selectivity of the p.D1044N for the FeOX assay relative to the Ox assay. If experimentally confirmed on CP’s proposed non-Fe^2+^ natural substrates (e.g., biogenic and aromatic amines^71^), p.D1044N would represent the first reported example of a natural CP variant able to impact CP activity in a substrate-selective fashion, with implications as to the role of such substrate selectivity in CP’s biology.

As low/absent CP levels in plasma are currently used to support ACP diagnosis,^57^ the measurement of CP levels alone would identify carriers of CP variants leading to a lack of protein expression (including impaired copper loading, as this CP would be unstable and degraded), but would fail to identify patients with variants resulting in unaltered CP levels (e.g., copper-loading proficient) but impaired enzymatic activity. Thus, measurement of CP-specific activity, as performed throughout the present work, would provide a more accurate diagnostic tool for ACP diagnosis.^57^ The other functionally analysed missense CP variants displayed different levels of functional performance, ranging from little or no impairment of secretion or enzymatic activity, to almost complete loss of protein function (p.H343Y and p.H656R), similar to missense mutants previously linked to ACP (p.C338S, p.G650R, p.G708S, and p.G988S). Importantly, the p.H343Y and p.H656R missense variants could produce an ACP phenotype if present in an individual as homozygous or compound heterozygous states with other LoF CP variants, as these mutants fail to incorporate Cu^2+^ during CP biosynthesis, leading to the secretion of Apo-CP, a protein that is inactive and has a short half-life in plasma.^72^ The p.H343Y and p.H656R mutants, whose pathogenic potential is we believe reported here for the first time, are located in the type I Cu^2+^binding sites of domains 2 and 4 (respectively), which were previously thought to be non-essential for CP enzymatic activity.^50^^,^^51^ The present study critically demonstrates that even CP residues associated with Cu^2+^ type I ion coordination are critical for Cu^2+^ incorporation and enzymatic activity of CP (without impacting protein secretion), thereby broadening our understanding of this protein. Aside from copper loading/correct folding and enzymatic activity, adequate secretion of CP is critical for attaining physiological levels of circulating CP and oxidase/ferroxidase activity. Significantly, the data presented here confirmed previous findings of substantial intracellular accumulation and impaired secretion due to ER-to-Golgi trafficking of two ACP variants, p.D77H and p.A350D.^56^ The SEL1L-HRD1 protein complex, which is part of the endoplasmic reticulum ER-associated degradation process, has been shown to degrade disease-causing mutants of CP (i.e., p.G195R, p.G625E, p.G892E) and prevent the formation of high-molecular-weight CP aggregates.^73^ It is possible that this mechanism intervenes in the degradation of p.D77H and p.A350D, as their CP concentrations in the media were too low to assess their specific activity in spite of clearly detectable intracellular expression. It has been reported that CP in the sera of patients with homozygous p.A350D is barely detectable, and a complete lack of serum CP was reported for compound heterozygous p.D77H/p.Q711K.^19^^,^^74^ Therefore, even if fully functional, the residual circulating CP from these mutants would be insufficient. CP secretion was also impaired in two uncharacterized missense variants impacting the signal peptide (p.A17D and p.A19V, predicted to alter the cleavage site of the mature CP protein) as well as in the p.G895A variant. Variations in CP secretion efficiency, as presented here for these variants, could contribute to the variability in plasma CP levels observed in the normal population.^75^ As for the p.G895A variant, conflicting reports have emerged in the literature regarding its pathogenicity^20^^,^^54^^,^^56^^,^^76^; however, this variant apparently displays normal Holo-CP formation and catalytic activity, consistent with a benign classification.

An important limitation of this study concerns the difficulty in including the impact of disease penetrance and mortality on prevalence estimates based on population frequencies of CP pathogenic alleles. While adequately powered natural history studies in ACP are not available to address penetrance and mortality, certain inferences can be drawn based on existing knowledge of the disease and published ACP case reports. ACP can be considered fully penetrant if the primary discriminating phenotype causing the clinically significant symptoms is taken into account (i.e., loss of CP function due to structural or missense mutations in the CP gene, causing neurological, hepatic, haematological, diabetes and retinal degeneration.^6^^,^^77^; These clinical phenotypes are present (often simultaneously) in all ACP patients, which are diagnosed based on the presence in a combination of the symptoms.^77^ Given that progressive iron accumulation in various tissues including brain and liver is invariably associated with ACP,^78^ it is plausible to assume that a majority of individuals diagnosed with ACP on the basis of non-neurological symptoms will eventually develop neurological symptoms as time progresses, consistent with available patient data,^6^ particularly because iron chelation therapy does not address these symptoms.^77^, ^78^, ^79^ It is therefore reasonable to assume that the penetrance of neurological symptoms in ACP may be very high, with most ACP patients eventually presenting with progressive neurological deterioration caused by iron accumulation in the brain. This is somewhat supported by the only longitudinal study of a small set of ACP patients^8^ as well as by case reports analyses indicating that >70% of ACP patients diagnosed for systemic symptoms eventually develop neurological symptoms.^6^ As for mortality, the absence of adequately powered longitudinal studies is again an issue.^77^ However, ACP is described as a potentially fatal disease,^80^ as progressive iron accumulation in the brain and other tissues can cause premature death due to severe neurodegeneration as well as heart or liver dysfunction, as described for NBIA disorders.^81^ In ACP, prognosis is considered poor once neurological symptoms ensue,^21^ and a diagnosis of ACP can therefore be reasonably assumed to increase the probability of premature mortality. Thus, it is plausible to assume that CP variants leading to loss of CP activity result in a high penetrance in terms of clinical symptoms, with a substantial impact on the life quality and life expectancy of affected individuals, particularly when neurological symptoms ensue. For pathogenic missense variants, the impact on penetrance and mortality is likely to be similar to that of structural variants when the resulting effect is loss of CP function, as for instance is the case for a majority of the known and novel CP pathogenic missense variants experimentally validated in the present study. The impact of missense CP variants demonstrating a partial loss of function on symptoms penetrance and mortality is less clear, and this represents a potential limitation for the prevalence estimates presented in this study. However, it is noteworthy that our *in-silico* prioritization of potentially pathogenic missense CP variants correctly excluded the p.E990D and p.D1044N *CP* variants, which our experimental analysis showed to cause partial loss of function, as well as the p.G895A which in our hands had no effect on CP activity. This suggests that, at least to some extent, our approach can meaningfully discern between complete and partial loss of function and that prevalence estimates presented in this work are likely based mostly on missense *CP* variants with a robust impact on *CP* function.

Clearly, the estimation of disease prevalence for ultra-rare diseases based on rare variant allele frequencies is affected by many variables (e.g., sampling variance, differences in algorithms and pathogenicity prediction tools in terms of sensitivity and specificity, lack of sufficient characterization in terms of disease penetrance and mortality). However, and in conclusion, in spite of its limitations this study represents a significant advancement in understanding the genetic variability of CP in relation to ACP susceptibility and highlights the need for standardization in the toolkit used to study CP expression and function. As for many rare diseases, the lack of commutable reference proteins,^82^, ^83^, ^84^ qualified antibodies (see for instance Supplementary Fig. S2) and standardized assays represent an additional challenge, particularly for clinicians working with patients, as well as for drug discovery efforts aimed at identifying therapies. The ACP community may benefit from the experience gained in other rare neurodegenerative diseases where standardized, qualified toolkits and assays have been developed, qualified and made available to the research community (e.g., in Huntington’s disease; https://chdifoundation.org/research-tools-reagents/), with similar efforts implemented in more common neurodegenerative conditions (e.g., in Parkinson’s disease; https://www.michaeljfox.org/research-tools).

## Contributors

NZ and AC conceived the study; NZ and SL performed the experiments and analysed the data; AZ and MA performed immunofluorescence analysis; MC performed *in-silico* structural analysis; NZ, GS, and RA performed prevalence analysis; NZ and AC performed data curation and wrote the manuscript; EDP and AC provided resources and funding for the investigation; AC supervised the study. NZ, RA and AC have accessed and verified the underlying data. All the authors read and approved the final version of the manuscript.

## Data sharing statement

All data are available in the main text or the Supplementary material.

## Declaration of interests

The authors declare that they have no conflict of interest. AC discloses that the study was partially supported by Kedrion S.p.A. within the framework of scientific collaboration. AC and SL are employees of Kedrion S.p.A.

## Appendix ASupplementary data

Figures_WB_uncropped

Supplementary Table S4

Reagent validation file

## References

[1] Kono S. (2012). Aceruloplasminemia. Curr Drug Targets.

[2] Harris Z.L., Takahashi Y., Miyajima H., Serizawa M., MacGillivray R.T., Gitlin J.D. (1995). Aceruloplasminemia: molecular characterization of this disorder of iron metabolism. Proc Natl Acad Sci USA.

[3] Patel B.N., Dunn R.J., David S. (2000). Alternative RNA splicing generates a glycosylphosphatidylinositol-anchored form of ceruloplasmin in mammalian brain. J Biol Chem.

[4] Bielli P., Calabrese L. (2002). Structure to function relationships in ceruloplasmin: a 'moonlighting' protein. Cell Mol Life Sci.

[5] Vasilyev V.B. (2019). Looking for a partner: ceruloplasmin in protein-protein interactions. Biometals.

[6] Ketata I., Ellouz E. (2023). New view of aceruloplasminemia: systematic review and meta-analysis tracking dots from onset to disease development and iron-related features. Rare.

[7] Piperno A., Alessio M. (2018). Aceruloplasminemia: waiting for an efficient therapy. Front Neurosci.

[8] Pelucchi S., Mariani R., Ravasi G. (2018). Phenotypic heterogeneity in seven Italian cases of aceruloplasminemia. Parkinsonism Relat Disord.

[9] Miyajima H., Kohno S., Takahashi Y., Yonekawa O., Kanno T. (1999). Estimation of the gene frequency of aceruloplasminemia in Japan. Neurology.

[10] Kolarova H., Tan J., Strom T.M., Meitinger T., Wagner M., Klopstock T. (2022). Lifetime risk of autosomal recessive neurodegeneration with brain iron accumulation (NBIA) disorders calculated from genetic databases. EBioMedicine.

[11] Martin Merinero H., Zhang Y., Arjona E. (2021). Functional characterization of 105 factor H variants associated with aHUS: lessons for variant classification. Blood.

[12] Gudmundsson S., Singer-Berk M., Watts N.A. (2022). Variant interpretation using population databases: lessons from gnomAD. Hum Mutat.

[13] Yonekawa M., Okabe T., Asamoto Y., Ohta M. (1999). A case of hereditary ceruloplasmin deficiency with iron deposition in the brain associated with chorea, dementia, diabetes mellitus and retinal pigmentation: administration of fresh-frozen human plasma. Eur Neurol.

[14] Poli L., Alberici A., Buzzi P. (2017). Is aceruloplasminemia treatable? Combining iron chelation and fresh-frozen plasma treatment. Neurol Sci.

[15] Zanardi A., Conti A., Cremonesi M. (2018). Ceruloplasmin replacement therapy ameliorates neurological symptoms in a preclinical model of aceruloplasminemia. EMBO Mol Med.

[16] Zanardi A., Nardini I., Raia S. (2024). New orphan disease therapies from the proteome of industrial plasma processing waste- a treatment for aceruloplasminemia. Commun Biol.

[17] Kalman B., Lautenschlaeger R., Kohlmayer F. (2012). An international registry for neurodegeneration with brain iron accumulation. Orphanet J Rare Dis.

[18] Kono S., Suzuki H., Oda T. (2006). Biochemical features of ceruloplasmin gene mutations linked to aceruloplasminemia. NeuroMolecular Med.

[19] Perez-Aguilar F., Burguera J.A., Benlloch S., Berenguer M., Rayon J.M. (2005). Aceruloplasminemia in an asymptomatic patient with a new mutation. Diagnosis and family genetic analysis. J Hepatol.

[20] Jimenez-Huete A., Bernar J., Miyajima H. (2008). Multiple motor system dysfunction associated with a heterozygous ceruloplasmin gene mutation. J Neurol.

[21] Vroegindeweij L.H.P., Langendonk J.G., Langeveld M. (2017). New insights in the neurological phenotype of aceruloplasminemia in Caucasian patients. Parkinsonism Relat Disord.

[22] Daimon M., Susa S., Ohizumi T. (2000). A novel mutation of the ceruloplasmin gene in a patient with heteroallelic ceruloplasmin gene mutation (HypoCPGM). Tohoku J Exp Med.

[23] Bosio S., De Gobbi M., Roetto A. (2002). Anemia and iron overload due to compound heterozygosity for novel ceruloplasmin mutations. Blood.

[24] Hellman N.E., Kono S., Miyajima H., Gitlin J.D. (2002). Biochemical analysis of a missense mutation in aceruloplasminemia. J Biol Chem.

[25] Lobbes H., Reynaud Q., Mainbourg S. (2022). A new pathogenic missense variant in a consanguineous north-African family responsible for a highly variable aceruloplasminemia phenotype: a case-report. Front Neurosci.

[26] Shang H.F., Jiang X.F., Burgunder J.M., Chen Q., Zhou D. (2006). Novel mutation in the ceruloplasmin gene causing a cognitive and movement disorder with diabetes mellitus. Mov Disord.

[27] Vila Cuenca M., Marchi G., Barque A. (2020). Genetic and clinical heterogeneity in thirteen new cases with aceruloplasminemia. Atypical anemia as a clue for an early diagnosis. Int J Mol Sci.

[28] Hines M.C., Bonkovsky H.L., Rudnick S.R., Mhoon J.T. (2018). Peripheral neuropathy and the ceruloplasmin gene. Ann Intern Med.

[29] Corradini E., Buzzetti E., Dongiovanni P. (2021). Ceruloplasmin gene variants are associated with hyperferritinemia and increased liver iron in patients with NAFLD. J Hepatol.

[30] Ondrejkovicova M., Drazilova S., Drakulova M. (2020). New mutation of the ceruloplasmin gene in the case of a neurologically asymptomatic patient with microcytic anaemia, obesity and supposed Wilson's disease. BMC Gastroenterol.

[31] Yamamura A., Kikukawa Y., Tokunaga K. (2018). Pancytopenia and myelodysplastic changes in aceruloplasminemia: a case with a novel pathogenic variant in the ceruloplasmin gene. Intern Med.

[32] Di Raimondo D., Pinto A., Tuttolomondo A., Fernandez P., Camaschella C., Licata G. (2008). Aceruloplasminemia: a case report. Internal and Emergency. Medicine.

[33] Lindner U., Schuppan D., Schleithoff L. (2014). Aceruloplasminaemia: a family with a novel mutation and long-term therapy with deferasirox. Horm Metab Res.

[34] Kuhn J., Miyajima H., Takahashi Y. (2005). Extrapyramidal and cerebellar movement disorder in association with heterozygous ceruloplasmin gene mutation. J Neurol.

[35] Kono S., Suzuki H., Takahashi K. (2006). Hepatic iron overload associated with a decreased serum ceruloplasmin level in a novel clinical type of aceruloplasminemia. Gastroenterology.

[36] Takeuchi Y., Yoshikawa M., Tsujino T. (2002). A case of aceruloplasminaemia: abnormal serum ceruloplasmin protein without ferroxidase activity. J Neurol Neurosurg Psychiatry.

[37] Chen S., Francioli L.C., Goodrich J.K. (2023). A genomic mutational constraint map using variation in 76,156 human genomes. Nature.

[38] Landrum M.J., Chitipiralla S., Brown G.R. (2020). ClinVar: improvements to accessing data. Nucleic Acids Res.

[39] Teufel F., Almagro Armenteros J.J., Johansen A.R. (2022). SignalP 6.0 predicts all five types of signal peptides using protein language models. Nat Biotechnol.

[40] Curzon G., Vallet L. (1960). The purification of human caeruloplasmin. Biochem J.

[41] Erel O. (1998). Automated measurement of serum ferroxidase activity. Clin Chem.

[42] Chen Y., Lu H., Zhang N., Zhu Z., Wang S., Li M. (2020). PremPS: predicting the impact of missense mutations on protein stability. PLoS Comput Biol.

[43] Zhou Y., Pan Q., Pires D.E.V., Rodrigues C.H.M., Ascher D.B. (2023). DDMut: predicting effects of mutations on protein stability using deep learning. Nucleic Acids Res.

[44] Pires D.E., Ascher D.B., Blundell T.L. (2014). DUET: a server for predicting effects of mutations on protein stability using an integrated computational approach. Nucleic Acids Res.

[45] Rodrigues C.H.M., Pires D.E.V., Ascher D.B. (2021). DynaMut2: assessing changes in stability and flexibility upon single and multiple point missense mutations. Protein Sci.

[46] Dehouck Y., Kwasigroch J.M., Gilis D., Rooman M. (2011). PoPMuSiC 2.1: a web server for the estimation of protein stability changes upon mutation and sequence optimality. BMC Bioinf.

[47] Varadi M., Anyango S., Deshpande M. (2022). AlphaFold Protein Structure Database: massively expanding the structural coverage of protein-sequence space with high-accuracy models. Nucleic Acids Res.

[48] Pejaver V., Byrne A.B., Feng B.J. (2022). Calibration of computational tools for missense variant pathogenicity classification and ClinGen recommendations for PP3/BP4 criteria. Am J Hum Genet.

[49] Musci G., Bonaccorsi di Patti M.C., Calabrese L. (1993). The state of the copper sites in human ceruloplasmin. Arch Biochem Biophys.

[50] Calabrese L., Carbonaro M., Musci G. (1989). Presence of coupled trinuclear copper cluster in mammalian ceruloplasmin is essential for efficient electron transfer to oxygen. J Biol Chem.

[51] Sedlak E., Wittung-Stafshede P. (2007). Discrete roles of copper ions in chemical unfolding of human ceruloplasmin. Biochemistry.

[52] Perlman D., Halvorson H.O. (1983). A putative signal peptidase recognition site and sequence in eukaryotic and prokaryotic signal peptides. J Mol Biol.

[53] von Heijne G. (1985). Signal sequences. The limits of variation. J Mol Biol.

[54] di Patti M.C., Maio N., Rizzo G. (2009). Dominant mutants of ceruloplasmin impair the copper loading machinery in aceruloplasminemia. J Biol Chem.

[55] Maio N., Polticelli F., De Francesco G., Rizzo G., Bonaccorsi di Patti M.C., Musci G. (2010). Role of external loops of human ceruloplasmin in copper loading by ATP7B and Ccc2p. J Biol Chem.

[56] Kono S., Yoshida K., Tomosugi N. (2010). Biological effects of mutant ceruloplasmin on hepcidin-mediated internalization of ferroportin. Biochim Biophys Acta.

[57] Miyajima H., Hosoi Y., Adam M.P., Feldman J., Mirzaa G.M. (1993). GeneReviews((R)).

[58] Mak C.M., Lam C.W. (2008). Diagnosis of Wilson's disease: a comprehensive review. Crit Rev Clin Lab Sci.

[59] Hellman N.E., Kono S., Mancini G.M., Hoogeboom A.J., De Jong G.J., Gitlin J.D. (2002). Mechanisms of copper incorporation into human ceruloplasmin. J Biol Chem.

[60] Asselta R., Paraboschi E.M., Rimoldi V. (2017). Exploring the global landscape of genetic variation in coagulation factor XI deficiency. Blood.

[61] Paraboschi E.M., Duga S., Asselta R. (2017). Fibrinogen as a pleiotropic protein causing human diseases: the mutational burden of aalpha, bbeta, and gamma chains. Int J Mol Sci.

[62] Seidizadeh O., Cairo A., Baronciani L., Valenti L., Peyvandi F. (2023). Population-based prevalence and mutational landscape of von Willebrand disease using large-scale genetic databases. NPJ Genom Med.

[63] Seidizadeh O., Cairo A., Mancini I., George J.N., Peyvandi F. (2024). Global prevalence of hereditary thrombotic thrombocytopenic purpura determined by genetic analysis. Blood Adv.

[64] Kurtovic-Kozaric A., Singer-Berk M., Wood J. (2024). An estimation of global genetic prevalence of PLA2G6-associated neurodegeneration. Orphanet J Rare Dis.

[65] Lake N.J., Phua J., Liu W., Moors T., Axon S., Lek M. (2023). Estimating the prevalence of LAMA2 congenital muscular dystrophy using population genetic databases. J Neuromuscul Dis.

[66] Kerkhof M., Honkoop P. (2014). Never forget aceruloplasminemia in case of highly suggestive Wilson's disease score. Hepatology.

[67] Schutte S., Acevedo P.N.M., Flahault A. (2018). Health systems around the world - a comparison of existing health system rankings. J Glob Health.

[68] Venner E., Patterson K., Kalra D. (2024). The frequency of pathogenic variation in the All of Us cohort reveals ancestry-driven disparities. Commun Biol.

[69] Miyajima H., Nishimura Y., Mizoguchi K., Sakamoto M., Shimizu T., Honda N. (1987). Familial apoceruloplasmin deficiency associated with blepharospasm and retinal degeneration. Neurology.

[70] Czlonkowska A., Litwin T., Dusek P. (2018). Wilson disease. Nat Rev Dis Primers.

[71] Zaitsev V.N., Zaitseva I., Papiz M., Lindley P.F. (1999). An X-ray crystallographic study of the binding sites of the azide inhibitor and organic substrates to ceruloplasmin, a multi-copper oxidase in the plasma. J Biol Inorg Chem.

[72] Holtzman N.A., Gaumnitz B.M. (1970). Studies on the rate of release and turnover of ceruloplasmin and apoceruloplasmin in rat plasma. J Biol Chem.

[73] Thepsuwan P., Bhattacharya A., Song Z. (2023). Hepatic SEL1L-HRD1 ER-associated degradation regulates systemic iron homeostasis via ceruloplasmin. Proc Natl Acad Sci USA.

[74] Hofmann W.P., Welsch C., Takahashi Y. (2007). Identification and in silico characterization of a novel compound heterozygosity associated with hereditary aceruloplasminemia. Scand J Gastroenterol.

[75] Xu R., Jiang Y.F., Zhang Y.H., Yang X. (2018). The optimal threshold of serum ceruloplasmin in the diagnosis of Wilson's disease: a large hospital-based study. PLoS One.

[76] Lanktree M.B., Sadikovic B., Waye J.S. (2017). Clinical evaluation of a hemochromatosis next-generation sequencing gene panel. Eur J Haematol.

[77] Marchi G., Busti F., Lira Zidanes A., Castagna A., Girelli D. (2019). Aceruloplasminemia: a severe neurodegenerative disorder deserving an early diagnosis. Front Neurosci.

[78] Finkenstedt A., Wolf E., Hofner E. (2010). Hepatic but not brain iron is rapidly chelated by deferasirox in aceruloplasminemia due to a novel gene mutation. J Hepatol.

[79] Vroegindeweij L.H.P., Boon A.J.W., Wilson J.H.P., Langendonk J.G. (2020). Effects of iron chelation therapy on the clinical course of aceruloplasminemia: an analysis of aggregated case reports. Orphanet J Rare Dis.

[80] Corradini E., Buzzetti E., Pietrangelo A. (2020). Genetic iron overload disorders. Mol Aspects Med.

[81] Wydrych A., Pakula B., Janikiewicz J. (2025). Metabolic impairments in neurodegeneration with brain iron accumulation. Biochim Biophys Acta Bioenerg.

[82] Zegers I., Beetham R., Keller T. (2013). The importance of commutability of reference materials used as calibrators: the example of ceruloplasmin. Clin Chem.

[83] Whicher J.T. (1998). BCR/IFCC reference material for plasma proteins (CRM 470). Community bureau of reference. International federation of clinical chemistry. Clin Biochem.

[84] Infusino I., Valente C., Dolci A., Panteghini M. (2010). Standardization of ceruloplasmin measurements is still an issue despite the availability of a common reference material. Anal Bioanal Chem.

