# Supplementary material for: Functional characterisation of missense ceruloplasmin variants and real-world prevalence assessment of Aceruloplasminemia using population data
Source: eBioMedicine. 2025 Mar 4;113:105625. doi: 10.1016/j.ebiom.2025.105625 (PMC11927744; doi:10.1016/j.ebiom.2025.105625)
Supplement: Figures_WB_uncropped [file mmc1.pptx]

## Slide 1
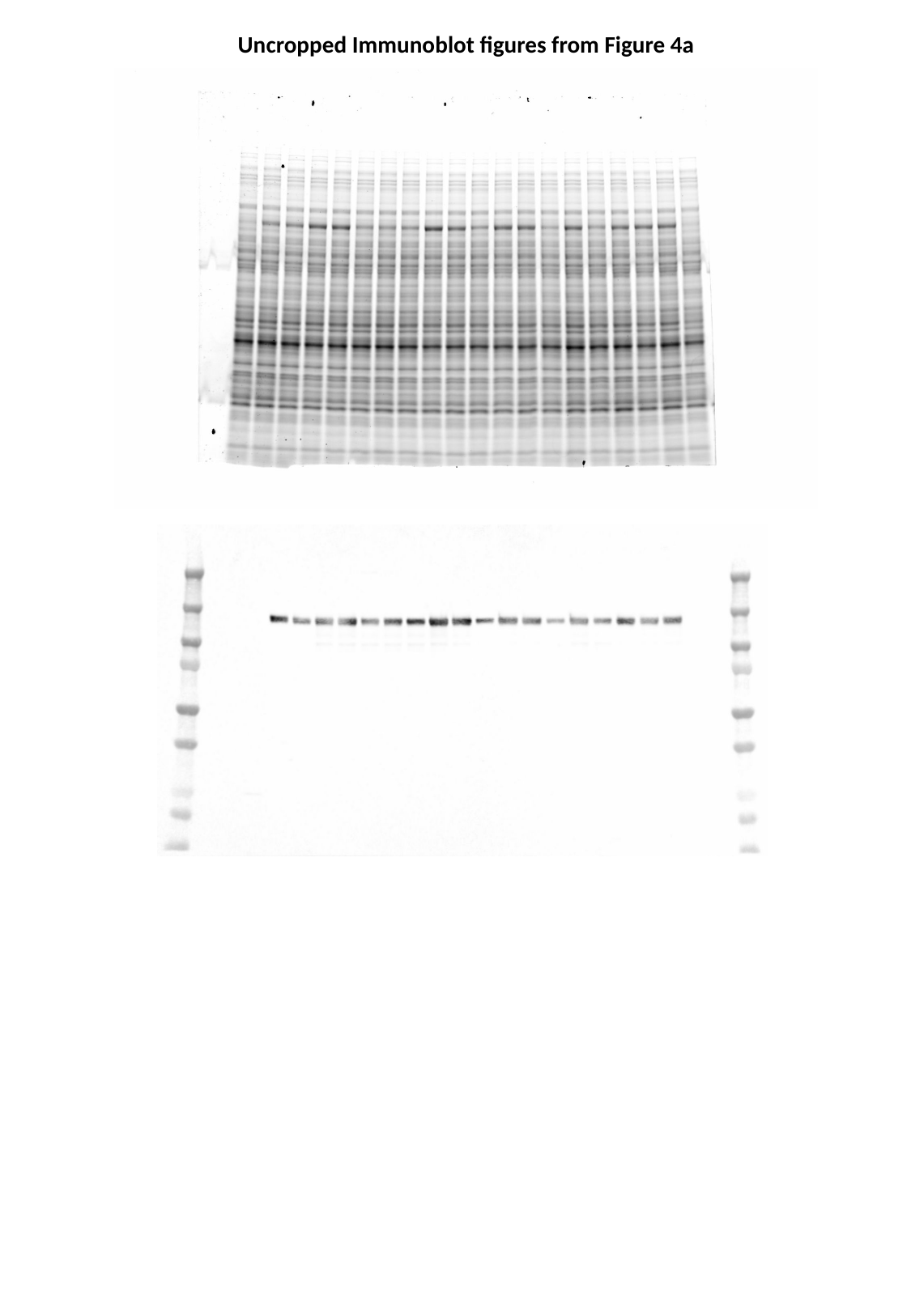

Uncropped Immunoblot figures from Figure 4a

## Slide 2
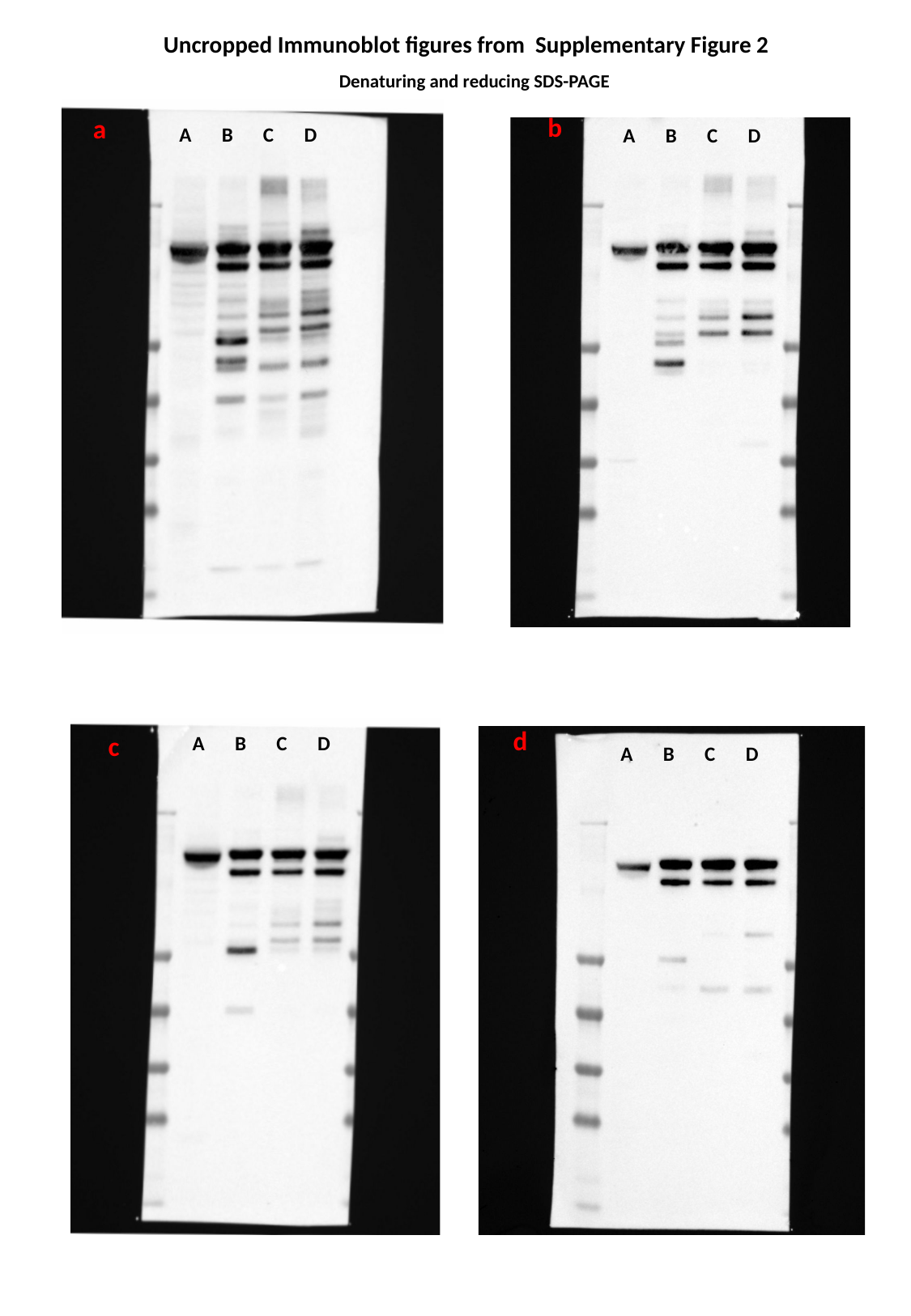

Uncropped Immunoblot figures from Supplementary Figure 2
Denaturing and reducing SDS-PAGE
a
A
B
C
D
b
A
B
C
D
c
A
B
C
D
d
A
B
C
D

## Slide 3
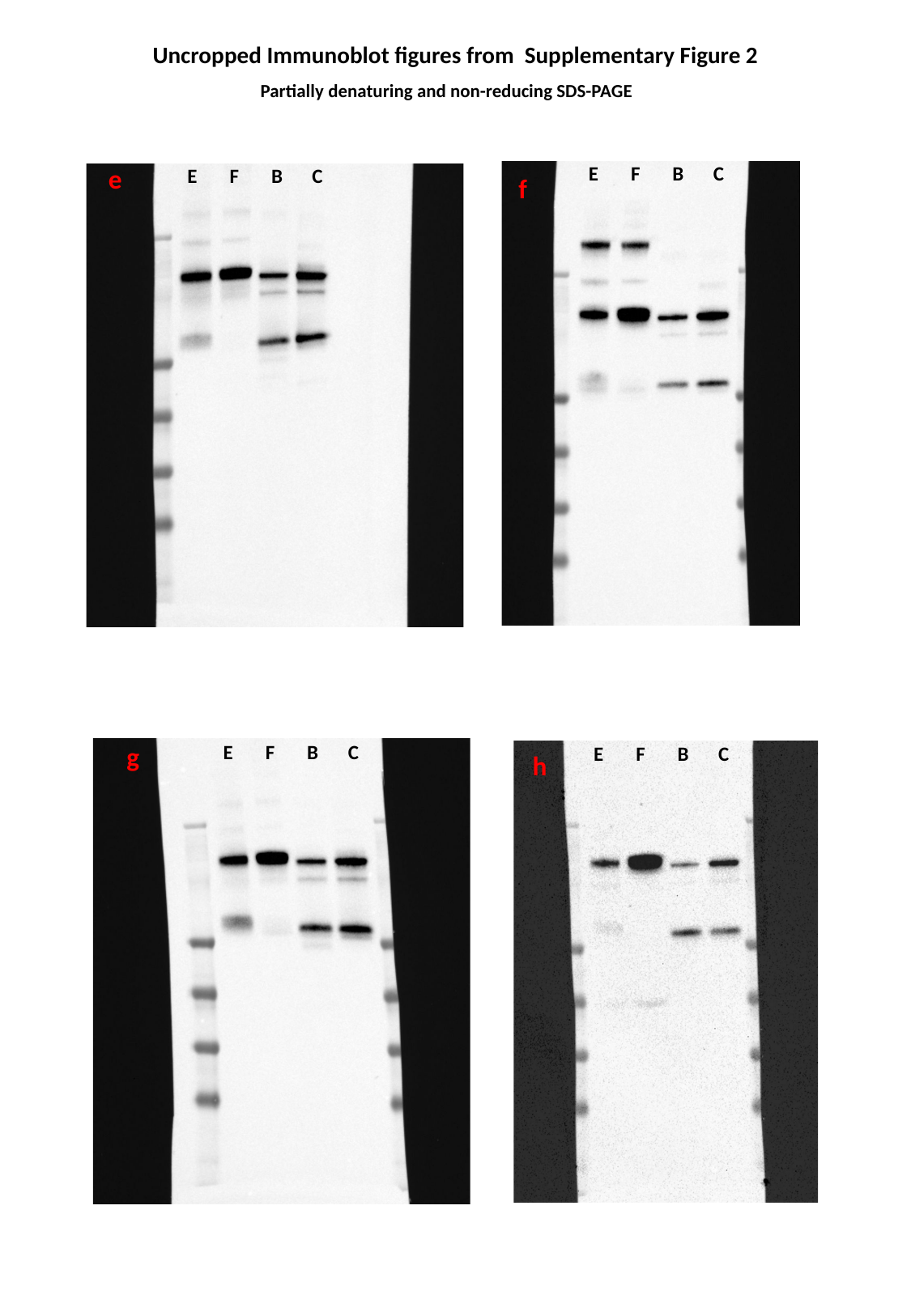

Uncropped Immunoblot figures from Supplementary Figure 2
Partially denaturing and non-reducing SDS-PAGE
E
F
B
C
f
e
E
F
B
C
E
F
B
C
g
E
F
B
C
h
