# Supplementary material for: Functional characterisation of missense ceruloplasmin variants and real-world prevalence assessment of Aceruloplasminemia using population data
Source: eBioMedicine. 2025 Mar 4;113:105625. doi: 10.1016/j.ebiom.2025.105625 (PMC11927744; doi:10.1016/j.ebiom.2025.105625)
Supplement: Supplementary Figs. S1 and S2 [file mmc2.pptx]

## Slide 1
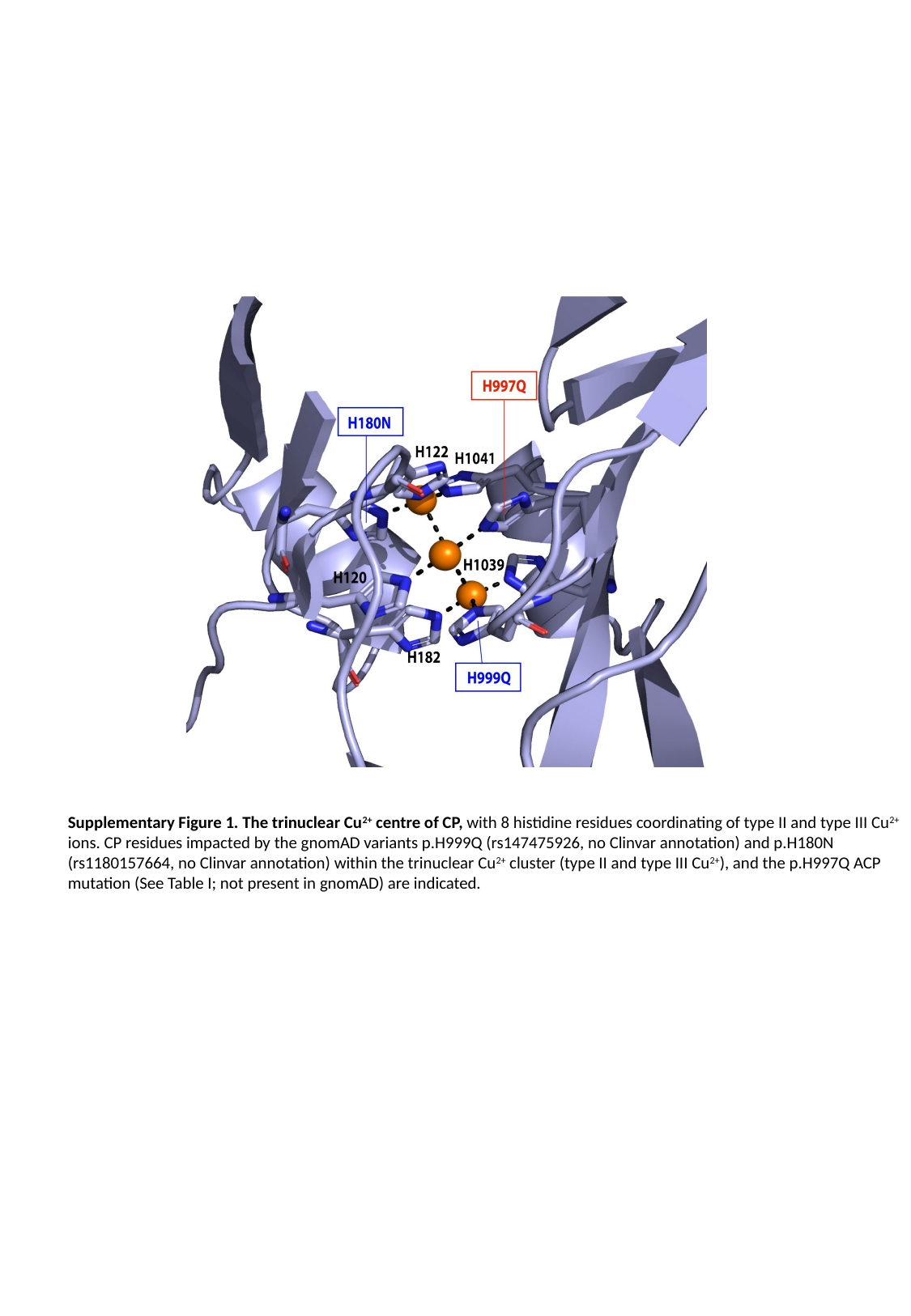

Supplementary Figure 1. The trinuclear Cu2+ centre of CP, with 8 histidine residues coordinating of type II and type III Cu2+ ions. CP residues impacted by the gnomAD variants p.H999Q (rs147475926, no Clinvar annotation) and p.H180N (rs1180157664, no Clinvar annotation) within the trinuclear Cu2+ cluster (type II and type III Cu2+), and the p.H997Q ACP mutation (See Table I; not present in gnomAD) are indicated.

## Slide 2
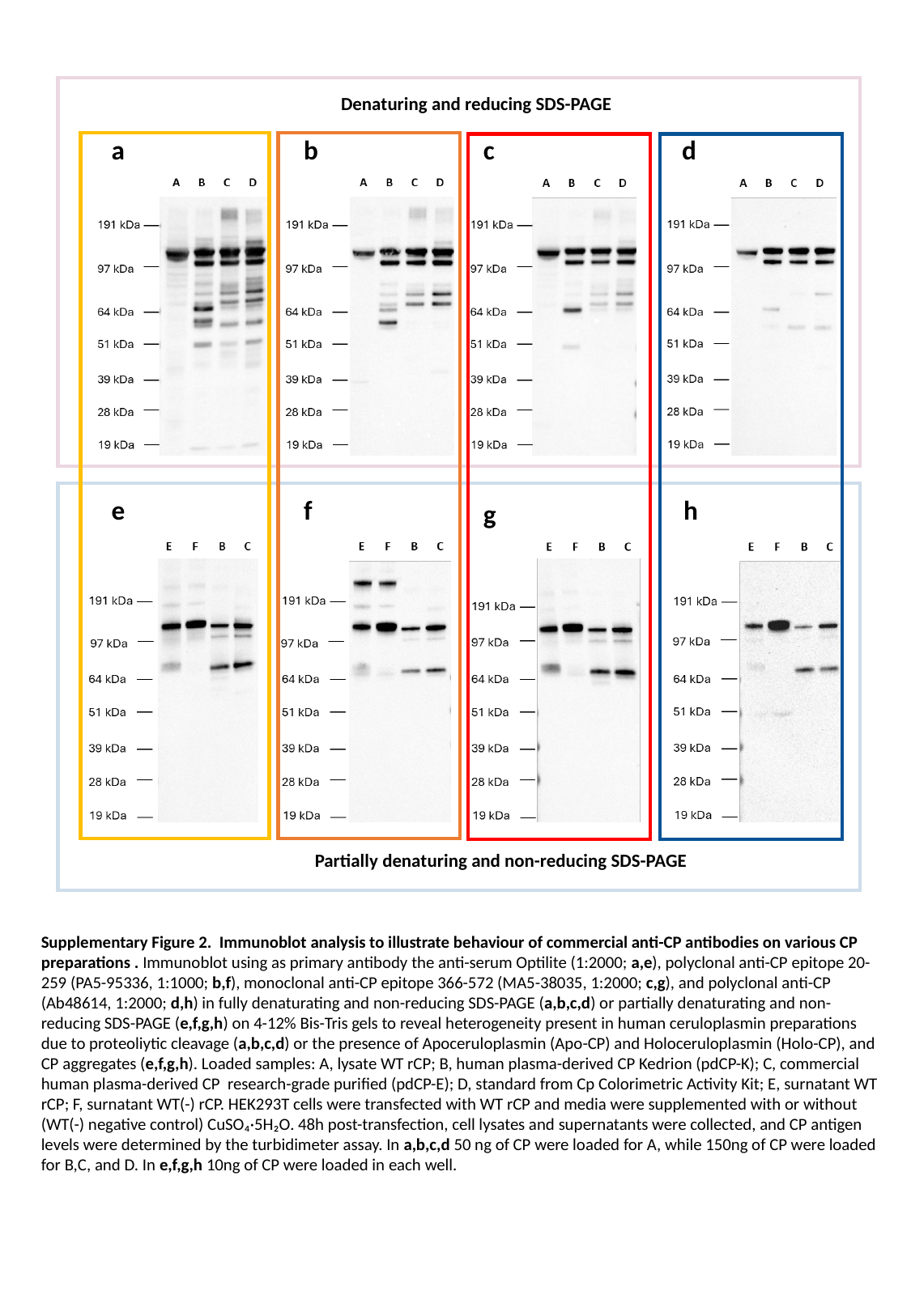

Denaturing and reducing SDS-PAGE
a
b
c
d
e
f
h
g
Partially denaturing and non-reducing SDS-PAGE
Supplementary Figure 2. Immunoblot analysis to illustrate behaviour of commercial anti-CP antibodies on various CP preparations . Immunoblot using as primary antibody the anti-serum Optilite (1:2000; a,e), polyclonal anti-CP epitope 20-259 (PA5-95336, 1:1000; b,f), monoclonal anti-CP epitope 366-572 (MA5-38035, 1:2000; c,g), and polyclonal anti-CP (Ab48614, 1:2000; d,h) in fully denaturating and non-reducing SDS-PAGE (a,b,c,d) or partially denaturating and non-reducing SDS-PAGE (e,f,g,h) on 4-12% Bis-Tris gels to reveal heterogeneity present in human ceruloplasmin preparations due to proteoliytic cleavage (a,b,c,d) or the presence of Apoceruloplasmin (Apo-CP) and Holoceruloplasmin (Holo-CP), and CP aggregates (e,f,g,h). Loaded samples: A, lysate WT rCP; B, human plasma-derived CP Kedrion (pdCP-K); C, commercial human plasma-derived CP research-grade purified (pdCP-E); D, standard from Cp Colorimetric Activity Kit; E, surnatant WT rCP; F, surnatant WT(-) rCP. HEK293T cells were transfected with WT rCP and media were supplemented with or without (WT(-) negative control) CuSO₄·5H₂O. 48h post-transfection, cell lysates and supernatants were collected, and CP antigen levels were determined by the turbidimeter assay. In a,b,c,d 50 ng of CP were loaded for A, while 150ng of CP were loaded for B,C, and D. In e,f,g,h 10ng of CP were loaded in each well.
