# Supplementary material for: Functional characterisation of missense ceruloplasmin variants and real-world prevalence assessment of Aceruloplasminemia using population data
Source: eBioMedicine. 2025 Mar 4;113:105625. doi: 10.1016/j.ebiom.2025.105625 (PMC11927744; doi:10.1016/j.ebiom.2025.105625)
Supplement: Reagent validation file [file mmc7.pdf]

Reagent Validation File

Relevant reagent information and RRID tags are listed below. All antibodies used in our study are commercially available antibodies.

Cell line/Antibody	Company	Cat. No	RRID
HEK293T	ATCC	#CRL-11268	**CVCL_1926**
CP monoclonal antibody	Thermo Fisher Scientific	#MA5-38035	**AB_2897953**
CP polyclonal antibody	Thermo Fisher Scientific	#PA5-95336	**AB_2807139**
polyclonal anti-Cp	Abcam	#ab48614	**AB_869113**
rabbit anti-sheep HRP	Thermo Fisher Scientific	#31480	**AB_228457**
donkey anti-rabbit HRP	Cytiva	#NA9340	**AB_772191**
rabbit anti-Cp	BioVision	#7019	**AB_3665653**
rat anti-GRP94	Enzo Life Sciences	#ADI-SPA-850-D	**AB_2039133**
mouse anti-GM130	BD Biosciences	#610822	**AB_398141**
Goat anti-rabbit Alexa Fluor-594	Thermo Fisher Scientific	#A32740	**AB_2762824**
Goat anti-rat Alexa Fluor-488	Thermo Fisher Scientific	#A11006	**AB_2534074**
Goat anti-mouse Alexa Fluor-488	Thermo Fisher Scientific	#A11029	**AB_2534088**

293T/17 [HEK 293T/17]

CRL-11268™

Description

293T/17 [HEK 293T/17] is a cell line exhibiting epithelial morphology that was isolated from human embryo kidney tissue. The cell line is a derivative of the 293T (293tsA1609neo) cell line, a highly transfectable derivative of the 293 cell line into which the temperature sensitive gene for SV40 T-antigen was inserted. 293T cells were cloned and the clones tested with the pBND and pZAP vectors to obtain a line capable of producing high titers of infectious retrovirus, 293T/17. These cells constitutively express the simian virus 40 (SV40) large T antigen, and clone 17 was selected specifically for its high transfectability.

Organism: *Homo sapiens*, human

Tissue: kidney

Age: fetus

Morphology: epithelial

Growth properties: Adherent

Patent depository: This material was deposited with the ATCC Patent Depository to fulfill U.S. or international patent requirements. This material may not have been produced or characterized by ATCC. As an International Depository Authority (IDA) for patent deposits, ATCC is required to complete viability testing only at time of initial deposit of patent material. Patent deposits are made available on behalf of the Depositor when the pertinent U.S. or international patent is issued, but material may not be used to infringe the patent claims.

Patent number:

6,329,199

Technical information: ATCC Technical Services does not have technical information on patent deposits that are not produced or characterized by ATCC. Additional information can be found in the corresponding patent available from the patent holder or with the U.S. and/or international patent office.

Storage Conditions

Product format: Frozen
Storage conditions: Vapor phase of liquid nitrogen

Intended Use

This product is intended for laboratory research use only. It is not intended for any animal or human therapeutic use, any human or animal consumption, or any diagnostic use.

BSL 2

ATCC determines the biosafety level of a material based on our risk assessment as guided by the current edition of *Biosafety in Microbiological and Biomedical Laboratories (BMBL)*, U.S. Department of Health and Human Services. It is your responsibility to understand the hazards associated with the material per your organization's policies and procedures as well as any other applicable regulations as enforced by your local or national agencies.

Cells contain Adenovirus DNA sequences

Cells contain SV40 sequences

ATCC highly recommends that appropriate personal protective equipment is always used when handling vials. For cultures that require storage in liquid nitrogen, it is important to note that some vials may leak when submersed in liquid nitrogen and will slowly fill with liquid nitrogen. Upon thawing, the conversion of the liquid nitrogen back to its gas phase may result in the vial exploding or blowing off its cap with dangerous force creating flying debris. Unless necessary, ATCC recommends that these cultures be stored in the vapor phase of liquid nitrogen rather than submersed in liquid nitrogen.

Certificate of Analysis

For batch-specific test results, refer to the applicable certificate of analysis that can be found at www.atcc.org.

Growth Conditions

Temperature: 37°C
Atmosphere: 95% Air, 5% CO_2

Handling Procedures

Unpacking and storage instructions:

1. Check all containers for leakage or breakage.
2. Remove the frozen cells from the dry ice packaging and immediately place the cells at a temperature below -130°C, preferably in liquid nitrogen vapor, until ready for use.

Complete medium: The base medium for this cell line is ATCC-formulated Dulbecco's Modified Eagle's Medium, Catalog No. 30-2002. To make the complete growth medium, add the following components to the base medium: fetal bovine serum to a final concentration of 10%.
Handling Procedure:

To insure the highest level of viability, thaw the vial and initiate the culture as soon as possible upon receipt. If upon arrival, continued storage of the frozen culture is necessary, it should be stored in liquid nitrogen vapor phase and not at -70°C. Storage at -70°C will result in loss of viability.

1. Thaw the vial by gentle agitation in a 37°C water bath. To reduce the possibility of contamination, keep the O-ring and cap out of the water. Thawing should be rapid (approximately 2 minutes).
2. Remove the vial from the water bath as soon as the contents are thawed, and decontaminate by dipping in or spraying with 70% ethanol. All of the operations from this point on should be carried out under strict aseptic conditions.
3. Transfer the vial contents to a centrifuge tube containing 9.0 mL complete

culture medium and spin at approximately 125 x *g* for 5 to 10 minutes.

4. Resuspend the cell pellet with the recommended complete medium (see the specific batch information for the culture recommended dilution ratio) and dispense into a 25 cm^2 or a 75 cm^2 culture flask. It is important to avoid excessive alkalinity of the medium during recovery of the cells. It is suggested that, prior to the addition of the vial contents, the culture vessel containing the complete growth medium be placed into the incubator for at least 15 minutes to allow the medium to reach its normal pH (7.0 to 7.6).
5. Incubate the culture at 37°C in a suitable incubator. A 5% CO_2 in air atmosphere is recommended if using the medium described on this product.

Subculturing procedure:
Volumes are given for a 75 cm^2 flask. Increase or decrease the amount of dissociation medium needed proportionally for culture vessels of other sizes. Corning T-75 flasks (catalog #430641) are recommended for subculturing this product.

1. Remove and discard culture medium.
2. Briefly rinse the cell layer with 0.25% (w/v) Trypsin- 0.53 mM EDTA solution to remove all traces of serum that contains trypsin inhibitor.
3. Add 2.0 to 3.0 mL of Trypsin-EDTA solution to flask and observe cells under an inverted microscope until cell layer is dispersed (usually within 5 to 15 minutes).
   Note: To avoid clumping do not agitate the cells by hitting or shaking the flask while waiting for the cells to detach. Cells that are difficult to detach may be placed at 37°C to facilitate dispersal.
4. Add 6.0 to 8.0 mL of complete growth medium and aspirate cells by gently pipetting.
5. Add appropriate aliquots of the cell suspension to new culture vessels.
6. Incubate cultures at 37°C.

Subcultivation Ratio: A subcultivation ratio of 1:4 to 1:8 is recommended
Medium Renewal: Every 2 to 3 days
Reagents for cryopreservation: Complete growth medium supplemented with 5% (v/v) DMSO (ATCC 4-X)

Material Citation

If use of this material results in a scientific publication, please cite the material in the

following manner: 293T/17 [HEK 293T/17] (ATCC CRL-11268)

References

References and other information relating to this material are available at www.atcc.org.

Warranty

The product is provided 'AS IS' and the viability of ATCC® products is warranted for 30 days from the date of shipment, provided that the customer has stored and handled the product according to the information included on the product information sheet, website, and Certificate of Analysis. For living cultures, ATCC lists the media formulation and reagents that have been found to be effective for the product. While other unspecified media and reagents may also produce satisfactory results, a change in the ATCC and/or depositor-recommended protocols may affect the recovery, growth, and/or function of the product. If an alternative medium formulation or reagent is used, the ATCC warranty for viability is no longer valid. Except as expressly set forth herein, no other warranties of any kind are provided, express or implied, including, but not limited to, any implied warranties of merchantability, fitness for a particular purpose, manufacture according to cGMP standards, typicality, safety, accuracy, and/or noninfringement.

Disclaimers

This product is intended for laboratory research use only. It is not intended for any animal or human therapeutic use, any human or animal consumption, or any diagnostic use. Any proposed commercial use is prohibited without a license from ATCC.

While ATCC uses reasonable efforts to include accurate and up-to-date information

on this product sheet, ATCC makes no warranties or representations as to its accuracy. Citations from scientific literature and patents are provided for informational purposes only. ATCC does not warrant that such information has been confirmed to be accurate or complete and the customer bears the sole responsibility of confirming the accuracy and completeness of any such information.

This product is sent on the condition that the customer is responsible for and assumes all risk and responsibility in connection with the receipt, handling, storage, disposal, and use of the ATCC product including without limitation taking all appropriate safety and handling precautions to minimize health or environmental risk. As a condition of receiving the material, the customer agrees that any activity undertaken with the ATCC product and any progeny or modifications will be conducted in compliance with all applicable laws, regulations, and guidelines. This product is provided 'AS IS' with no representations or warranties whatsoever except as expressly set forth herein and in no event shall ATCC, its parents, subsidiaries, directors, officers, agents, employees, assigns, successors, and affiliates be liable for indirect, special, incidental, or consequential damages of any kind in connection with or arising out of the customer's use of the product. While reasonable effort is made to ensure authenticity and reliability of materials on deposit, ATCC is not liable for damages arising from the misidentification or misrepresentation of such materials.

Please see the material transfer agreement (MTA) for further details regarding the use of this product. The MTA is available at www.atcc.org.

This material is cited in a US and/or international patent and may not be used to infringe the claims. Depending on the wishes of the Depositor, ATCC may be required to inform the Depositor of the party to which the material was furnished.

Copyright and Trademark Information

© ATCC 2023. All rights reserved.
ATCC is a registered trademark of the American Type Culture Collection.

Revision

This information on this document was last updated on 2024-10-25

Contact Information

ATCC
10801 University Boulevard
Manassas, VA 20110-2209
USA
US telephone: 800-638-6597
Worldwide telephone: +1-703-365-2700
Email: tech@atcc.org or contact your local distributor

CERTIFICATE OF ANALYSIS

ATCC® Number: CRL-11268™

Lot Number: 70022180

Name: 293T/17

Description: Embryonic Kidney

Species: Human (*Homo sapiens*)

Volume/Ampule: Approximately 1 mL

Date Frozen: 16JAN2019

Recovery: A T-75 setup at a dilution of 1:15 reaches approximately 20% confluence in 1 day and 100% in 5 days.

Product Format: Cells cryopreserved in the appropriate cryopreservation medium

Expiration Date: Not applicable

Storage Conditions: Vapor phase of liquid nitrogen

Test / Method	Specification	Result
**Ampule passage number**	Report results	17
**Population doubling level (PDL)**	Report results	Not applicable
**Total cells/ampule**<br>(Cell count using Trypan Blue stain method)	Report results	2.4×10^6 total cells/ampule
**Post-freeze viability**<br>(Cell count using Trypan Blue stain method)	≥ 50.0%	93.2%
**Growth properties**<br>(Visual observation method)	Adherent	Adherent
**Morphology**<br>(Visual observation method)	Epithelial-like*	Epithelial-like
**Test for mycoplasma contamination**<br>Hoechst DNA stain (indirect) method<br>Agar culture (direct) method<br>PCR-based assay	 None detected<br>None detected<br>None detected	 None detected<br>None detected<br>None detected
**Species determination: COI assay (interspecies)**	Human	Human

ATCC
10801 University Boulevard
Manassas, VA 20110-2209 USA
www.atcc.org

800-638-6597 or 703-365-2700
Fax: 703-365-2750
E-mail: tech@atcc.org
or contact your local distributor

CERTIFICATE OF ANALYSIS

ATCC® Number: **CRL-11268™**

Lot Number: **70022180**

**Species determination: STR analysis (intraspecies)**	**Human (Unique DNA Profile)**<br>TH01: 7, 9.3<br>D5S818: 8, 9<br>D13S317: 12, 14<br>D7S820: 11<br>D16S539: 9, 13<br>CSF1PO: 11, 12<br>Amelogenin: X<br>vWA: 16, 18, 19<br>TPOX: 11	**Human (Unique DNA Profile)**<br>TH01: 7, 9.3<br>D5S818: 8, 9<br>D13S317: 12, 14<br>D7S820: 11<br>D16S539: 9, 13<br>CSF1PO: 11, 12<br>Amelogenin: X<br>vWA: 16, 19 **<br>TPOX: 11
**Sterility test (BacT/ALERT 3D)**<br>iAST bottle (aerobic) at 32.5°C<br>iNST bottle (anaerobic) at 32.5°C	No growth<br>No growth	No growth<br>No growth
**Human pathogenic virus testing**<br>(PCR-based assay for HIV, HepB, HPV, EBV, and CMV)	Report results	HIV – None detected<br>HepB – None detected<br>HPV – None detected<br>EBV – None detected<br>CMV – None detected

* Epithelial-like: Any adherent cells of a polygonal shape with clear, sharp boundaries between them.
** This cell line has historically exhibited instability at vWA # 18. This particular lot exhibits loss of heterozygosity (LOH) at vWA #18.

Robbin L Smith

Digitally signed by Robbin L Smith
DN: cn=Robbin L Smith, o=ATCC, ou=Quality Assurance Specialist, email=rsmith@atcc.org, c=US
Date: 2019.03.04 15:39:11 -05'00'

Quality Assurance Specialist; Quality Assurance

ATCC hereby represents and warrants that the material provided under this certificate is pure and has been subjected to the tests and procedures specified and that the results described, along with any other data provided in this certificate, are true and correct to the best of the company's knowledge and belief. This certificate does not extend to the growth and/or passage of any living organism or cell line beyond what is supplied within the container received from ATCC.

This product is intended to be used for laboratory research use only. It is not intended for use in humans, animals, or for diagnostics. Appropriate Biosafety Level (BSL) practices should always be used with this material. Refer to the Product Information Sheet for instructions on the correct use of this product.

ATCC products may not be resold, modified for resale, used to provide commercial services, or to manufacture commercial products without prior written agreement from ATCC.

© 2017 ATCC. The ATCC trademark and trade name are owned by the American Type Culture Collection.

ATCC
10801 University Boulevard
Manassas, VA 20110-2209 USA
www.atcc.org

800-638-6597 or 703-365-2700
Fax: 703-365-2750
E-mail: tech@atcc.org
or contact your local distributor

Ceruloplasmin Recombinant Rabbit Monoclonal Antibody (6C3K9)

Product Details	
Size	100 µL
Species Reactivity	Human, Mouse, Rat
Host/Isotype	Rabbit / IgG
Expression system	HEK293 cells
Class	Recombinant Monoclonal
Type	Antibody
Clone	6C3K9
Conjugate	Unconjugated
Immunogen	Recombinant fusion protein containing a sequence corresponding to amino acids 366-572 of human Ceruloplasmin (NP_0000871)
Form	Liquid
Concentration	1 mg/mL
Purification	Affinity chromatography
Storage buffer	PBS, pH 7.3, with 50% glycerol, 0.05% BSA
Contains	0.05% ProClin 300
Storage conditions	-20° C, Avoid Freeze/Thaw Cycles
RRID	AB_2897953

Applications	Tested Dilution	Publications
Western Blot (WB)	1:1000-1:5,000	-
Immunohistochemistry (Paraffin) (IHC (P))	1:50-1:200	-
Immunocytochemistry (ICC/IF)	1:50-1:200	-

Product Specific Information

Positive Samples: Mouse serum, Rat testis, Rat serum

Immunogen sequence: IRGKHVRHYY IAAEEIIWNY APSGIDIFTK ENLTAPGSDS AVFFEQGTTR IGGSYKKLVY REYTDASFTN RKERGPEEEH LGILGPVIWA EVGDTIRVTF HNKGAYPLSI EPIGVRFNKN NEGTYYSPNY NPQSRSVPPS ASHVAPTETF TYEWTVPKEV GPTNADPVCL AKMYYSAVDP TKDIFTGLIG PMKICKKGSL HANGRQK

Product Images For Ceruloplasmin Recombinant Rabbit Monoclonal Antibody (6C3K9)

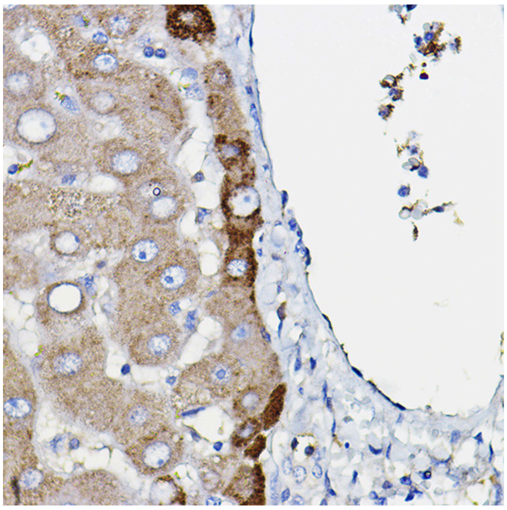

Ceruloplasmin Antibody (MA5-38035) in IHC (P)
Immunohistochemistry analysis of Ceruloplasmin in paraffin-embedded human liver. Samples were incubated with Ceruloplasmin Monoclonal antibody (Product # MA5-38035) using a dilution of 1:100 (40x lens). Perform high pressure antigen retrieval with 10 mM citrate buffer pH 6.0 before commencing with IHC staining protocol.

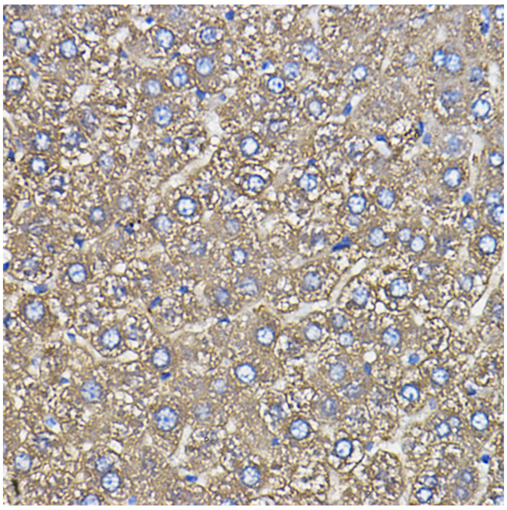

Ceruloplasmin Antibody (MA5-38035) in IHC (P)
Immunohistochemistry analysis of Ceruloplasmin in paraffin-embedded rat liver. Samples were incubated with Ceruloplasmin Monoclonal antibody (Product # MA5-38035) using a dilution of 1:100 (40x lens). Perform high pressure antigen retrieval with 10 mM citrate buffer pH 6.0 before commencing with IHC staining protocol.

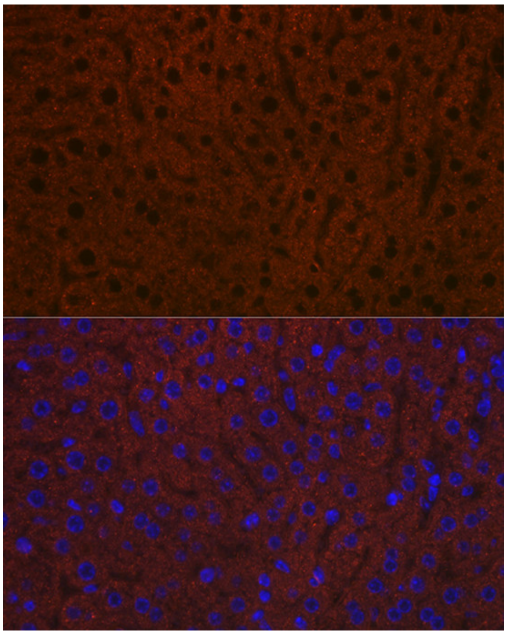

Ceruloplasmin Antibody (MA5-38035) in IHC (P)
Immunohistochemistry (Immunofluorescence) analysis of Ceruloplasmin in mouse liver. Samples were incubated with Ceruloplasmin Monoclonal antibody (Product # MA5-38035) using a dilution of 1:100 (40x lens). Blue: DAPI for nuclear staining.

View more figures on thermofisher.com

For Research Use Only. Not for use in diagnostic procedures. Not for resale without express authorization. Products are warranted to operate or perform substantially in conformance with published Product specifications in effect at the time of sale, as set forth in the Production documentation, specifications and/or accompanying package inserts ("Documentation"). No claim of suitability for use in applications regulated by FDA is made. The warranty provided herein is valid only when used by properly trained individuals. Unless otherwise stated in the Documentation, this warranty is limited to one year from date of shipment when the Product is subjected to normal, proper and intended usage. This warranty does not extend to anyone other than the Buyer. Any model or sample furnished to Buyer is merely illustrative of the general type and quality of goods and does not represent that any Product will conform to such model or sample. NO OTHER WARRANTIES, EXPRESS OR IMPLED, ARE GRANTED INCLUDING WITHOUT LIMITATION, IMPLIED WARRANTIES OF MERCHANTABILITY, FITNESS FOR ANY PARTICULAR PURPOSE, OR NON INFRINGEMENT. BUYER'S EXCLUSIVE REMEDY FOR NON-CONFORMING PRODUCTS DURING THE WARRANTY PERIOD IS LIMITED TO REPAIR, REPLACEMENT OF OR REFUND FOR THE NON-CONFORMING PRODUCT(S) AT SELLER'S SOLE OPTION. THERE IS NO OBLIGATION TO REPAIR, REPLACE OR REFUND FOR PRODUCTS AS THE RESULT OF (I) ACCIDENT, DISASTER OR EVENT OF FORCE MAJEURE, (II) MISUSE, FAULT OR NEGLIGENCE OF OR BY BUYER, (III) USE OF THE PRODUCTS IN A MANNER FOR WHICH THEY WERE NOT DESIGNED, OR (IV) IMPROPER STORAGE AND HANDLING OF THE PRODUCTS. Unless otherwise expressly stated on the Product or in the documentation accompanying the Product, the Product is intended for research only and is not to be used for any other purpose, including without limitation, unauthorized commercial uses, in vitro diagnostic uses, ex vivo or in vivo therapeutic uses, or any type of consumption by or application to human or animals.

Ceruloplasmin Polyclonal Antibody

**Product Details**	
Size	100 µg
Species Reactivity	Human
Host/Isotype	Rabbit / IgG
Class	Polyclonal
Type	Antibody
Conjugate	Unconjugated
Immunogen	E.coli-derived human Ceruloplasmin recombinant protein (Position: K20-M259).
Form	Lyophilized
Concentration	500 µg/mL
Purification	Affinity chromatography
Storage buffer	PBS with 5mg BSA
Contains	0.05mg sodium azide
Storage conditions	Store at 4°C short term. For long term storage, store at -20°C, avoiding freeze/thaw cycles.
RRID	AB_2807139

**Applications**	**Tested Dilution**	**Publications**
Western Blot (WB)	0.1-0.5 µg/mL	-
Immunohistochemistry (Paraffin) (IHC (P))	0.5-1 µg/mL	-

Product Specific Information

Human Ceruloplasmin shares 80.8% and 79.6% amino acid (aa) sequence identity with mouse and rat Ceruloplasmin, respectively.

Reconstitute with 0.2 mL of distilled water to yield a concentration of 500 µg/mL.

Product Images For Ceruloplasmin Polyclonal Antibody

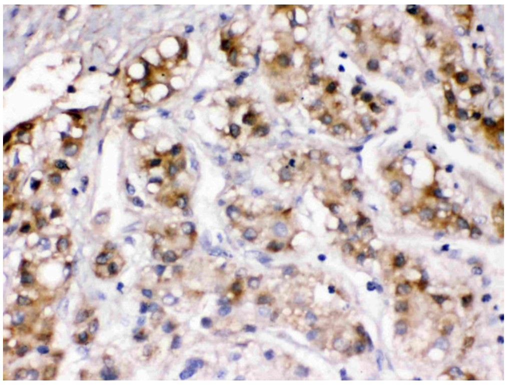

Ceruloplasmin Antibody (PA5-95336) in IHC (P)
Immunohistochemistry analysis of Ceruloplasmin in paraffin-embedded human liver cancer tissues. Samples were incubated with Ceruloplasmin polyclonal antibody (Product # PA5-95336) at a 1 µg/mL dilution, and developed with Strepavidin-Biotin-Complex.

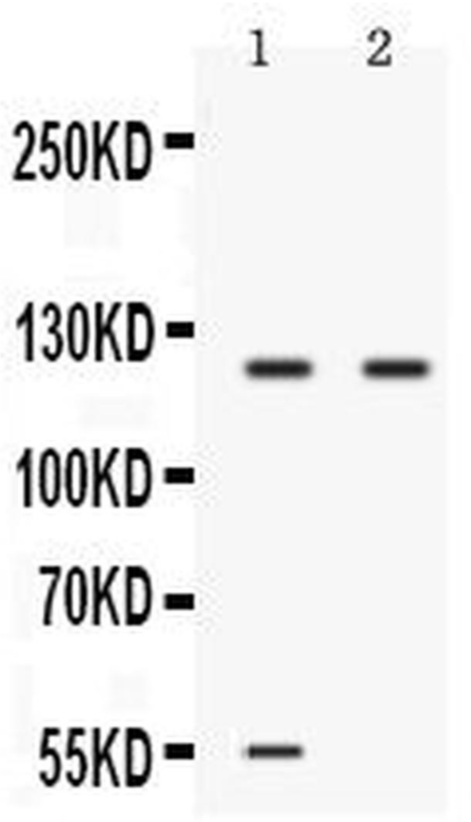

Ceruloplasmin Antibody (PA5-95336) in WB
Western blot analysis of Ceruloplasmin in 22RV1 whole cell lysates (lane 1) and A549 whole cell lysates (lane 2). Samples were incubated with Ceruloplasmin polyclonal antibody (Product # PA5-95336) using a 0.5 µg/mL dilution. developed was performed using enhanced chemiluminescence (ECL).

For Research Use Only. Not for use in diagnostic procedures. Not for resale without express authorization. Products are warranted to operate or perform substantially in conformance with published Product specifications in effect at the time of sale, as set forth in the Production documentation, specifications and/or accompanying package inserts ("Documentation"). No claim of suitability for use in applications regulated by FDA is made. The warranty provided herein is valid only when used by properly trained individuals. Unless otherwise stated in the Documentation, this warranty is limited to one year from date of shipment when the Product is subjected to normal, proper and intended usage. This warranty does not extend to anyone other than the Buyer. Any model or sample furnished to Buyer is merely illustrative of the general type and quality of goods and does not represent that any Product will conform to such model or sample. NO OTHER WARRANTIES, EXPRESS OR IMPLED, ARE GRANTED INCLUDING WITHOUT LIMITATION, IMPLIED WARRANTIES OF MERCHANTABILITY, FITNESS FOR ANY PARTICULAR PURPOSE, OR NON INFRINGEMENT. BUYER'S EXCLUSIVE REMEDY FOR NON-CONFORMING PRODUCTS DURING THE WARRANTY PERIOD IS LIMITED TO REPAIR, REPLACEMENT OF OR REFUND FOR THE NON-CONFORMING PRODUCT(S) AT SELLER'S SOLE OPTION. THERE IS NO OBLIGATION TO REPAIR, REPLACE OR REFUND FOR PRODUCTS AS THE RESULT OF (I) ACCIDENT, DISASTER OR EVENT OF FORCE MAJEURE, (II) MISUSE, FAULT OR NEGLIGENCE OF OR BY BUYER, (III) USE OF THE PRODUCTS IN A MANNER FOR WHICH THEY WERE NOT DESIGNED, OR (IV) IMPROPER STORAGE AND HANDLING OF THE PRODUCTS. Unless otherwise expressly stated on the Product or in the documentation accompanying the Product, the Product is intended for research only and is not to be used for any other purpose, including without limitation, unauthorized commercial uses, in vitro diagnostic uses, ex vivo or in vivo therapeutic uses, or any type of consumption by or application to human or animals.

abcam

ab48614

Anti-Ceruloplasmin antibody

Rabbit Polyclonal Ceruloplasmin antibody. Suitable for IHC-P, IP, ELISA, WB, IHC-FoFr, RIA, ICC/IF, EIA and reacts with Human samples. Cited in 15 publications. Immunogen corresponding to Native Full Length Protein corresponding to Human CP.

Alternative names

Ceruloplasmin, Ferroxidase, CP

Key facts

**Isotype**	IgG
**Host species**	Rabbit
**Storage buffer**	pH: 7.4<br>Preservative: 0.02% Sodium azide<br>Constituents: 50% Glycerol (glycerin, glycerine), 49.98% PBS
**Form**	Liquid
**Clonality**	Polyclonal
**Immunogen**	Native Full Length Protein corresponding to Human CP. Database link P00450
**Purification technique**	Affinity purification Protein G
**Concentration**	0.38 - 1.014 mg/mL The concentration of this product may be batch-dependent<br>Batch concentration finder →

Reactivity data

IHC-P

Tested

**Species**	Human
**Dilution info**	2 µg/mL

**Notes**	Perform heat-mediated antigen retrieval before commencing with IHC staining protocol.

IP

Expected

**Species**	Human
**Dilution info**	Use at an assay dependent concentration.
**Notes**	-

ELISA

Expected

**Species**	Human
**Dilution info**	Use at an assay dependent concentration.
**Notes**	-

WB

Tested

**Species**	Human
**Dilution info**	-
**Notes**	-

IHC-FoFr

Expected

**Species**	Human
**Dilution info**	Use at an assay dependent concentration.
**Notes**	-

RIA

Expected

**Species**	Human
**Dilution info**	Use at an assay dependent concentration.
**Notes**	-

ICC/IF

Expected

**Species**	Human
**Dilution info**	Use at an assay dependent concentration.
**Notes**	-

EIA

Expected

**Species**	Human
**Dilution info**	Use at an assay dependent concentration.
**Notes**	-

Storage

**Shipped at conditions**	Blue Ice
**Appropriate long-term storage conditions**	-20°C
**Aliquoting information**	Upon delivery aliquot
**Storage information**	Avoid freeze / thaw cycle

Notes

Abcam is leading the way to address reproducibility in scientific research with our highly validated recombinant monoclonal and recombinant multiclonal antibodies. Search & select one of Abcam's thousands of recombinant alternatives to eliminate batch-variability and unnecessary animal use.

If you do not find a host species to meet your needs, our catalogue and custom Chimeric range provides scientists the specificity of Abcam's RabMAbs in the species backbone of your choice. Remember to also review our range of edited cell lines, proteins and biochemicals relevant to your target that may help you further your research goals.

Abcam antibodies are extensively validated in a wide range of species and applications, so please check the reagent specifications meet your scientific needs before purchasing. If you have any questions or bespoke requirements, simply visit the Contact Us page to send us an inquiry or contact our Support Team ahead of purchase.

Product promise

Tested

We have tested this species and application combination and it works. It is covered by our product promise.

Expected

We have not tested this specific species and application combination in-house, but expect it will work. It is covered by our product promise.

Predicted

This species and application combination has not been tested, but we predict it will work based on strong homology. However, this combination is not covered by our product promise.

Not recommended

We do not recommend this combination. It is not covered by our product promise.

We are dedicated to supporting your work with high quality reagents and we are here for you every step of the way should you need us.

In the unlikely event of one of our products not working as expected, you are covered by our product promise.

Full details and terms and conditions can be found here:
Terms & Conditions.

2 product images

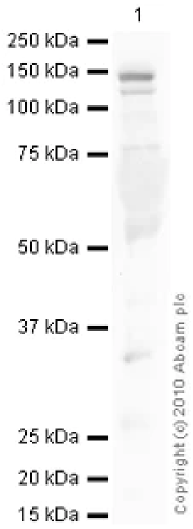

Western blot - Anti-Ceruloplasmin antibody (ab48614)

Ceruloplasmin contains a number of potential glycosylation sites (SwissProt) which may explain its migration at a higher molecular weight than predicted (148 kDa).

All lanes:
Western blot - Anti-Ceruloplasmin antibody (AB48614) at 1 µg/mL

All lanes:
Human Plasma Total Protein Lysate at 10 µg

Secondary

All lanes:
Western blot - Goat Anti-Rabbit IgG H&L (HRP) preadsorbed (AB97080) at 1/5000 dilution

Developed using the ECL technique.

Performed under reducing conditions.

Predicted band size: 122 kDa

Observed band size: 122 kDa, 148 kDa, 34 kDa, 76 kDa

Exposure time: 30s

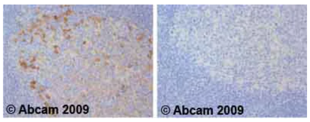

Immunohistochemistry (Formalin/PFA-fixed paraffin-embedded sections) - Anti-Ceruloplasmin antibody (ab48614)

Ab48614 staining human tonsil. Staining is localized to the cytoplasm.
Left panel: with primary antibody at 2 ug/ml. Right panel: isotype control.
Sections were stained using an automated system (Dako PT Link), at room temperature. Sections were rehydrated and antigen retrieved with the Dako 3-in-1 antigen retrieval buffer, EDTA pH 9.0. Slides were peroxidase blocked in 3% H_2O_2 in methanol for 10 minutes. They were then blocked with Dako Protein block for 10 minutes (containing casein 0.25% in PBS) then incubated with primary antibody for 20 minutes and detected with Dako Envision Flex amplification kit for 30 minutes. Colorimetric detection was completed with diaminobenzidine for 5 minutes. Slides were counterstained with Haematoxylin and coverslipped under DePeX. Please note that for manual staining we recommend to optimize the primary antibody concentration and incubation time (overnight incubation), and amplification may be required.

Please note: All products are 'FOR RESEARCH USE ONLY. NOT FOR USE IN DIAGNOSTIC OR THERAPEUTIC PROCEDURES'.

Rabbit anti-Sheep IgG (H+L) Secondary Antibody, HRP

Product Details	
Size	1.5 mL
Species Reactivity	Sheep
Host/Isotype	Rabbit / IgG
Class	Polyclonal
Type	Secondary Antibody
Conjugate	HRP
Form	Lyophilized
Concentration	0.8 mg/mL
Purification	Affinity chromatography
Storage buffer	PBS, pH 7.6, with 15mg/mL BSA
Contains	no preservative
Storage conditions	4° C
RRID	AB_228457

Applications	Tested Dilution	Publications
Western Blot (WB)	1:2000-1:20,000	0 Publication
Immunohistochemistry (IHC)	1:500-1:5,000	0 Publication
Immunocytochemistry (ICC/IF)	1:500-1:5,000	-
ELISA (ELISA)	-	0 Publication

Product Specific Information

Concentration may vary slightly from lot-to-lot, see lot-specific datasheet for exact concentration.

This antibody has been successfully used in Western blot, and ICC applications.

Antibody Specificity: The antibody reacts with the heavy chains of sheep IgG and with light chains common to most sheep immunoglobulins, based on immunoelectrophoresis. No antibody was detected against non-immunoglobulin serum proteins. However, this antibody may cross-react with immunoglobulins from other species.

Restoration and Storage: Store product at 4°C until opened. Restore with 1.5 mL distilled water (0.8 mg/mL after restoration). Centrifuge product if it is not completely clear after standing for 1-2 hours at room temperature. To judge clarity, draw product into a pasteur pipette. Product may be stored for several weeks at 4°C as an undiluted liquid. After dilution, do not use for more than one day.

To extend the shelf-life of this product, add an equal volume of glycerol to make a final concentration of approximately 50% glycerol and store at -20°C.

Country of Origin: USA

Product Images For Rabbit anti-Sheep IgG (H+L) Secondary Antibody, HRP

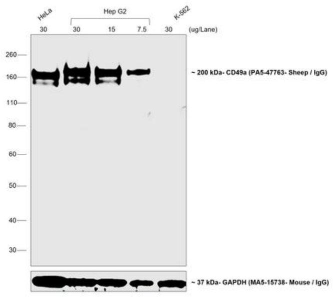

Sheep IgG (H+L) Secondary Antibody (31480) in WB

Chemiluminescent western blot was performed using Rabbit anti-Sheep IgG (H+L) Secondary Antibody, HRP (Product # 31480). Membrane enriched extracts of HeLa (Lane 1), Hep G2 (Lane 2, 3, 4) and K-562 (Lane 5) were electrophoresed usingNuPAGE™ 3 to 8%, Tris-Acetate, 1.0 mm, Mini Protein Gel, 10-well (Product # EC6695BOX). Resolved proteins were transferred onto anitrocellulose membrane (Product # IB23001) byiBlot® 2 Dry BlottingSystem (Product # IB21001). The blot was probed with CD49a Sheep Polyclonal Antibody (Product # PA5-47763). Secondary antibody (Product # 31480, 1: 30,000 dilution) was used for detection of CD49a by chemiluminescence with SuperSignal™ West Pico PLUS Chemiluminescent Substrate (Product # 34580) using the iBright FL 1500 (Product # A44115). The anti-sheep secondary antibody (Product # 31480) specifically detects the sheep primary antibody.

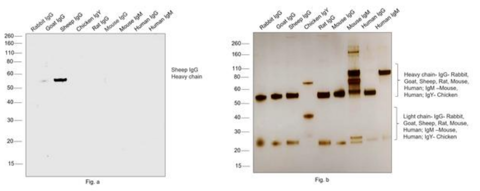

Sheep IgG (H+L) Secondary Antibody (31480) in WB

Western blot was performed using Rabbit anti-Sheep IgG (H+L) Secondary Antibody, HRP (Product # 31480) and ~50 kDa band corresponding to Sheep IgG Heavy Chain was observed in Sheep IgG but not in Rabbit IgG, Rat IgG, Chicken IgY, Mouse IgG, Mouse IgM, Human IgG and Human IgM. Purified protein (100 ng) of Rabbit IgG (Lane 1), Goat IgG (Lane 2), Sheep IgG (Lane 3), Chicken IgY (Lane 4), Rat IgG (Lane 5), Mouse IgG (Lane 6), Mouse IgM (Lane 7), Human IgG (Lane 8), Human IgM (Lane 9) (Fig. a) were electrophoresed using NuPAGE™ 4-12% Bis-Tris Protein Gel (Product # NP0321BOX). Resolved proteins were then transferred onto a nitrocellulose membrane (Product # IB23001) by iBlot® 2 Dry Blotting System (Product # IB21001). The blot was probed with Rabbit anti-Sheep IgG (H+L) Secondary Antibody, HRP (Product # 31480, 1:2000 dilution) and detected using the iBright FL1500 (Product # A44115). Silver staining was performed to establish equivalent loading of purified proteins using the Pierce™ Silver Stain Kit (Product # 24612) (Fig. b). The secondary antibody showed cross reactivity with Goat IgG.

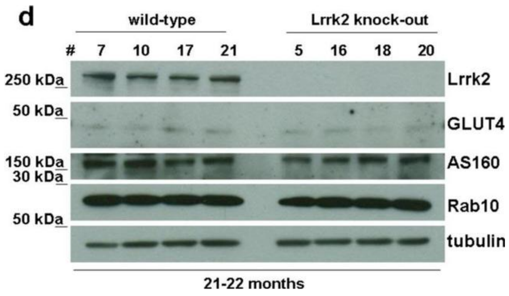

Sheep IgG (H+L) Secondary Antibody (31480) in WB

Insulin signalling in Lrrk2 deficient animals. (a) Insulin-triggered phosphorylation of IR and AS160 (Thr642) in fibroblasts from 22 months old Lrrk2 deficient rats at different time-points after stimulation and the corresponding quantification of P-IR (b, n = 7, normalized to IR-) and P-AS160 Thr642 (c, n = 10, normalized to AS160) signal intensity (mean ¬± SEM). (d) Western blot analysis (fibroblasts from 22 months old rats as example) and quantification of total GLUT4, AS160 and Rab10 expression in fibroblasts from 6 months (e) and 22 months old (f) Lrrk2 deficient and wild-type rats (normalized to tubulin, mean ¬± SEM). # are numbers of animals/cell lines. The difference in GLUT4 and AS160 signal intensity between 6 months und 22 months old sample-groups results from differences in experimental procedure and does not reflect the absolute quantity of GLUT4 or rather AS160 in these age groups. (g) Investigation of Rab10 phosphorylation by Mn2+ Phos-tag SDS-PAGE in fibroblasts from 6 months old Lrrk2 deficient and wild-type rats at different time points (0-10-30-40 min) after insulin addition and (h) corresponding quantification of P-Rab10 signal intensity in wild-type cells at different time points after stimulation (normalized to Rab10, n = 7, mean and SEM). Image collected and cropped by CiteAb from the following publication (https://pubmed.ncbi.nlm.nih.gov/30872638), licensed under a CC BY license.

View more figures on thermofisher.com

62 References

Targeted dephosphorylation of SMAD3 as an approach to impede TGF- signaling. iScience (2024)

Targeted dephosphorylation of TFEB promotes its nuclear translocation. iScience (2024)

PPAR activation by lipolysis-generated ligands is required for cAMP dependentUCP1induction in human thermogenic adipocytes bioRxiv (2024)

CK2 phosphorylation of CMTR1 promotes RNA cap formation and influenza virus infection. Cell Rep (2024)

A novel FAM83G variant from palmoplantar keratoderma patient disrupts WNT signalling via loss of FAM83G-CK1 interaction. Open Biol (2024)

For Research Use Only. Not for use in diagnostic procedures. Not for resale without express authorization. Products are warranted to operate or perform substantially in conformance with published Product specifications in effect at the time of sale, as set forth in the Production documentation, specifications and/or accompanying package inserts ("Documentation"). No claim of suitability for use in applications regulated by FDA is made. The warranty provided herein is valid only when used by properly trained individuals. Unless otherwise stated in the Documentation, this warranty is limited to one year from date of shipment when the Product is subjected to normal, proper and intended usage. This warranty does not extend to anyone other than the Buyer. Any model or sample furnished to Buyer is merely illustrative of the general type and quality of goods and does not represent that any Product will conform to such model or sample. NO OTHER WARRANTIES, EXPRESS OR IMPLED, ARE GRANTED INCLUDING WITHOUT LIMITATION, IMPLIED WARRANTIES OF MERCHANTABILITY, FITNESS FOR ANY PARTICULAR PURPOSE, OR NON INFRINGEMENT. BUYER'S EXCLUSIVE REMEDY FOR NON-CONFORMING PRODUCTS DURING THE WARRANTY PERIOD IS LIMITED TO REPAIR, REPLACEMENT OF OR REFUND FOR THE NON-CONFORMING PRODUCT(S) AT SELLER'S SOLE OPTION. THERE IS NO OBLIGATION TO REPAIR, REPLACE OR REFUND FOR PRODUCTS AS THE RESULT OF (I) ACCIDENT, DISASTER OR EVENT OF FORCE MAJEURE, (II) MISUSE, FAULT OR NEGLIGENCE OF OR BY BUYER, (III) USE OF THE PRODUCTS IN A MANNER FOR WHICH THEY WERE NOT DESIGNED, OR (IV) IMPROPER STORAGE AND HANDLING OF THE PRODUCTS. Unless otherwise expressly stated on the Product or in the documentation accompanying the Product, the Product is intended for research only and is not to be used for any other purpose, including without limitation, unauthorized commercial uses, in vitro diagnostic uses, ex vivo or in vivo therapeutic uses, or any type of consumption by or application to human or animals.

Amersham

ECL Anti-rabbit IgG, Horseradish Peroxidase- Linked Species-Specific $F(ab')_2$ Fragment (from donkey)

Product Specification Sheet

Introduction

Product code

NA9340

Important

Read these instructions carefully before using the products.

Intended use

The products are intended for research use only, and shall not be used in any clinical or *in vitro* procedures for diagnostic purposes.

Safety

All chemicals should be considered as potentially hazardous. For use and handling of the products in a safe way, refer to the Safety Data Sheets.

Storage

Store at 2–8°C. Do not freeze. Under these conditions, the product is stable for at least 12 months from the date of despatch.

Expiry

See outer packaging.

Component

Horseradish Peroxidase $F(ab')_2$ fragments are supplied in Phosphate Buffered Saline (Sodium Phosphate 0.1 M, NaCl 0.1 M) pH 7.5, containing 1% (w/v) Bovine Serum Albumin and an anti-microbial agent.

Description

Purification to ensure species-specificity

The antibody is prepared by hyper-immunizing donkeys with purified immunoglobulin fractions from normal rabbit serum to produce high affinity antibodies. The pooled antiserum is used to produce an immunoglobulin preparation which is then affinity adsorbed to remove cross-reacting antibodies towards rat, human and mouse immunoglobulins. These activities are thoroughly depleted to ensure species-specificity.

Finally, to select for specific binding to rabbit IgG, the antibodies are purified using an affinity column of rabbit IgG. After washing to remove non-specific serum components and low affinity antibodies, the species-specific antibodies are eluted using carefully selected, mild conditions which minimize aggregation and preserve immunological activity, yet which will elute high affinity antibodies.

The $F(ab')_2$ fragments are produced by digestion of the whole antibodies with pepsin. Undigested IgG Fc fragments and pepsin are removed by gel filtration. The purity of the separated $F(ab')_2$ fragments is checked by gel filtration chromatography.

Preparation of labelled antibody

The enzyme Horseradish Peroxidase is attached to the $F(ab')_2$ fragments using an adaptation of the periodate oxidation technique (1). This method has been found not to affect the effective binding of the antibody to the antigen or the activity of the enzyme.

Quality control

For every batch of enzyme-linked antibody that is produced the antibody titre is determined in an ELISA. The substrate used for the peroxidase is 2,2′-Azinobis[3-Ethylbenzothiazoline Sulphonate, diammonium salt], ABTS™.

Every batch is also QC tested in a Western blotting system. This is performed using Hybond™ ECL™ membrane containing Beta-Galactosidase protein and immunodetected with primary antibody Anti-Beta Galactosidase (Cappel) and secondary antibody NA9340, anti-rabbit IgG, HRP $F(ab')_2$ fragment. Blots are detected using ECL and ECL Plus detection systems.

Applications

1. **Protein blotting**

   a. **Detection with ECL (2) Western blotting reagents**

   This reagent has been shown to be suitable for use in ECL Western blotting applications.

   The control system used was the detection of Anti-Beta Galactosidase.

   We have found in our laboratories that dilutions of 1:5000 for Anti-Beta Galactosidase and 1:50 000 for NA9340 are suitable for the detection of 6 ng of Beta-Galactosidase on Hybond ECL membrane, exposed to Hyperfilm™ ECL for 5 minutes.

To achieve the same sensitivity level on Hybond-P PVDF, dilutions would typically be Anti-Beta Galactosidase - 1:5000 and NA9340 - 1:100 000.

b. **Detection with ECL Plus(3,4) Western blotting reagents**

ECL Plus Western blotting reagent is highly sensitive, giving an increase, for this antibody, of 4–20 fold over ECL detection. This property can be utilized in 2 ways:

- Preservation of antibodies that are rare or costly
- Increase in detectable sensitivity levels

The control system used was the same as for ECL.

The suitable antibody dilutions, to detect 6 ng of Beta-Galactosidase on Hybond ECL membrane are Anti-Beta Galactosidase - 1:10 000 and NA9340 - 1:100 000.

For Hybond-P PVDF, dilutions would typically be Anti-Beta Galactosidase - 1:20 000 and NA9340 - 1:200 000.

c. **Colorimetric detection**

A dilution of 1:300 is recommended.

2. **ELISA**

If this reagent is to be used to detect mouse immunoglobulins, we have found in our laboratories that a dilution of 1:9000 is suitable for the detection of 1 µg of IgG. For greater sensitivity (for example down to 300 pg) the reagent should be diluted rather less (for example 1:5000). Thus 1.0 ml of stock reagent will be sufficient for up to 90 000 wells at the higher dilution if used at 0.1 ml per well in standard microplates. A suitable diluent is Phosphate-Buffered Saline containing 0.05% (v/v) Tween™ 20.

3. **Immunocytochemistry**

When using the reagent as a second antibody in immunocytochemistry on sections of formalin-fixed wax-embedded tissue the antibody can be typically diluted 1:100 in Phosphate-Buffered Saline. The user may wish to adjust this to obtain the required sensitivity for the tissue under investigation. Assuming that 0.1 ml of the diluted antibody can be used to cover the tissue section then 1.0 ml of stock reagent will be sufficient for up to 1000 slides. If frozen sections are used, acceptable staining may be obtained using even higher dilutions of the reagent.

Protocol recommendations

Membranes

Nitrocellulose and PVDF membranes are suitable for use with both detection systems. PVDF membrane is highly recommended for use with ECL Plus detection reagents.

For high quality results the following guidelines should be followed:

Blocking: Use enough blocking agent to block all non-specific sites. A typical block is 5% non-fat dried milk in PBS Tween or TBS Tween. See *'Tech-Tips' No. 136* available from Cytiva, for further details.

Washing: The volume of wash buffer (eg PBS-T or TBS-T) must be sufficient to cover the membrane completely.

Optimization of primary and secondary antibodies

ECL detection

ECL Western blotting is a very sensitive technique. As such it is essential to optimize the system under study for high signal and low background for both primary and secondary antibodies.

Dot blots are a quick and effective method of determining the optimum dilutions required for primary and secondary antibodies. Optimization details are set out in the *RPN2106/2108/2109/2209/2134 booklets* and *'Tech-Tips' No. 129* available from Cytiva.

ECL Plus detection

Due to the improved sensitivity of ECL Plus compared to ECL, optimization details as set out in the *RPN2132/2133 booklets* and *'Tech-Tips' No. 169* available from Cytiva recommended.

Typical anti-rabbit secondary antibody dilution ranges:

ECL for nitrocellulose membrane	1:5000 to 1:50 000
ECL Plus for nitrocellulose membrane	1:10 000 to 1:100 000

For PVDF membrane the use of higher dilutions may be necessary. The exact concentration of the secondary antibody will always be dependent upon the primary antibody used and the sensitivity and exposure times required.

Detection: Ensure any excess ECL or ECL Plus detection reagents are sufficiently drained prior to exposure.

Exposure times:

ECL - exposure times of 1 to 15 minutes are suggested.

ECL Plus - initial exposure times of 1 to 5 minutes are suggested.

Signal can still be obtained up to 24 hours after the application of ECL Plus reagents, and for this exposure times of 1 to 2 hours may be required.

Related products

ECL Western blotting detection reagents	RPN2106/2108/2109/2209/2134
ECL Plus Western blotting detection system	RPN2132/2133
Hybond ECL membrane	RPN2020D
Hybond-P PVDF membrane	RPN2020F
Hyperfilm ECL	RPN2103/2104/1681/1674
ECL protein molecular weight markers	RPN2107

References

1. NAKANE, P.K. and KAWAOI, A., *Journal of Histochemistry and Cytochemistry*, **22**, pp.1084-1091, 1974.
2. WHITEHEAD, T.P. *et al., Clin. Chem*., **25**, pp.1531-1546, 1979.
3. AKHAVEN-TAFTI, H. *et al., Clin. Chem*., **41**, pp.1368-1369, 1995.
4. AKHAVEN-TAFTI, H. *et al., Biolum. And Chemilum. Fundamentals and Applied Aspects*, pp.199-202, Chichester, 1994.

cytiva.com

Cytiva and the Drop logo are trademarks of Global Life Sciences IP Holdco LLC or an affiliate.

Amersham™, Hybond, Hyperfilm and ECL are trademarks of Global Life Sciences Solutions USA LLC or an affiliate doing business as Cytiva.

ABTS is a trademark of Boehringer Mannheim Corp.

Tween is a trademark of Croda Group of Companies

All other third-party trademarks are the property of their respective owners.

© 2020–2021 Cytiva

All goods and services are sold subject to the terms and conditions of sale of the supplying company operating within the Cytiva business. A copy of those terms and conditions is available on request. Contact your local Cytiva representative for the most current information.

For local office contact information, visit cytiva.com/contact

NA9340PS AF V:4 03/2021

Ceruloplasmin Antibody

ALTERNATE NAMES: Ferroxidase, CP, CP-2

CATALOG NO. : 7019-100

AMOUNT: 100 µg (0.5 mg/ml)

HOST: Rabbit

IMMUNOGEN: human ceruloplasmin (Cat No. 4096)

INTERNAL ID: BV-P34

PURIFICATION: Affinity purified rabbit IgG

MOLECULAR WEIGHT: ~132 kDa

FORM: Liquid

FORMULATION: Supplied in PBS (pH 7.2) with 0.01 % BSA, 0.03 % ProClin®, and 50 % glycerol

SPECIES REACTIVITY: Human, Mouse, Rat

STORAGE CONDITIONS: Store at -20°C. Avoid repeated freeze/thaw cycles.

DESCRIPTION: Ceruloplasmin is a blue, copper-binding (6-7 atoms per molecule) glycoprotein. It has ferroxidase activity oxidizing Fe2+ to Fe3+ without releasing radical oxygen species. It is involved in iron transport across the cell membrane. Provides Cu2+ ions for the ascorbate-mediated deaminase degradation of the heparan sulfate chains of GPC1. May also play a role in fetal lung development or pulmonary antioxidant defense

APPLICATION: Western blot: 1-4 µg/ml

Note: This information is only intended as a guide. The optimal dilutions must be determined by the user.

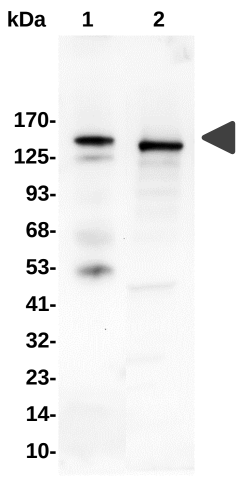

Western blot analysis of anti-Ceruloplasmin pAb in human serum and rat kidney. Ceruloplasmin (arrow) was detected using the purified antibody.

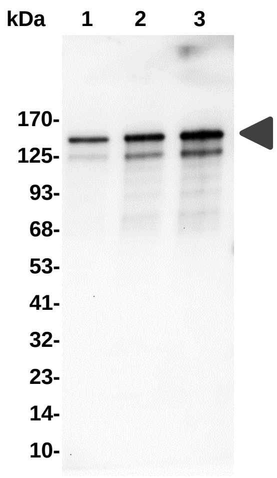

Dose-dependent western blot analysis of anti-Ceruloplasmin pAb using
Lane 1: 2 ng human ceruloplasmin protein
Lane 2: 5 ng human ceruloplasmin protein
Lane 3: 10 ng human ceruloplasmin protein

RELATED PRODUCTS:

- Ceruloplasmin, Human Plasma (Cat. No. 4096-1000)
- Ceruloplasmin Activity Colorimetric Assay Kit (Cat No. K669-100)

FOR RESEARCH USE ONLY! Not to be used on humans.

Grp94 monoclonal antibody (9G10)

This antibody is covered by our Worry-Free Guarantee.

Grp94 (Glucose-regulated protein 94) is an abundant resident endoplasmic reticulum (ER) lumenal stress protein, which together with cytosolic Hsp90 belongs to the Hsp90 family of molecular chaperones. Grp94 expression is upregulated by stress conditions such as glucose starvation and heat shock, which promote protein misfolding or unfolding. In addition to a homeostatic role in protein folding and assembly, Grp94 can function in the intracellular trafficking of peptides from the extracellular space to the MHC class I antigen processing pathway of antigen presentation cells.

Citations: 33 View Online »

Ordering Information

Order Online »

**ADI-SPA-850-D**	50µg
**ADI-SPA-850-F**	200µg

Manuals, SDS & CofA

View Online »

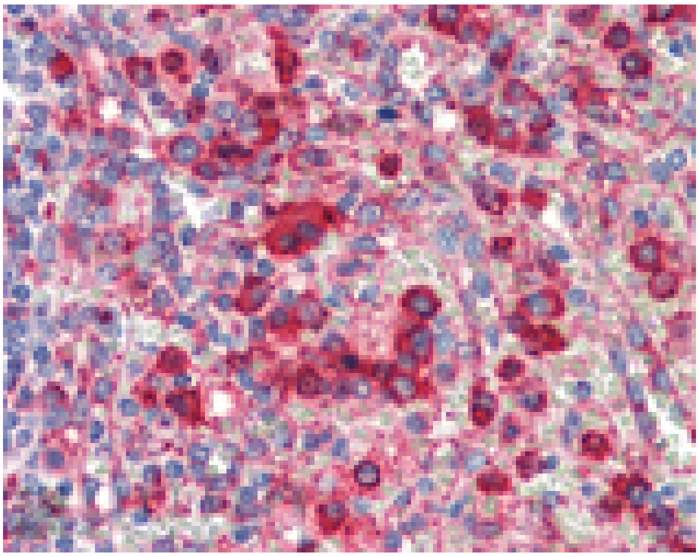<br>Immunohistochemistry analysis of human spleen tissue stained with Grp94, mAb (9G10) at 10µg/ml.	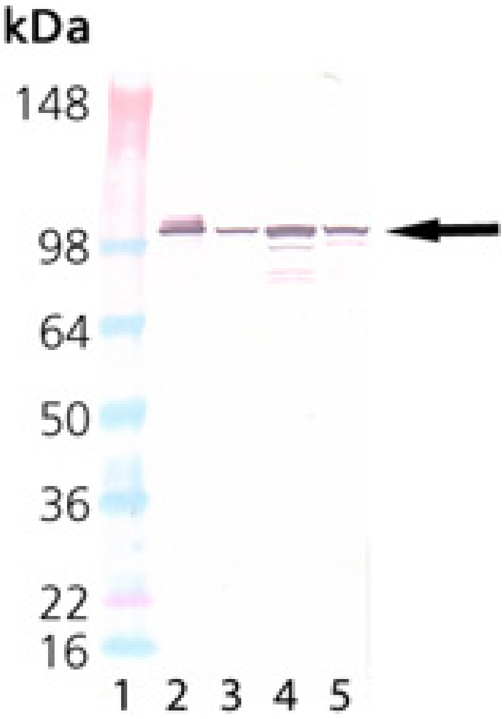<br>Western Blot Analysis of Grp94: Lane 1: MW Marker, Lane 2: Grp94 (canine), (recombinant) (Prod No. ADI-SPP-766), Lane 3: HeLa, (cell lysate) (Prod No. ADI-LYC-HL100), Lane 4: Mouse Liver Lysate, Lane 5: Vero Cell Lysate

Handling & Storage

**Handling**	Avoid freeze/thaw cycles.
**Long Term Storage**	-20°C
**Shipping**	Blue Ice

Regulatory Status

RUO - Research Use Only

Product Details

**Alternative Name**	Endoplasmin, Tra1, Hsp90B1, Gp96
**Application**	Electron microscopy, ICC, IF, IHC (PS), IP, WB
**Application Notes**	Detects a band of ~98kDa by Western blot.
**Clone**	9G10
**Formulation**	Liquid. In PBS containing 50% glycerol and 0.09% sodium azide.
**GenBank ID**	M14772
**Host**	Rat
**Immunogen**	Native chicken Grp94.
**Isotype**	IgG2a
**Purity Detail**	Protein G affinity purified.
**Recommendation Dilutions/Conditions**	Immunoprecipitation (1:100)Western Blot (1:1,000, ECL)Suggested dilutions/conditions may not be available for all applications.Optimal conditions must be determined individually for each application.
**Source**	Purified from ascites.
**Species Reactivity**	Bovine, Chicken, Dog, Guinea pig, Hamster, Human, Monkey, Mouse, Porcine, Rabbit, Rat, Sheep, Xenopus
**U[illegible]Prot ID**	P08110

Technical Data Sheet

Purified Mouse Anti-GM130

Product Information

**Material Number:**	**610822**
**Size:**	50 µg
**Concentration:**	250 µg/ml
**Clone:**	35/GM130
**Immunogen:**	Rat GM130 aa. 869-982
**Isotype:**	Mouse IgG1, κ
**Reactivity:**	QC Testing: Rat<br>Tested in Development: Human, Dog, Mouse
**Target MW:**	130 kDa
**Storage Buffer:**	Aqueous buffered solution containing BSA, glycerol, and ≤0.09% sodium azide.

Description

Maturation and post-translational modification of proteins occurs after their biosynthesis at the endoplasmic reticulum and their transport through the Golgi apparatus. The process involves the transport of vesicles carrying the proteins through a vectorial process of vesicle budding and fusion from the *cis*-compartment to the *medial*-compartment and the *trans*-compartment of the Golgi apparatus. The detergent insoluble fraction of the Golgi is named "matrix" and is required for proper morphology of the Golgi membranes. GM130 (Golgi matrix protein of 130 kDa) is a protein isolated from the Triton™ X-100-insoluble Golgi matrix and peripherally associated with the *cis*-compartment, as demonstrated by co-localization with syntaxin5. GM130 is homologous to the Golgi autoantigen golgin 95. GM130 interacts through its N-terminal domain with p115 and with the Golgi membranes at the C-terminal portion. Furthermore, the mitotic phosphorylation of GM130 blocks the interaction with p115. Thus, GM130 appears to function as a structural element of the Golgi apparatus that also provides attachment sites for membranes and other Golgi proteins. The 35/GM130 monoclonal antibody recognizes GM130, regardless of phosphorylation status.

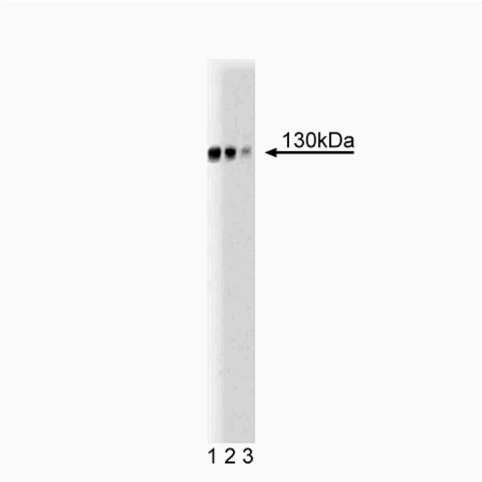

Western blot analysis of GM130 on rat brain lysate.
Lane 1: 1:250, lane 2: 1:500, lane 3: 1:1000 dilution of anti-GM130 antibody.

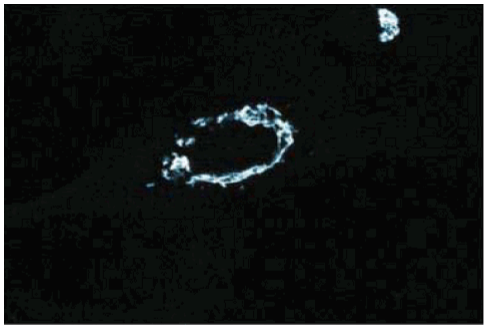

Immunofluorescent staining of WI-38 cells.

Preparation and Storage

The monoclonal antibody was purified from tissue culture supernatant or ascites by affinity chromatography.
Store undiluted at -20°C.

BD Biosciences
bdbiosciences.com

United States	Canada	Europe	Japan	Asia Pacific	Latin America/Caribbean
877.232.8995	888.259.0187	32.53.720.550	0120.8555.90	65.6861.0633	55.11.5185.9995

For country-specific contact information, visit **bdbiosciences.com/how_to_order/**

Conditions: The information disclosed herein is not to be construed as a recommendation to use the above product in violation of any patents. BD Biosciences will not be held responsible for patent infringement or other violations that may occur with the use of our products. Purchase does not include or carry any right to resell or transfer this product either as a stand-alone product or as a component of another product. Any use of this product other than the permitted use without the express written authorization of Becton Dickinson and Company is strictly prohibited.
For Research Use Only. Not for use in diagnostic or therapeutic procedures. Not for resale.
BD, BD Logo and all other trademarks are the property of Becton, Dickinson and Company. ©2008 BD

Application Notes

Application

Western blot	Routinely Tested
Immunofluorescence	Tested During Development
Immunohistochemistry	Not Recommended
Immunoprecipitation	Not Recommended

Recommended Assay Procedure:

Western blot: Please refer to http://www.bdbiosciences.com/pharmingen/protocols/Western_Blotting.shtml .

Suggested Companion Products

Catalog Number	Name	Size	Clone
611463	Rat Cerebrum Lysate	500 µg	(none)
554002	HRP Goat Anti-Mouse Ig	1.0 ml	(none)
554001	FITC Goat Anti-Mouse Ig	0.5 mg	Polyclonal

Product Notices

1. Since applications vary, each investigator should titrate the reagent to obtain optimal results.
2. Please refer to www.bdbiosciences.com/pharmingen/protocols for technical protocols.
3. Caution: Sodium azide yields highly toxic hydrazoic acid under acidic conditions. Dilute azide compounds in running water before discarding to avoid accumulation of potentially explosive deposits in plumbing.
4. Source of all serum proteins is from USDA inspected abattoirs located in the United States.

References

Ireton RC, Davis MA, van Hengel J, et al. A novel role for p120 catenin in E-cadherin function. *J Cell Biol.* 2002; 159(3):465-476.(Clone-specific: Western blot)

Marra P, Maffucci T, Daniele T, et al. The GM130 and GRASP65 Golgi proteins cycle through and define a subdomain of the intermediate compartment. *Nat Cell Biol.* 2001; 3(12):1101-1113.(Clone-specific: Immunofluorescence)

Nakamura N, Lowe M, Levine TP, Rabouille C, Warren G. The vesicle docking protein p115 binds GM130, a cis-Golgi matrix protein, in a mitotically regulated manner. *Cell.* 1997; 89(3):445-455.(Biology)

Perez F, Pernet-Gallay K, Nizak C, Goodson HV, Kreis TE, Goud B. CLIPR-59, a new trans-Golgi/TGN cytoplasmic linker protein belonging to the CLIP-170 family. *J Cell Biol.* 2002; 156(4):631-642.(Clone-specific: Immunofluorescence)

Ralston E, Lu Z, Ploug T. The organization of the Golgi complex and microtubules in skeletal muscle is fiber type-dependent. *J Neurosci.* 1999; 19(24):10694-10705.(Clone-specific: Immunohistochemistry)

Goat anti-Rabbit IgG (H+L) Highly Cross-Adsorbed Secondary Antibody, Alexa Fluor™ Plus 594

Product Details	
Size	1 mg
Species Reactivity	Rabbit
Host/Isotype	Goat / IgG
Class	Polyclonal
Type	Secondary Antibody
Conjugate	Alexa Fluor™ Plus 594
Excitation/Emission Max	590/618 nm
Immunogen	Gamma Immunoglobins Heavy and Light chains
Form	Liquid
Concentration	2 mg/mL
Purification	Affinity chromatography
Storage buffer	proprietary buffer, pH 6.5
Contains	0.016% Methylisothiazolone, 0.016% Bromonitrodioxane
Storage conditions	4° C, store in dark
RRID	AB_2762824

Applications	Tested Dilution	Publications
Immunohistochemistry (Paraffin) (IHC (P))	-	0 Publication
Immunohistochemistry (Frozen) (IHC (F))	-	0 Publication
Immunocytochemistry (ICC/IF)	1-10 µg/mL	0 Publication
Miscellaneous PubMed (Misc)	-	0 Publication

Product Specific Information

To minimize cross-reactivity, the goat anti-rabbit IgG whole antibodies have been cross-adsorbed against IgG from human, mouse and rat. Cross-adsorption or pre-adsorption is a purification step to increase specificity of the antibody resulting in less background staining and cross-reactivity. The secondary antibody solution is passed through a column matrix containing immobilized serum proteins from potentially cross-reactive species. Only the nonspecific-binding secondary antibodies are captured in the column, and the highly specific secondaries flow through. Further passages through additional columns result in highly cross-adsorbed preparations of secondary antibody. The benefits of these extra steps are apparent in multiplexing /multicolor-staining experiments where there is potential cross-reactivity with other primary antibodies or in tissue/cell fluorescent staining experiments where there may be the presence of endogenous immunoglobulins.

Using conjugate solutions: Centrifuge the protein conjugate solution briefly in a microcentrifuge before use; add only the supernatant to the experiment. This step will help eliminate any protein aggregates that may have formed during storage, thereby reducing nonspecific background staining. Because staining protocols vary with application, the appropriate dilution of antibody should be determined empirically

Specificity: This antibody binds to whole molecule rabbit IgG and light chains on other rabbit immunoglobulins. This antibody does not bind non-immunoglobulin serum proteins from human, mouse and rat. It has been pre-adsorbed for minimal cross-reactivity with IgG from human, mouse and rat sources.

Product Images For Goat anti-Rabbit IgG (H+L) Highly Cross-Adsorbed Secondary Antibody, Alexa Fluor™ Plus 594

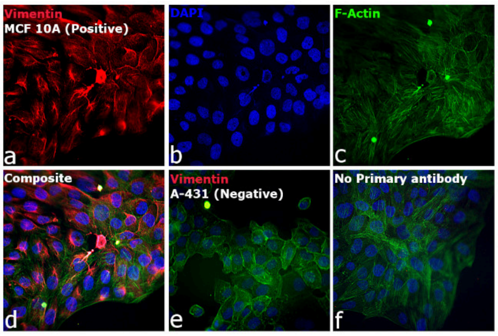

Rabbit IgG (H+L) Highly Cross-Adsorbed Secondary Antibody (A32740) in ICC/IF

Immunofluorescence analysis of Goat anti-Rabbit IgG (H+L) Highly Cross-Adsorbed Secondary Antibody, Alexa Fluor Plus 594 (Product # A32740) was performed using MCF 10A (positive model) and A-431 (negative model) cells stained with Vimentin Polyclonal Antibody (Product # PA5-27231). The cells were fixed with 4% paraformaldehyde for 10 minutes, permeabilized with 0.1% Triton™ X-100 for 10 minutes, blocked with 1% BSA for 1 hour and labeled with 2 µg/mL primary antibody for 3 hours at room temperature. Goat anti-Rabbit IgG (H+L) Highly Cross-Adsorbed Secondary Antibody, Alexa Fluor Plus 594 (Product # A32740, 1:2000 dilution) in 0.1% BSA in PBS for 45 minutes at room temperature, was used for detection of Vimentin in the cytoplasm (Panel a: Red). Nuclei (Panel b: blue) were stained with Hoechst33342 (Product # H1399). F-actin was stained with Alexa Fluor® 488 Phalloidin (Product # A12379, 1:300) (Panel c: green). Panel d represents the composite image. The specificity of the secondary antibody was proved by the absence of signal in A-431 (negative model for vimentin) due to no primary antibody binding (Panel e). Nonspecific staining was not observed with secondary antibody alone (panel f). The images were captured at 40X magnification in CellInsight CX7 LZR High-Content Screening (HCS) Platform (Product # CX7A1110LZR) and externally deconvoluted (D.Sage et al./Methods 115 (2017) 28–41).

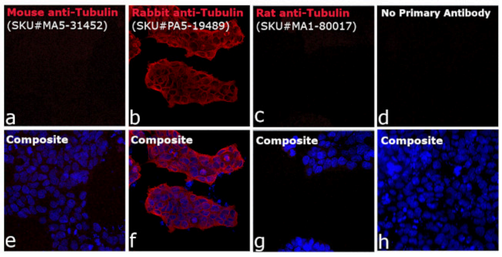

Rabbit IgG (H+L) Highly Cross-Adsorbed Secondary Antibody (A32740) in ICC/IF

Immunofluorescence analysis of A32740 was performed using anti-alpha tubulin antibodies in 70% confluent log phase HEK 293 cells. The cells were fixed with 4% Paraformaldehyde, permeabilized with 0.1% Triton X-100 and blocked with 2% BSA, then incubated with primary antibodies at 1:100 dilution at 4 degree celsius overnight. The cells were then incubated with Goat anti-Rabbit IgG (H+L) Highly Cross-Adsorbed Secondary Antibody, Alexa Fluor Plus 594 (Product # A32740) at 1:2000 dilution in 0.1% BSA at room temperature for 45 minutes. The images were captured at 40X magnification in CellInsight CX7 LZR High-Content Screening (HCS) Platform (Product # CX7A1110LZR) and externally deconvoluted (D.Sage et al./Methods 115 (2017) 28–41). Cytoskeletal localization of alpha-tubulin was observed only in cells stained with Rabbit alpha-Tubulin antibody (Product # PA5-19489) (Panels b and d), and not in the cells stained with Mouse alpha-Tubulin antibody (Product # MA5-31452) (Panels a and e) or Rat alpha-Tubulin antibody (Product # MA1-80017) (Panels c and g), demonstrating the host specific reactivity of A32740. Nuclei (blue) were stained with Hoechst33342 (Product # H1399). Panels d and h represent control cells with no primary antibody.

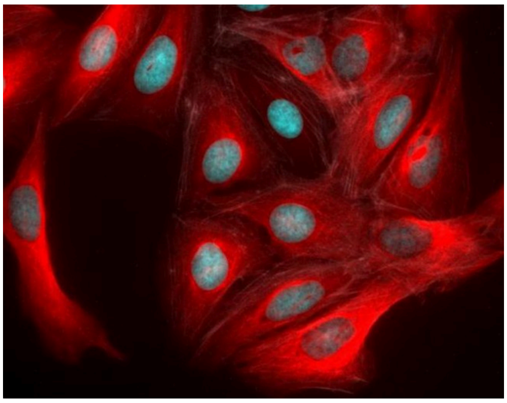

Rabbit IgG (H+L) Highly Cross-Adsorbed Secondary Antibody (A32740) in ICC/IF

Immunofluorescent analysis of tubulin in U2OS cells. The cells were fixed with 4% formaldehyde for 20 mins, permeabilized with 0.5% Triton X-100 in PBS for 20 mins, washed 3X in PBS and blocked with 3% BSA in PBS for 30 mins at RT. Cells were stained with a tubulin antibody at a dilution of 1:1000 in 3% BSA in PBS for 1 hr at RT, washed 3X in PBS and then incubated with Invitrogen Alexa Fluor Plus 594 goat anti-rabbit IgG secondary antibody (Product # A32740) prepared in 3% BSA in PBS at a dilution of 1:1000 for 1 hr at RT in the presence of NucBlue Live ReadyProbes Reagent (Product # R37605). The image contains overlay of tubulin (red) and nuclei (blue). Images were taken on an EVOS FL Auto 2 Imaging System (Product # AMAFD2000) with an Olympus 20X Super Apochromat objective (Product # AMEP4754) at 40X magnification. Actin was stained using Alexa Fluor Plus Phalloidin (Product # A30105)

View more figures on thermofisher.com

325 References

Non-cell autonomous regulation of cell-cell signaling and differentiation by mitochondrial ROS. J Cell Biol (2024)

Chemokine expression profile of an innate granuloma. Elife (2024)

Flavopiridol induces cell cycle arrest and apoptosis by interfering with CDK1 signaling pathway in human ovarian granulosa cells. Sci Rep (2024)

CCR7 depletion alleviates bony growth imbalance following physeal injury in mice. Sci Rep (2024)

Critical-Size Defect Tibialis Anterior (TA) Muscle Regeneration using Ex-Vivo Mice Hindlimbs Culturing under Dynamic Mechanical Loading bioRxiv (2024)

For Research Use Only. Not for use in diagnostic procedures. Not for resale without express authorization. Products are warranted to operate or perform substantially in conformance with published Product specifications in effect at the time of sale, as set forth in the Production documentation, specifications and/or accompanying package inserts ("Documentation"). No claim of suitability for use in applications regulated by FDA is made. The warranty provided herein is valid only when used by properly trained individuals. Unless otherwise stated in the Documentation, this warranty is limited to one year from date of shipment when the Product is subjected to normal, proper and intended usage. This warranty does not extend to anyone other than the Buyer. Any model or sample furnished to Buyer is merely illustrative of the general type and quality of goods and does not represent that any Product will conform to such model or sample. NO OTHER WARRANTIES, EXPRESS OR IMPLED, ARE GRANTED INCLUDING WITHOUT LIMITATION, IMPLIED WARRANTIES OF MERCHANTABILITY, FITNESS FOR ANY PARTICULAR PURPOSE, OR NON INFRINGEMENT. BUYER'S EXCLUSIVE REMEDY FOR NON-CONFORMING PRODUCTS DURING THE WARRANTY PERIOD IS LIMITED TO REPAIR, REPLACEMENT OF OR REFUND FOR THE NON-CONFORMING PRODUCT(S) AT SELLER'S SOLE OPTION. THERE IS NO OBLIGATION TO REPAIR, REPLACE OR REFUND FOR PRODUCTS AS THE RESULT OF (I) ACCIDENT, DISASTER OR EVENT OF FORCE MAJEURE, (II) MISUSE, FAULT OR NEGLIGENCE OF OR BY BUYER, (III) USE OF THE PRODUCTS IN A MANNER FOR WHICH THEY WERE NOT DESIGNED, OR (IV) IMPROPER STORAGE AND HANDLING OF THE PRODUCTS. Unless otherwise expressly stated on the Product or in the documentation accompanying the Product, the Product is intended for research only and is not to be used for any other purpose, including without limitation, unauthorized commercial uses, in vitro diagnostic uses, ex vivo or in vivo therapeutic uses, or any type of consumption by or application to human or animals.

Goat anti-Rat IgG (H+L) Cross-Adsorbed Secondary Antibody, Alexa Fluor™ 488

Product Details	
Size	1 mg
Species Reactivity	Rat
Host/Isotype	Goat / IgG
Class	Polyclonal
Type	Secondary Antibody
Conjugate	Alexa Fluor™ 488
Excitation/Emission Max	499/520 nm
Immunogen	Gamma Immunoglobins Heavy and Light chains
Form	Liquid
Concentration	2 mg/mL
Purification	purified
Storage buffer	PBS, pH 7.5
Contains	5mM sodium azide
Storage conditions	4° C, store in dark
RRID	AB_2534074

Applications	Tested Dilution	Publications
Western Blot (WB)	-	0 Publication
Immunohistochemistry (IHC)	-	0 Publication
Immunohistochemistry (Paraffin) (IHC (P))	-	0 Publication
Immunohistochemistry (PFA fixed) (IHC (PFA))	-	0 Publication
Immunohistochemistry (Frozen) (IHC (F))	-	0 Publication
Immunohistochemistry - Free Floating (IHC (Free))	-	0 Publication
Immunocytochemistry (ICC/IF)	4 µg/mL	0 Publication
Flow Cytometry (Flow)	1-10 µg/mL	0 Publication
Miscellaneous PubMed (Misc)	-	0 Publication
Not applicable (N/A)	-	0 Publication

Product Specific Information

To minimize cross-reactivity, these goat anti-rat IgG whole antibodies have been cross-adsorbed against mouse IgG, mouse serum, and human serum prior to conjugation. Cross-adsorption or pre-adsorption is a purification step to increase specificity of the antibody resulting in higher sensitivity and less background staining. The secondary antibody solution is passed through a column matrix containing immobilized serum proteins from potentially cross-reactive species. Only the nonspecific-binding secondary antibodies are captured in the column, and the highly specific secondaries flow through. The benefits of this extra step are apparent in multiplexing/multicolor-staining experiments (e.g., flow cytometry) where there is potential cross-reactivity with other primary antibodies or in tissue/cell fluorescent staining experiments where there are may be the presence of endogenous immunoglobulins.

Alexa Fluor dyes are among the most trusted fluorescent dyes available today. Invitrogen™ Alexa Fluor 488 dye is a bright, green-fluorescent dye with excitation ideally suited to the 488 nm laser line. For stable signal generation in imaging and flow cytometry, Alexa Fluor 488 dye is pH-insensitive over a wide molar range. Probes with high fluorescence quantum yield and high photostability allow detection of low-abundance biological structures with great sensitivity. Alexa Fluor 488 dye molecules can be attached to proteins at high molar ratios without significant self-quenching, enabling brighter conjugates and more sensitive detection. The degree of labeling for each conjugate is typically 2-8 fluorophore molecules per IgG molecule; the exact degree of labeling is indicated on the certificate of analysis for each product lot.

Using conjugate solutions: Centrifuge the protein conjugate solution briefly in a microcentrifuge before use; add only the supernatant to the experiment. This step will help eliminate any protein aggregates that may have formed during storage, thereby reducing nonspecific background staining. Because staining protocols vary with application, the appropriate dilution of antibody should be determined empirically. For the fluorophore-labeled antibodies a final concentration of 1-10 µg/mL should be satisfactory for most immunohistochemistry and flow cytometry applications.

Product will be shipped at Room Temperature.

Product Images For Goat anti-Rat IgG (H+L) Cross-Adsorbed Secondary Antibody, Alexa Fluor™ 488

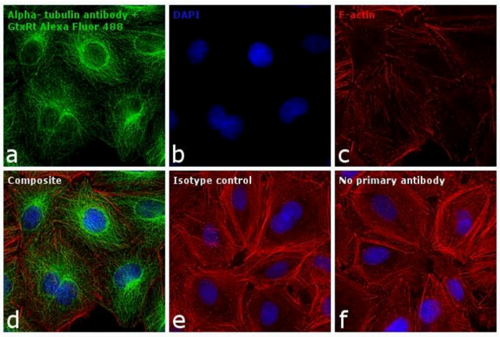

Rat IgG (H+L) Cross-Adsorbed Secondary Antibody (A-11006) in ICC/IF
Immunofluorescence analysis of Goat anti-Rat IgG (H+L) Cross-Adsorbed Secondary Antibody, Alexa Fluor® 488 conjugate was performed using A549 cells stained with alpha Tubulin (YL1/2) Rat Monoclonal Antibody (Product # MA1-80017). The cells were fixed with 4% paraformaldehyde for 10 minutes, permeabilized with 0.1% Triton™ X-100 for 10 minutes, blocked with 1% BSA for 1 hour and labeled with 2 µg/mL primary antibody for 3 hours at room temperature. Goat anti-Rat IgG (H+L) Cross-Adsorbed Secondary Antibody, Alexa Fluor® 488 conjugate (Product # A-11006) was used at a concentration of 4 µg/mL in phosphate buffered saline containing 0.2% BSA for 45 minutes at room temperature, for detection of alpha Tubulin in the cytoplasm (Panel a: green). Nuclei (Panel b: blue) were stained with DAPI in SlowFade® Gold Antifade Mountant (Product # S36938). F-actin was stained with Rhodamine Phalloidin (Product # R415, 1:300) (Panel c: red). Panel d represents the composite image. No nonspecific staining was observed with the secondary antibody alone (panel f), or with an isotype control (panel e). The images were captured at 60X magnification.

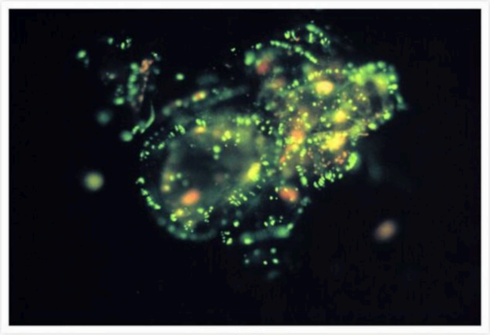

Rat IgG (H+L) Cross-Adsorbed Secondary Antibody (A-11006) in ICC/IF
The abundance of pectin associated with the plasmodesmatal pit fields of kiwifruit cells. Pectin, a component of the cell wall matrix and the main constituent of the middle lamella that forms between daughter cell walls, was tagged with an anti-pectin monoclonal antibody, JIM 5. The primary antibody was detected and visualized with Alexa Fluor® 488 goat anti-rat IgG (Product # A-11006). The primary antibody was a gift from Dr. Paul Knox, University of Leeds, U.K. Image contributed by Paul Sutherland, The Horticulture and Food Research Institute of New Zealand, Ltd., Mt. Albert Research Centre.

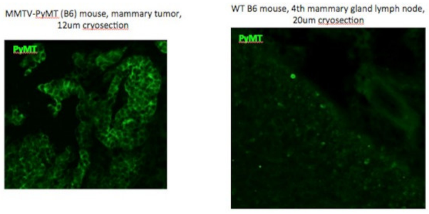

Rat IgG (H+L) Cross-Adsorbed Secondary Antibody (A-11006) in ICC/IF
Immunofluorescence analysis of Polyoma Virus Medium T (green) in PyMT+ mammary tumor cells and mammary gland lymph node. Cells were stained with a Polyoma Virus Medium T Monoclonal Antibody (Product # MA1-46061) at a dilution of 1:500 overnight at 4C, and then incubated with secondary goat anti-rat IgG - Alexa Fluor 488 antibody (Product # A-11006) at a dilution of 1:500 for 1 hour. Data courtesy of Antibody Data Exchange Program.

View more figures on thermofisher.com

2308 References

Small heterodimer partner-interacting leucine zipper protein suppresses pain and cartilage destruction in an osteoarthritis model by modulating the AMPK/STAT3 signaling pathway. Arthritis Res Ther (2024)

Cell type mapping reveals tissue niches and interactions in subcortical multiple sclerosis lesions. Nat Neurosci (2024)

Neutrophil pyroptosis regulates corneal wound healing and post-injury neovascularisation. Clin Transl Med (2024)

Spatial quantitative metabolomics enables identification of remote and sustained ipsilateral cortical metabolic reprogramming after stroke bioRxiv (2024)

Synaptic input architecture of visual cortical neurons revealed by large-scale synapse imaging without backpropagating action potentials bioRxiv (2024)

For Research Use Only. Not for use in diagnostic procedures. Not for resale without express authorization. Products are warranted to operate or perform substantially in conformance with published Product specifications in effect at the time of sale, as set forth in the Production documentation, specifications and/or accompanying package inserts ("Documentation"). No claim of suitability for use in applications regulated by FDA is made. The warranty provided herein is valid only when used by properly trained individuals. Unless otherwise stated in the Documentation, this warranty is limited to one year from date of shipment when the Product is subjected to normal, proper and intended usage. This warranty does not extend to anyone other than the Buyer. Any model or sample furnished to Buyer is merely illustrative of the general type and quality of goods and does not represent that any Product will conform to such model or sample. NO OTHER WARRANTIES, EXPRESS OR IMPLED, ARE GRANTED INCLUDING WITHOUT LIMITATION, IMPLIED WARRANTIES OF MERCHANTABILITY, FITNESS FOR ANY PARTICULAR PURPOSE, OR NON INFRINGEMENT. BUYER'S EXCLUSIVE REMEDY FOR NON-CONFORMING PRODUCTS DURING THE WARRANTY PERIOD IS LIMITED TO REPAIR, REPLACEMENT OF OR REFUND FOR THE NON-CONFORMING PRODUCT(S) AT SELLER'S SOLE OPTION. THERE IS NO OBLIGATION TO REPAIR, REPLACE OR REFUND FOR PRODUCTS AS THE RESULT OF (I) ACCIDENT, DISASTER OR EVENT OF FORCE MAJEURE, (II) MISUSE, FAULT OR NEGLIGENCE OF OR BY BUYER, (III) USE OF THE PRODUCTS IN A MANNER FOR WHICH THEY WERE NOT DESIGNED, OR (IV) IMPROPER STORAGE AND HANDLING OF THE PRODUCTS. Unless otherwise expressly stated on the Product or in the documentation accompanying the Product, the Product is intended for research only and is not to be used for any other purpose, including without limitation, unauthorized commercial uses, in vitro diagnostic uses, ex vivo or in vivo therapeutic uses, or any type of consumption by or application to human or animals.

Goat anti-Mouse IgG (H+L) Highly Cross-Adsorbed Secondary Antibody, Alexa Fluor™ 488

Product Details	
Size	1 mg
Species Reactivity	Mouse
Host/Isotype	Goat / IgG
Class	Polyclonal
Type	Secondary Antibody
Conjugate	Alexa Fluor™ 488
Excitation/Emission Max	499/520 nm
Immunogen	Gamma Immunoglobins Heavy and Light chains
Form	Liquid
Concentration	2 mg/mL
Purification	purified
Storage buffer	PBS, pH 7.5
Contains	5mM sodium azide
Storage conditions	4° C, store in dark
RRID	AB_2534088

Applications	Tested Dilution	Publications
Immunohistochemistry (IHC)	Assay-dependent	0 Publication
Immunohistochemistry (Paraffin) (IHC (P))	-	0 Publication
Immunohistochemistry (PFA fixed) (IHC (PFA))	-	0 Publication
Immunohistochemistry (Frozen) (IHC (F))	-	0 Publication
Immunocytochemistry (ICC/IF)	1-10 µg/mL	0 Publication
Flow Cytometry (Flow)	1-10 µg/mL	0 Publication
Miscellaneous PubMed (Misc)	-	0 Publication
Not applicable (N/A)	-	0 Publication

Product Specific Information

To minimize cross-reactivity, the goat anti-mouse IgG whole antibodies have been highly cross-adsorbed against bovine IgG, goat IgG, rabbit IgG, rat IgG, human IgG, and human serum. Cross-adsorption or pre-adsorption is a purification step to increase specificity of the antibody resulting in higher sensitivity and less background staining. The secondary antibody solution is passed through a column matrix containing immobilized serum proteins from potentially cross-reactive species. Only the nonspecific-binding secondary antibodies are captured in the column, and the highly specific secondaries flow through. Further passages through additional columns result in 'highly cross-adsorbed' preparations of secondary antibody. The benefits of these extra steps are apparent in multiplexing/multicolor-staining experiments where there is potential cross-reactivity with other primary antibodies or in tissue/cell fluorescent staining experiments where there may be the presence of endogenous immunoglobulins.

Alexa Fluor dyes are among the most trusted fluorescent dyes available today. Invitrogen™ Alexa Fluor 488 dye is a bright, green-fluorescent dye with excitation ideally suited to the 488 nm laser line. For stable signal generation in imaging and flow

cytometry, Alexa Fluor 488 dye is pH-insensitive over a wide molar range. Probes with high fluorescence quantum yield and high photostability allow detection of low-abundance biological structures with great sensitivity. Alexa Fluor 488 dye molecules can be attached to proteins at high molar ratios without significant self-quenching, enabling brighter conjugates and more sensitive detection. The degree of labeling for each conjugate is typically 2-8 fluorophore molecules per IgG molecule; the exact degree of labeling is indicated on the certificate of analysis for each product lot.

Using conjugate solutions: Centrifuge the protein conjugate solution briefly in a microcentrifuge before use; add only the supernatant to the experiment. This step will help eliminate any protein aggregates that may have formed during storage, thereby reducing nonspecific background staining. Because staining protocols vary with application, the appropriate dilution of antibody should be determined empirically. For the fluorophore-labeled antibodies a final concentration of 1-10 µg/mL should be satisfactory for most immunohistochemistry and flow cytometry applications.

Product will be shipped at Room Temperature.

Product Images For Goat anti-Mouse IgG (H+L) Highly Cross-Adsorbed Secondary Antibody, Alexa Fluor™ 488

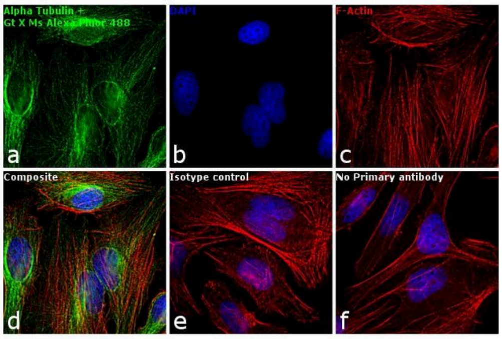

Mouse IgG (H+L) Highly Cross-Adsorbed Secondary Antibody (A-11029) in ICC/IF

Immunofluorescence analysis of Goat anti-Mouse IgG (H+L) Highly Cross-Adsorbed Secondary Antibody Alexa Fluor® 488 conjugate was performed using HeLa cells stained with alpha Tubulin (236-10501) Mouse Monoclonal Antibody (Product # A11126). The cells were fixed with 4% paraformaldehyde for 10 minutes, permeabilized with 0.1% Triton™ X-100 for 10 minutes, blocked with 1% BSA for 1 hour and labeled with 2 µg/mL Mouse primary antibody for 3 hours at room temperature. Goat anti-Mouse IgG (H+L) Highly Cross-Adsorbed Secondary Antibody Alexa Fluor® 488 conjugate (A-11029) was used at a concentration of 1 µg/mL in phosphate buffered saline containing 0.2% BSA for 45 minutes at room temperature, for detection of alpha Tubulin in the cytoplasm (Panel a: green). Nuclei (Panel b: blue) were stained with DAPI in SlowFade® Gold Antifade Mountant (Product # S36938). F-actin was stained with Rhodamine Phalloidin (Product # R415, 1:300) (Panel c: red). Panel d represents the composite image. No nonspecific staining was observed with the secondary antibody alone (panel f), or with an isotype control (panel e). The images were captured at 60X magnification.

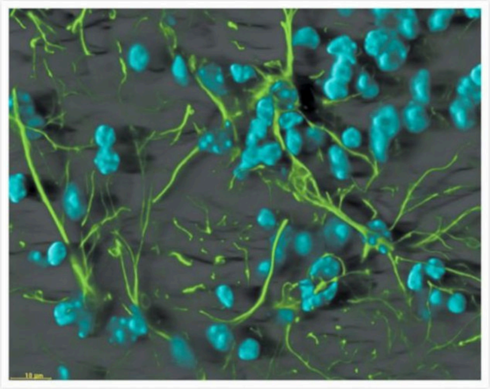

Mouse IgG (H+L) Highly Cross-Adsorbed Secondary Antibody (A-11029) in ICC/IF

Intermediate filaments of astrocytes and ependymal cells in a mouse brain cryosection identified using mouse monoclonal anti-GFAP and visualized with Alexa Fluor® 488 goat anti-mouse IgG antibody. Intermediate filaments of astrocytes and ependymal cells in a 14 µm mouse brain cryosection were identified using mouse monoclonal anti-glial fibrillary monoclonal antibody (anti-GFAP, Product # A-21282) and visualized with green-fluorescent Alexa Fluor® 488 goat anti-mouse IgG antibody (Product # A-11029). Nuclei were stained with blue-fluorescent DAPI (Product # D1306, D3571, D21490). The image was deconvolved using Huygens software (Scientific Volume Imaging, http://www.svi.nl/). 3-D reconstruction was performed using Imaris software (Bitplane AG, http://www.bitplane.com/).

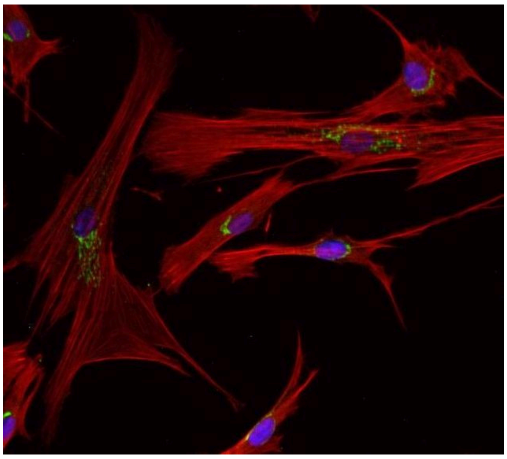

Mouse IgG (H+L) Highly Cross-Adsorbed Secondary Antibody (A-11029) in ICC/IF

Human dermal fibroblasts, neonatal (HDFn) (C-004-5C) were fixed and permeabilized using the Image-iT® Fixation/Permeabilization Kit (Product # R37602). Golgi staining was done using an anti-Golgin-97 primary antibody (Product # A-21270) and a goat anti-mouse Alexa Fluor® 488 secondary antibody (Product # A-11029). Actin was stained using Alexa Fluor® 594 phalloidin (Product # A12381) and nuclei were stained using NucBlue™ Live Cell Stain (Product # R37605). Slides were mounted using ProLong® Gold antifade kit (P7481) and images were acquired on the FLoid™ Cell Imaging Station (Product # 4471136).

View more figures on thermofisher.com

5755 References

Characterizing the effects of Dechlorane Plus on -cells: a comparative study across models and species. Islets (2024)

NAD+ depletion is central to placental dysfunction in an inflammatory subclass of preeclampsia. Life Sci Alliance (2024)

Novel ectopic expression of zona pellucida 3 glycoprotein in lung cancer promotes tumor growth. Int J Cancer (2024)

Age-dependent cerebral vasodilation induced by volatile anesthetics is mediated by NG2+ vascular mural cells. Commun Biol (2024)

Dynamin independent endocytosis is an alternative cell entry mechanism for multiple animal viruses. PLoS Pathog (2024)

For Research Use Only. Not for use in diagnostic procedures. Not for resale without express authorization. Products are warranted to operate or perform substantially in conformance with published Product specifications in effect at the time of sale, as set forth in the Production documentation, specifications and/or accompanying package inserts ("Documentation"). No claim of suitability for use in applications regulated by FDA is made. The warranty provided herein is valid only when used by properly trained individuals. Unless otherwise stated in the Documentation, this warranty is limited to one year from date of shipment when the Product is subjected to normal, proper and intended usage. This warranty does not extend to anyone other than the Buyer. Any model or sample furnished to Buyer is merely illustrative of the general type and quality of goods and does not represent that any Product will conform to such model or sample. NO OTHER WARRANTIES, EXPRESS OR IMPLED, ARE GRANTED INCLUDING WITHOUT LIMITATION, IMPLIED WARRANTIES OF MERCHANTABILITY, FITNESS FOR ANY PARTICULAR PURPOSE, OR NON INFRINGEMENT. BUYER'S EXCLUSIVE REMEDY FOR NON-CONFORMING PRODUCTS DURING THE WARRANTY PERIOD IS LIMITED TO REPAIR, REPLACEMENT OF OR REFUND FOR THE NON-CONFORMING PRODUCT(S) AT SELLER'S SOLE OPTION. THERE IS NO OBLIGATION TO REPAIR, REPLACE OR REFUND FOR PRODUCTS AS THE RESULT OF (I) ACCIDENT, DISASTER OR EVENT OF FORCE MAJEURE, (II) MISUSE, FAULT OR NEGLIGENCE OF OR BY BUYER, (III) USE OF THE PRODUCTS IN A MANNER FOR WHICH THEY WERE NOT DESIGNED, OR (IV) IMPROPER STORAGE AND HANDLING OF THE PRODUCTS. Unless otherwise expressly stated on the Product or in the documentation accompanying the Product, the Product is intended for research only and is not to be used for any other purpose, including without limitation, unauthorized commercial uses, in vitro diagnostic uses, ex vivo or in vivo therapeutic uses, or any type of consumption by or application to human or animals.

Optilite®
OPTIMISED PROTEIN SYSTEM

Optilite® Caeruloplasmin Kit

IVD **For professional use only.**

REF **NK045.OPT**

1 INTENDED USE

The Optilite Caeruloplasmin Kit is intended for the quantitative *in vitro* measurement of caeruloplasmin in serum and lithium heparin plasma using the Binding Site Optilite analyser to aid the diagnosis of copper metabolism disorders. This test should be used in conjunction with other laboratory and clinical findings.

2 SUMMARY AND EXPLANATION

Caeruloplasmin is synthesised in the liver and has a major role in copper metabolism, carrying approximately 95% of the total copper in serum. Decreased levels of caeruloplasmin can be caused by hereditary disorders of copper metabolism, for example; inability to transport oxidised copper (Cu^{2+}) from the gastrointestinal epithelium into the circulation, as in Menkes disease (Ref 1). Or, the inability to insert Cu^{2+} into the developing caeruloplasmin molecule, as in Wilson's disease (Ref 1). Dietary copper insufficiency, including malabsorption, also reduces serum caeruloplasmin concentrations. Serum caeruloplasmin concentrations can increase as a result of acute-phase reactions, pregnancy or use of oral contraceptives (Ref 2).

3 PRINCIPLE

The determination of soluble antigen concentration by turbidimetric methods involves the reaction with specific antiserum to form insoluble complexes. When light is passed through the suspension formed a portion of the light is transmitted and focused onto a photodiode by an optical lens system. The amount of transmitted light is indirectly proportional to the specific protein concentration in the test sample. Concentrations are automatically calculated by reference to a calibration curve stored within the instrument.

4 REAGENTS

4.1 Antiserum: Supplied in stabilised liquid form. Preservatives: 0.099% sodium azide, 0.1% E-amino-n-caproic acid (EACA), 0.1% EDTA and 0.01% benzamidine.

4.2 Calibrator and Controls: Pooled human serum, supplied in lyophilised form. Containing 0.099% sodium azide, 0.1% EACA and 0.01% benzamidine as preservatives. The concentration given on the quality control certificate has been obtained by comparison to an internal master calibrator.

4.3 Reaction Buffer: Containing 0.099% sodium azide as a preservative.

5 CAUTION

All donors of human serum supplied in this kit have been serum tested and found negative for hepatitis B surface antigen (HBsAg) and antibodies to human immunodeficiency virus (HIV1 and HIV2) and hepatitis C virus. The assays used were either cleared by the FDA (USA) or cleared for *in vitro* diagnostic use in the EU (Directive 98/79/EC, Annex II); however, these tests cannot guarantee the absence of infective agents. Proper handling and disposal methods should be established as for all potentially infective material, including (but not limited to) users wearing suitable protective equipment and clothing at all times. Only personnel fully trained in such methods should be permitted to perform these procedures.

WARNING: This product contains sodium azide and must be handled with caution; suitable gloves and other protective clothing should be worn at all times when handling this product. Do not ingest or allow contact with the skin (particularly broken skin or open wounds) or mucous membranes. If contact does occur wash with a large volume of water and urgently seek medical advice. Explosive metal azides may be formed on prolonged contact of sodium azide with lead and copper plumbing; on disposal of reagent, flush with a large volume of water to prevent azide build up.

This product should only be used by suitably trained personnel for the purposes stated in the Intended Use. Strict adherence to these instructions is essential at all times. Results are likely to be invalid if parameters other than those stated in these instructions are used.

Reagents from different batch numbers of kits are **NOT** interchangeable.

6 STORAGE AND STABILITY

The unopened kit should be stored at 2-8°C and can be used until the expiry date shown on the kit box label. DO NOT FREEZE. The Reagent may be stored for up to three months after opening provided that it is capped to avoid evaporation and kept at 2-8°C in a refrigerator. Once reconstituted, the caeruloplasmin Calibrator and Controls must be stored at 2-8°C. The calibrator must be used within 7 days and the controls within 30 days. Discard any unused calibrator and controls after this time.

7 SPECIMEN COLLECTION AND PREPARATION

Samples should be obtained by venepuncture and in the case of plasma separated as soon as possible. Blood should be allowed to clot and the serum separated as soon as possible to prevent haemolysis. Samples may be stored at 2-8°C for up to three days prior to assay. For prolonged storage sera may be stored for up to four weeks at -20°C provided they are frozen within 24 hours after collection (Ref 3). Repeated freeze/thaw cycles should be avoided. Microbially contaminated, haemolysed and lipaemic samples and samples containing particulate matter should not be used. It is the responsibility of the individual laboratory to use all available references and/or its own studies to determine specific stability criteria for its laboratory.

8 METHODOLOGY

8.1 Materials provided

8.1.1 1 x 50 Tests *Optilite Cp Reagent*
8.1.2 2 x *Optilite Cp Calibrator*
8.1.3 2 x *Optilite Cp High Control*
8.1.4 2 x *Optilite Cp Low Control*

8.2 Materials required but not provided

8.2.1 Equipment for collection and preparation of test samples e.g. sample tubes, centrifuge etc.
8.2.2 A fully operational and equipped Optilite analyser.
8.2.3 Current analyser operating instructions: Optilite Operation Manual, Insert Code INS700.OPT
8.2.4 Optilite Diluent 2, Product Code IK710
8.2.5 Distilled water

8.3 Calibrator, Controls and Reagent Preparation

8.3.1 The calibrator and controls are supplied in lyophilised form. Each vial must be reconstituted in the volume of distilled water stated on the Quality Control Certificate (QCcert045.OPT) and vial label. Remove the cap and gently tap down all lyophilised material to the bottom of the vial. Add the required volume of distilled water and leave to stand for 20 minutes. Invert the vial and allow the fluid to cover the stopper for 20-30 seconds with gentle shaking. Return the vial to the upright position and leave to stand for 10 minutes, gently shake before use.
8.3.2 Before loading the reagent, gently mix by inversion ensuring no foam or bubbles are generated or remain on the surface as these may interfere with reagent aspiration.

8.4 Updating the Calibrator and QC Lot Information Using the QC Certificate (QCcert045.OPT)

The user should be familiar with the operation of the Optilite analyser before attempting to carry out the test procedures. The analyser should be prepared for use according to the instruction in the Optilite Operation Manual.

8.4.1 Go to **F4 – 4 Cal/Ctrl definition** and select **Cp Cal**.
8.4.2 Click [+].
8.4.3 Scan Barcode 1 from QCcert045.OPT
8.4.4 Click **Save**.
8.4.5 Click the pencil icon.
8.4.6 Ensure the new lot is ticked as the **Current lot**.
8.4.7 Click **Save**.
8.4.8 In the **Lot concentrations** tab click [+] and select the Caeruloplasmin **(Cp)** test.
8.4.9 Click into the **[Concentration]** field.
8.4.10 Scan Barcode 2 from QCcert045.OPT.
8.4.11 Click **Save**.
8.4.12 Select the relevant **Cp** control
8.4.13 In the **Cal/ctrl and lot** tab Click [+].
8.4.14 Scan Barcode 3 from QCcert045.OPT
8.4.15 Click **Save**.
8.4.16 Click the pencil icon.
8.4.17 Ensure the new lot is ticked as the **Current lot**.
8.4.18 Click **Save**.
8.4.19 In the **Lot concentrations** tab click [+] and select the Caeruloplasmin **(Cp)** test.
8.4.20 Click into the **[Concentration]** field.
8.4.21 Scan Barcode 4 from QCcert045.OPT for the relevant QC.
8.4.22 Click **Save**.
8.4.23 Repeat steps 8.4.12 – 8.4.22 for the other QC.

8.5 Loading the Calibrator and Controls

8.5.1 Dispense 300µL of calibrator and 200µL of each QC into separate 0.5ml sample cups and load as detailed in the Optilite Operation Manual (INS700.OPT).

8.6 Measuring range

The approximate measuring range of the assay is shown in the table below.

Optilite Analyser Dilution	Approximate range (g/L)
**1+9**	**0.04 – 0.82**
1+19	0.08 – 1.64

9 QUALITY CONTROL

At least two levels of appropriate control material should be tested a minimum of once a day. In addition, controls should be tested after calibration, with each new lot of reagent and after specific maintenance or troubleshooting steps described in the Optilite Operation Manual.

Quality control testing should be performed in accordance with regulatory requirements and each laboratory's standard procedure.

The concentrations of the controls provided are stated on the accompanying QC certificate (QCcert045.OPT). Sample results obtained should only be accepted if the control results are within ±15% of the concentration(s) stated.

Should a control measurement be out of range when assayed with a stored curve the assay must be recalibrated. If on recalibration the control values measured with the new curve are still out of range, the instrument and the assay parameters should be checked before repeating the assay. If problems persist, refer to the local technical support organisation.

Binding Site

10 LIMITATIONS

10.1 Turbidimetric assays are not suitable for measurement of highly lipaemic or haemolysed samples or samples containing high levels of circulating immune complexes (CICs) due to the unpredictable degree of non-specific scatter these sample types may generate. Unexpected results should be confirmed using an alternative assay method.

10.2 Diagnosis cannot be made and treatment must not be given on the basis of caeruloplasmin measurements alone. Clinical history and other laboratory findings must be taken into account.

10.3 This assay has not been validated using paediatric samples.

10.4 Should a control measurement be out of range when assayed with a stored curve the assay must be recalibrated. If on recalibration the control values measured with the new curve are still out of range, the instrument and the assay parameters should be checked before repeating the assay. If problems persist, refer to supplier.

10.5 Variation in reagent temperature may affect results. Ensure that reagents are transferred directly from the refrigerator to the refrigerated reagent compartment of the analyser – do not allow to warm to room temperature.

11 EXPECTED VALUES

The ranges provided have been obtained from a limited number of samples and are intended for guidance purposes only. Expected values may vary with age, sex, sample type, diet and geographical location. Each laboratory should verify the transferability of the expected values to its own population and, if necessary, determine its own reference interval.

Adult serum range

The literature reference interval of caeruloplasmin in serum is 0.2 – 0.6g/L (Ref. 4). This was verified using 50 sera from blood donors.

12 PERFORMANCE CHARACTERISTICS

12.1 Precision

The precision study was based on CLSI EP5-A2 *Evaluation of Precision Performance of Clinical Quantitative Measurement Methods.* The study was performed over 21 working days, with 2 runs per day. One user assessed 5 different samples, using 1 reagent lots on 3 analysers.

Precision Summary

	Mean (g/L)	Within run		Between run		Between day		Total	
		SD	CV %	SD	CV %	SD	CV %	SD	CV %
Level 1	0.0616	0.001	1.5	0.003	5.5	0.005	7.4	0.006	9.4
Level 2	0.1562	0.003	1.6	0.005	3.4	0.010	6.6	0.012	7.6
Level 3	0.2475	0.004	1.7	0.006	2.3	0.012	4.8	0.014	5.6
Level 4	0.4421	0.008	1.7	0.009	2.1	0.022	5.0	0.025	5.7
Level 5	0.8671	0.017	2.0	0.016	1.8	0.051	5.9	0.056	6.4

12.2 Comparison

A comparison study was performed by analysing 116 samples (66 normal sera and 50 clinical sera) using the Optilite Caeruloplasmin Kit and an alternative commercially available assay. Passing Bablok regression analysis generated the following results:

y = 1.07x – 0.01 (g/L) (y = Optilite; x = predicate analyser)

correlation coefficient r = 0.986 (calculated by linear regression)

A comparison study was performed by analysing 54 paired serum and lithium heparin plasma samples using the Optilite Caeruloplasmin Kit.Passing Bablok regression analysis generated the following results:

y = 0.97x – 0.00 (g/L) (y = lithium heparin plasma; x = serum)

correlation coefficient r = 0.979 (calculated by linear regression)

12.3 Limit of Quantitation

The limit of quantitation (LoQ) for this assay is defined as the bottom of the measuring range, 0.04 g/L. The LoQ validation study was based on CLSI EP17-A *Protocols for Determination of Limits of Detection and Limits of Quantitation.*

12.4 Linearity

A linearity study was performed following CLSI Evaluation of the Linearity of Quantitative Measurement Procedures: A Statistical Approach; Approved Guideline (EP6-A). The linearity of this assay has been confirmed using a serially diluted serum sample over the range of 0.04 – 0.82 g/L at Optilite analyser dilution 1+9 with deviation from linearity <10%.

12.5 Interference

A study was performed following CLSI EP7-A2: Interference Testing in Clinical Chemistry, Approved Guideline (CLSI Document EP7-A2). A normal serum sample, a sample close to the medical decision point and an abnormal serum sample were tested. No significant assay interference effects were observed when tested with Intralipid (100mg/dL), triglycerides (250mg/dL), bilirubin (200mg/L) or haemoglobin (5g/L).

12.6 Antigen excess

No antigen excess was observed up to a level of approximately three times the top of the calibration curve at the standard 1+9 sample dilution. This is equivalent to 3 g/L.

13 BIBLIOGRAPHY

1. Kodama, F, Fujisawa, C, Bhadhprasit, W. (2012) Inherited Copper Transport Disorders: Biochemical Mechanisms, Diagnosis and Treatment, *Current Drug Metabolism*, 13(3), 237-250.
2. Tietz Fundamentals of Clinical Chemistry 6th edn. (2008). Ed. C.A. Burtis and E.R. Ashwood, Publ. WB Saunders Company, Philadelphia, US, 300.
3. Tietz Clinical guide to laboratory tests, 4th edn. (2006). Ed. AHB Wu. Publ. WB Saunders Company, Philadelphia, US, 230.
4. Dati, F *et al.* (1996). Consensus of a Group of Professional Societies and Diagnostic Companies on Guidelines for Interim Reference Ranges for 14 Proteins in Serum based on the Standardization against IFCC/BCR/CAP Reference Material (CRM 470). International Federations of Clinical Chemistry. Community Bureau of Reference of the Commission of the European Communities. College of American Pathologists. Eur J Clin Chem Clin Biochem; 34:517-20.

14 DOCUMENT REVISION

Date of Issue	Revision detail
November 2022	Changes to the IFU are indicated with a vertical brace in the margin.

The Binding Site Group Ltd., 8 Calthorpe Road, Edgbaston, Birmingham, B15 1QT, UK. www.bindingsite.com
Telephone: +44 (0)121 456 9500
*Email: **info@bindingsite.com***

EC REP

The Binding Site Ireland Limited, First Floor, 43-49 Sir John Rogerson's Quay, Dublin 2, Ireland.
Telephone: +44 (121) 456 9500
*Email: **info@bindingsite.co.uk***

Optilite is a registered trademark of The Binding Site Group Limited (Birmingham, UK) in certain countries. Other brand or product names may be trademarks of their respective holders.

CE

15 SYMBOL INDEX

Symbol	Meaning	Symbol	Meaning
IVD	*In vitro* diagnostic medical device	REF	Catalogue number
	Manufacturer	CAL	Calibrator
EC REP	Authorised Representative in the European Community	CAL VAL	Calibrator Value
	Temperature limit	CONTROL	Quality Control
Σ	Contains sufficient for <n> tests	CONTROL RANGE	Control Range
	Consult instructions for use	CONTROL VAL	Control Value
	Use-by date	CONTROL H	High Control
LOT	Batch code	CONTROL L	Low Control

Binding Site
